# Breadth of ACE2 receptor usage predicts host range and antigenic relatedness across bat sarbecoviruses

**DOI:** 10.1371/journal.pbio.3003944

**Published:** 2026-10-01

**Authors:** Nazia Thakur, Joseph Newman, Dongchun Ni, Jeffrey Seow, Abigail L. Hay, Yeonjae Lee, Ayush Upadhyay, Babatunde E. Ekundayo, John A. Hammond, Thomas P. Peacock, Kelvin Lau, Katie J. Doores, Dalan Bailey

**Affiliations:** 1 The Pirbright Institute, Pirbright, Woking, United Kingdom; 2 Nuffield Department of Medicine, University of Oxford, Oxford, United Kingdom; 3 School of Pharmacy, International Cancer Center, Guangdong Key Laboratory of Genome Instability and Human Disease Prevention, Shenzhen University Medical School, Shenzhen, China; 4 Department of Infectious Diseases, School of Immunology & Microbial Sciences, King’s College London, London, United Kingdom; 5 Laboratory of Biological Electron Microscopy (LBEM), Institute of Physics, School of Basic Sciences, École Polytechnique Fédérale de Lausanne, Rte Cantonale, Lausanne, Switzerland; 6 Department of Infectious Disease, Imperial College London, London, United Kingdom; 7 Protein Production and Structure Core Facility (PTPSP), School of Life Sciences, École Polytechnique Fédérale de Lausanne, Lausanne, Switzerland; Washington University School of Medicine, UNITED STATES OF AMERICA

## Abstract

Sarbecoviruses interact with their receptor, angiotensin-converting enzyme 2 (ACE2), via the receptor-binding domain (RBD) of Spike, the immunodominant target for neutralising antibodies. Understanding the interplay and correlation between ACE2-determined host range and antigenicity is vitally important for understanding the zoonotic potential of related bat sarbecoviruses. Using binding assays, pseudotype-entry assays and a diverse panel of mammalian ACE2 proteins, we examined the host range and related antigenicity of multiple bat coronaviruses. Broad bat ACE2 usage (a *generalist* phenotype) was most common in clade 1 sarbecoviruses, including SARS-CoV-2 and the BANAL isolates from Laos. In contrast, clade 3 (e.g., RhGB07) and 5 (e.g., Rc-o319) sarbecoviruses exhibited more restricted ACE2 usage (a *specialist* phenotype). A novel structure for RhGB07 Spike further helped to identify RBD residues associated with this receptor specialism. Interestingly, the generalist phenotypes were largely maintained with more diverse mammalian receptor libraries, including human, non-human primate, livestock, rodent ACE2 and potential intermediate reservoir hosts (e.g., civet, racoon dog, pangolin), while specialists, like RhGB07, exhibited wider phenotypic diversity. The impact of SARS-CoV-2’s continued evolution in humans was also examined, identifying an expanding and/or shifting pattern of generalism for variants, especially Omicron and its sub-lineages. Furthermore, we compared and correlated these entry phenotypes with antigenicity using sera from SARS-CoV-2 convalescent individuals. Clade 1 viruses, phylogenetically related to SARS-CoV-2, were antigenically the most similar, with robust evidence for cross-neutralisation; however, there was still evidence for limited cross-neutralisation across the entire sub-genus. Finally, using monoclonal antibodies, derived from COVID-19 vaccinees with breakthrough infections, we pin-pointed the antibody epitope classes responsible for wider neutralisation. Our research indicates that generalist ACE2-using sarbecoviruses are phylogenetically and antigenically related to SARS-CoV-2.

## Abstract 2

How ACE2 receptor usage shapes sarbecovirus host range and antigenicity remains unclear. In a sarbecovirus screen against 34 ACE2 receptors, this study identifies viruses with generalist and specialist tropism, which correlates with antigenicity, with robust cross-neutralization of closely-related viruses but some cross-neutralisation across the entire viral sub-genus.

## Introduction

Severe respiratory coronavirus 1 (SARS-CoV-1) and more recently SARS-CoV-2, responsible for the COVID-19 pandemic, are both thought to have spilled over from *Rhinolophus* bat species, highlighting the significant risk of bat sarbecoviruses to human and animal health [1,2]. Our current understanding of the sarbecovirus sub-genus is that this group encompasses hundreds of related viruses, exhibiting high genetic diversity arising from recombination and multi-species host spillover events [1–3]. Phylogenetic analyses based on the receptor-binding domain (RBD) clusters the extant sequences into five distinct clades: clade Ia which includes SARS-CoV-1, clade Ib which includes SARS-CoV-2, and clades II-V; with the vast majority of sequences coming from bats [4–11]. The angiotensin-converting enzyme 2 (ACE2) protein has been identified as the receptor for many of these sarbecoviruses, with the exception of the clade II viruses, for which the receptor remains unknown [12]. To what extent human ACE2 usage is conserved across the sarbecovirus phylogeny is a source of intense investigation [13,14]. Although RaTG13, isolated from a *Rhinolophus affinis* horseshoe bat, is similar to SARS-CoV-2 at the RBD amino acid level (90%), we previously showed it does not share the same broad *generalist* ACE2 usage profile [15]. However, we identified mutations within the RaTG13 RBD-ACE2 interface that could increase human ACE2 (hACE2) usage, which were consistent with the mechanism of action underpinning the early adaptation of SARS-CoV-1 to civet and then human ACE2 [15,16]. Since 2020, multiple sarbecoviruses have been identified in bat reservoirs that already have high affinity for hACE2, for example, the BANAL viruses found in Laos (BANAL-20-52, BANAL-20-103, etc.) [17], raising intriguing questions about the molecular, ecological and evolutionary drivers for ACE2 generalism in bat reservoirs.

As the first step in the life cycle, receptor engagement is critical in determining virus host-range and species susceptibility. The identification of other human ACE2-using sarbecoviruses with generalist receptor usage profiles [10,13,14,18] is perhaps unsurprising, given the high sequence similarity of some these viruses [17]. However, to date, there have been no highly efficient (equivalent to SARS-CoV-2) human ACE2-using sarbecoviruses identified outside of clade 1. This underpins the need to better characterise the diversity of receptor usage across the whole sub-genera. Furthermore, how this spectrum of generalism and specialism correlates with bat ACE2 usage and bat ecology is also an important area for consideration. A previous receptor usage study using 46 ACE2 orthologues from phylogenetically diverse bat species, showed that 30 of these bat ACE2s were able to support entry of both SARS-CoV-1 and SARS-CoV-2. Further mutational analysis revealed that amino acid residues at positions 27, 31 and 42 in the bat ACE2 were important determinants of bat host-range [19]. RBDs from a more diverse range of sarbecovirus were shown to bind to various *Rhinolophus* ACE2 orthologues with high affinity, in particular clade Ia and Ib viruses [13]. Also, Roelle and colleagues, performing entry assays with chimeric sarbecovirus RBDs in a SARS-CoV-2 Spike backbone, detected entry in cells over-expressing human and *Rhinolophus* ACE2, including for clade III representatives BtKY72 and Khosta-2 [11]. Allied with this is a need to understand how host range and receptor tropism relates to antigenicity. For example, are there human ACE2-using generalist sarbecoviruses that are antigenically totally distinct from SARS-CoV-2? Related, does pre-existing immunity to SARS-CoV-2 protect against, or prevent, related bat sarbecoviruses emerging in the human population? Other studies have shown that monoclonal antibodies (mAb) derived from macaques immunised with SARS-CoV-2 subunit vaccines are able to neutralise clade I (SARS-CoV-1, WIV-1, SARS-CoV-2, Pangolin-GD) and clade III sarbecoviruses (BtKY72 and PRD-0038) [20]. However, a more detailed analysis of the breadth of COVID-19-derived immunity and the important epitopes that might define cross-protection is lacking. Another important aspect to consider is that over the course of the COVID-19 pandemic, SARS-CoV-2 itself has shifted species tropism [21,22]. Single amino acid changes in SARS-CoV-2 variant Spikes have expanded its tropism, e.g., to rodents (N501Y) and ferret/mink ACE2 (N501T) [21,23,24]. In addition, there are also examples of entirely new animal reservoirs being established, e.g., white-tailed deer, that are able to sustain onward transmission [25–27]. In many cases, this can likely be considered coincidental to the continued evolution of SARS-CoV-2 to human ACE2; however, the implications for bat ecology have perhaps been overlooked. Indeed, it is not clear whether novel SARS-CoV-2 variants have evolved enhanced tropism for bat ACE2 proteins. This might be of significance in biodiverse regions such as South America, where anthropogenic spillover (reverse zoonosis) of SARS-CoV-2 into native bat species is a conceivable risk.

To address these questions, we examined the receptor usage of a panel of 15 representative full-length bat sarbecovirus Spike proteins, using ACE2 libraries representing 16 bat species and a further 18 mammalian species of direct anthropogenic and/or zoonotic spillover relevance. We identified that clade Ia and Ib viruses widely, although not exclusively, exhibited generalist bat ACE2 usage, whereas clade III and V viruses were far more specialist, i.e., using only a limited number of receptors. A novel Spike trimer protein structure for one *specialist*, the UK-isolated RhGB07, identified several amino acid motifs that contribute to this host-range tropism. Interestingly, the patterns of generalism and specialism seen with bat receptors were largely mirrored when examining a wider mammalian library, a correlation which highlights that ecological and/or viral drivers of broad receptor usage tropism in bats could potentiate spillover risk into non-bat species. Significantly, bat ACE2 usage tropism has also shifted during the evolution of SARS-CoV-2 in human populations, highlighting underappreciated risks of reverse zoonosis. To broaden our understanding of the wider risk to human populations of future sarbecovirus spillover, we conducted serological assays using COVID-19 convalescent human sera as well as monoclonal antibodies derived from breakthrough infections. Importantly, it appears that all generalist ACE2 users are antigenically related to SARS-CoV-2. Somewhat surprisingly, even phylogenetically distinct, specialist ACE2-using sarbecoviruses still retained some antigenic relatedness to SARS-CoV-2, albeit with lower-level binding and neutralisation titres. This suggests a degree of cross-protection (against other sarbecoviruses emerging in human populations) might be conveyed by pre-existing COVID-19 immunity and outlines the potential of pan-sarbecovirus vaccines.

## Materials and methods

### Cells

HEK293T cells were used to generate lentiviral pseudoparticles, and BHK-21 cells for receptor screens. HEK293T cells stably expressing human angiotensin-converting enzyme 2 (HEK293T-hACE2) were used for pseudotype neutralisation assays. Cells were maintained in Dulbecco’s Modified Eagle Medium (DMEM, Sigma-Aldrich) supplemented with 10% FBS (Life Science Production), 1% 100mM sodium pyruvate (ThermoFisher Scientific), and 1% penicillin/streptomycin (10,000 Uml − 1; Life Technologies) at 37 °C in a humidified atmosphere of 5% CO_2_. Stable cell lines were generated, as described previously [28], using a lentiviral transduction system under 1 μg/mL puromycin (ThermoFisher Scientific) selection.

### Plasmids

Codon-optimised bat sarbecovirus Spike constructs were synthesised and subcloned into pcDNA3.1 (+) with a C-terminal FLAG tag (BioBasic, Canada). Human codon-optimised, Δ19-truncated Spike genes from RhGB07 and RfGB02 were subcloned into pcDNA3.1 (+) [10]. Mammalian ACE2 constructs were subcloned into pDISPLAY with an N-terminal signal peptide (the murine Ig k-chain leader sequence) and HA-tag (BioBasic, Canada; Twist Bioscience, USA). Accession numbers can be found in S1 and S2 Tables, respectively. All SARS-CoV-2 variant Spike expression plasmids were based on codon-optimised SARS-CoV-2 lineage B (Pango) reference sequence (GenBank ID NC_045512.2), with an additional Δ19 cytoplasmic tail truncation. Mutants of B.1 and BA.1, mutants of RhGB07, RBD chimeras of SARS-CoV-2 and RhGB07, and RBD chimeras of SARS-CoV-2 and Rs4231 were generated by site-directed mutagenesis, using the QuikChange II Lightning Multi Site-Directed Mutagenesis kit (Agilent).

### Lentiviral pseudotype production

Viral pseudotypes bearing the Spike of different bat sarbecoviruses were generated as described previously [29]. Briefly, HEK293T cells were transfected with p8.91 (gag/pol), CSFLW (lentivirus backbone expressing Firefly luciferase) and the Spike construct of interest using PEI transfection reagent (Merck). Virus was harvested 48 and 72 h post-transfection and centrifuged and frozen at −80 °C to remove cellular debris. Viruses were titrated on HEK293T-hACE2 stable cell lines, or where viruses used hACE2 poorly, HEK293T cells were transfected with the cognate bat ACE2 receptor (i.e., *Rh. cornutus* for Rc-o319, *Rh. ferrumequinum* for RhGB07 and RfGB02). Luciferase signals were measured 3 days later using Bright-Glo (Promega).

### ACE2 receptor screen

Receptor usage screens were conducted as described previously [15,21,29]. BHK-21 cells were seeded in 6-well plates at 7.5 × 10^5^/well in DMEM-10% 1 day prior to transfection with 500 ng of different species, ACE2-expressing constructs or empty vector (pDISPLAY) (S2 Table) in OptiMEM (ThermoFisher Scientific) and TransIT-X2 (Mirus Bio) transfection reagent, according to the manufacturer’s recommendations. The next day, cells were re-plated into white-bottomed 96-well plates at 2 × 10^4^/well and infected with pseudoparticles equivalent to 10^5^ to 10^6^ relative light units (RLU), pre-determined by titrating on BHK-21 cells overexpressing *Rh. cornutus* ACE2 (Rc-o319), *Rh. alcyone* ACE2 (RfGB02, RhGB07) or human ACE2 (all others) and the volume of pseudoparticles added to the assay was adjusted accordingly. For non-ACE2-using pseudoviruses, a fixed volume of 100 µL was used in subsequent assays. Transfected cells were incubated with pseudotyped virus for 48 h at 37 °C, 5% CO_2_. To quantify Firefly luciferase, media was replaced with 50 μL Bright-Glo substrate (Promega) diluted 1:2 with PBS. The plate was incubated in the dark for 2 min and then read on a Glomax Multi+ Detection System (Promega) as above. Comma-separated values (CSV) files were exported for analysis. Biological replicates were performed 3 times.

### Micro virus neutralisation assays

Neutralisation assays were conducted as described previously [30]. COVID-19 convalescent plasma/serum were diluted in serum-free media for a final dilution of 1:40 and added in triplicate to a white-bottomed 96-well plate and titrated 3-fold. Serial dilution of mAb were prepared. Convalescent plasma/serum or mAbs were incubated with a fixed titred volume of sarbecovirus pseudoparticles equivalent to 10^5^–10^6^ signal luciferase units and incubated for 1 h at 37 °C and 5% CO_2_. Target HEK293T cells expressing human ACE2, or the relevant bat ACE2 receptor (*Rh. alcyone* for RhGB07 and RfGB02, *Rh. cornutus* for Rc-o319) were then added (at a density of 2 × 10^5^ cells/mL for convalescent serum/plasma, and at 4x10^5^cell/mL for mAbs) and incubated at 37 °C, 5% CO_2_ for 72 h. Firefly luciferase activity was then measured after the addition of Bright-Glo luciferase reagent on a luminescence plate reader (GloMax-Multi+ Detection System (Promega) for convalescent serum/plasma or Victor X3 multilabel plate reader (PerkinElmer) for mAbs). Pseudotyped virus neutralisation titres were calculating by interpolating the point at which there was a 50% reduction in luciferase activity, relative to untreated controls (IC_50_).

### Cell–cell fusion assays

HEK293T rLuc-GFP 1–7 [28] effector cells were transfected in OptiMEM (ThermoFisher Scientific) using Transit-X2 transfection reagent (Mirus Bio), as per the manufacturer’s recommendations, with sarbecovirus Spikes (500ng) alongside mock-transfection with empty plasmid vector (pcDNA3.1+) (S1 Table). BHK-21 rLuc-GFP 8–11 target cells were transfected with 500 ng of different ACE2-expressing constructs (S2 Table). Effector and target cells were co-cultured at a ratio of 1:1 in white 96-well plates to a final density of 4 × 10^4^ cells/well 2 days post-transfection. Quantification of cell–cell fusion was measured based on Renilla luciferase activity, 24 h later by adding 1 μM of Coelenterazine-H (Promega) at 1:400 dilution in PBS. The plate was incubated in the dark for 2 min and then read on a Glomax Multi+ Detection System (Promega) as above and CSV files were exported for analysis. To quantify GFP expression, co-cultures were set up of effector and target cells as above into a clear flat-bottomed 96-well plate (Nunc) and imaged 24 h later using the IncuCyte S3 live cell imaging system (Essen BioScience). Four fields of view were taken per well at 10× magnification. Analysis of images generated was performed using the IncuCyte S3 software, setting fluorescence (“masks”) and cellular thresholds (“objects”) to remove background fluorescence. The total sum of objects with fluorescent masks was then calculated using the total integrated intensity metric, expressed as green count units (GCU) µm^2^.

### Recombinant protein production

Secreted sarbecovirus RBDs and mammalian ACE2 proteins were produced by cloning into the pOPIN-BAP-His and pOPIN-Fc expression vectors, respectively. These plasmids were then expressed in Expi293F cells using the PEI40K transfection reagent (Polysciences) with additives (300 mM valproic acid, 500 mM of sodium propionate, 2.5 M glucose). Supernatants were harvested 4 days post-transfection, centrifuged for 45 mins at 6,000*g* and filter through a 0.45 µM filter. Presence of secreted protein was determined by Coomassie and by blotting for anti-His or anti-Fc. Supernatants were purified using HisTrapHP or HiTrap Protein A columns (Cytivia), desalted using Zeba Spin Desalting Columns (ThermoFisher Scientific) and concentrated using Amicon Ultra 15 columns (Merck Millipore, #C7715). The proteins were then quantified using a Nanodrop and a Pierce BCA Protein assay (ThermoFisher Scientific) and run on an SDS-PAGE gel to assess protein purity by Coomassie.

### Structural analysis

#### Protein production.

For protein production, this was performed as previously reported for Spike and ACE2 [10]. Briefly for Spike, a CHO-codon-optimised sequence of the soluble ectodomain of RhGB07 (residues 1–1,191) was placed downstream of the u-phosphatase signal secretion peptide. Following the Spike sequence, a C-terminal T4-foldon trimerization domain, a 3C-protease cleavage site, 8x-His tag and a 2× Strep tag were included. A ×putative polybasic site RAK (residues 669–671) was mutated to GAS. A prefusion-state 2P-stabilising mutation was also added (residues 969–970). The protein was produced ExpiCHO cells and purified via the 2× Strep tag. For ACE2 expression and purification were also performed in the same manner as Spike.

#### Cryo-electron microscopy.

Cryo-EM grids were prepared as previously [31] on a Vitrobot Mark IV (Thermofisher Scientific (TFS)). Quantifoil R1.2/1.3 Au 400 holey carbon grids were glow-discharged for 90 secs at 15mA using a PELCO easiGlow device (Ted Pella). The putative complex was formed by mixing 3µM of RhGB07 spike with 6 µM of either human or *Rh. ferrumequinum* ACE2 and applying 3 µL to the glow-discharged grids blotting for 6 secs, blot force 10, at 95% humidity and 10 °C in the chamber. The blotted grid was plunge-frozen in liquid ethane cooled by liquid nitrogen.

#### Data screening, collection and processing.

On a TFS Titan Krios G4, the grids were screened and only one was suitable for data collection containing the putative RhGB07 Spike with human ACE2. On-the-fly processing on cryoSPARC live v3.3.2 was used during data acquisition (10.1038/nmeth.4169). Pre-processed motion-corrected micrographs were patch-corrected and used thereafter. 5,254,208 particles were template-picked using PDB: 7QO7 as a template. After 2 rounds of 2D classification, clear classes showing a trimeric spike were observed comprising 2,685 particles. The 2D classes already show a trimeric spike resembling a closed conformation. These particles were sufficient to produce an ab initio model and can be refined to 3.66 Å with C3 symmetry. To increase the resolution and quality of the map, these 2D classes were used once again for template picking which led to 2,234,763 particles. After several rounds of extracting, cropping (Box size 500 pixel, Crop size 300 pixel) and classification only 21,821 particles remained (3.09 Å, C3 symmetry) and were subjected to heterogenous refinement (C1 symmetry) with 4 classes. The 3 best classes in terms of resolution were used (17,685 particles) for a final round of NU-Refinement leading to a map of 2.97 Å (FSC 0.143, C3 symmetry). This map showed good local resolution at the RBD without necessitating for local refinement for this domain. Due to cropping during particle extraction, the FSC curve is truncated at the Nyquist limit of 2.77 Å and does not fully decay to zero. The atomic model, its associated map, and the raw motion-corrected data has been deposited under PDB: 9RDT, EMDB: 53938 EMPIAR: 12921. A map generated from the same particles with no cropping during particle extraction shows a full decay to zero in its FSC curve and has also been deposited under EMDB: 58663.

#### Model building.

The final model was built by docking Alphafold2 [32] predictions as individual domains (NTD, RBD, S2) using phenix.voyager [33] cycles of manual model-building in COOT [34], specifically within the NTD, RBD and the putative basic region of the RhGB07. Analysis and figures were done within ChimeraX [35].

### Surface plasmon resonance

Kinetics and epitope binning assays were performed on the Carterra LSA^XT^ using HC30M chips. Chips were pre-conditioned using sequential 60s injections of 50 mM NaOH, 1 M NaCl, and 10 mM pH2 glycine. For the kinetics assay, an anti-human Fc lawn was prepared by covalently coupling goat anti-human-Fc polyclonal antibody (BioRad 5211-8004) to the chip surface*.* Free carboxyl groups on the chip surface were activated for 5 min using 1-ethyl-3-(3-dimethylaminopropyl)carbodiimide hydrochloride (EDC) and 115 mg N-hydroxysuccinimide (NHS) (final concentrations of 130 and 33 mM, respectively, in PBS). Goat anti-human-Fc was diluted to 50 μg/ml in 10 mM sodium acetate (NaOAc) to pre-concentrate the protein into the negatively charged mix and applied to the chip for 7 min in two independent locations. The coupling reaction was quenched for 5 min using 500 mM ethanolamine, and the chip washed with three 30-second pulses of pH 1.9 glycine (10 mM). The human anti-RBD monoclonal antibodies were diluted in PBST (PBS + 0.005% tween 20) to 1 µg/ml and 0.25 µg/ml and captured to the anti-human-Fc lawn for 7 min. Non-regenerative kinetics was performed using RBD diluted 2-fold in PBST (5 μg/ml to 2.4 ng/ml) allowing a seven-minute association and 14- minute dissociation. After the highest concentration of RBD the anti-human Fc lawn was regenerated using three 30-second injections of pH 1.9 glycine (10 mM) and fresh human anti-RBD antibodies were captured for each sarbecovirus RBD kinetics cycle. Kinetic analysis was performed in Kinetics v1.9.2 to calculate K_D_ by fitting to the 1:1 model following channel and double referencing. Average K_D_ was calculated using the values obtained from both coupling concentrations and the two independent couplings on the chip giving *n* = 4.

For epitope binning, human anti-RBD monoclonal antibodies were diluted to 1 μg/ml in NaOAc and directly coupled to the pre-conditioned HC30M chip as above allowing a 10-min coupling period. SARS-CoV-2 wild-type RBD was diluted to 0.625 μg/ml in PBST and injected for 5 min, followed by the competing antibody at 1 μg/ml for 5 min. The chip was regenerated using pH 1.9 glycine (10 mM) as previously before injecting the next RBD and competitor antibody. Epitope binning and community analysis was performed in Epitope v1.9.2.

### Flow cytometry

BHK-21 cells were transfected using Transit-X2 transfection reagent (Mirus Bio), as per the manufacturer’s instructions with 500 ng of each ACE2-expressing construct (S2 Table) or mock-transfected with empty plasmid vector (pDISPLAY) for 48 h. Cells were resuspended in cold PBS and washed in cold stain buffer (PBS with 1% BSA (Sigma-Aldrich), 0.01% NaN_3_ and protease inhibitors (ThermoFisher Scientific). Cells were stained with anti-HA Phycoerythrin (PE)-conjugated antibody (Miltenyi Biotech) at 1:500 dilution for 10^6^ cells for 30 min on ice. In parallel, 10 μg RBD recombinant proteins were stained with anti-His-Allophycocyanin (APC)-conjugated antibody (Miltenyi Biotech) at 1:500, and 50 µl of stained RBD were mixed with cells for 1 h to allow binding. Cells were then fixed with 2% paraformaldehyde (Fisher Scientific) on ice for 20 mins, then resuspended in PBS before being analysed using the MACSQuant Analyzer 10 (Miltenyi Biotech), and RBD-ACE2 binding was statistically calculated by SE Dymax % Positive. The mean SE Dymax % Positive was calculated from three biological replicates and analysed using FlowJo_v10.8.1. The gating strategy can be found in S4A Fig and the same gating strategy was applied in all experiments.

### Western blotting

BHK-21 cells were transfected using Transit-X2 transfection reagent (Mirus Bio), as per the manufacturer’s instructions with 500 ng of different ACE2-expressing constructs (S2 Table) or mock-transfected with empty plasmid vector (pDISPLAY) and harvested 48 h post-transfection. Pseudotyped virus was purified and concentrated under a 20% sucrose cushion by ultracentrifugation (23,000 rpm for 2 h, 4 °C). All protein samples were generated using 2 × Laemmli buffer (Bio-Rad) and reduced at 95˚C for 5 min. Samples were resolved on 7.5% acrylamide gels by SDS-PAGE, using semidry transfer onto nitrocellulose membrane. Blots were probed with anti-HA primary antibody (Cell Signalling, #2367) for ACE2-transfected cells at 1:1,000 or with anti-GAPDH (Cell Signalling, #2118) internal control at 1:3000. Purified psuedovirus was resolved using an anti-FLAG primary antibody (Cell Signalling, #2368) at 1:1,000, anti-Spike antibody (Novus Biologicals, #NB100-56578) at 1:500 or anti-p24 (ThermoFisher Scientific, #MA1-71515) as an internal lentiviral control at 1:1,000 in PBS-Tween 20 (PBS-T, 0.1%) with 5% (w/v) milk powder overnight at 4˚C. Blots were washed in PBS-T and incubated with anti-mouse (for HA and p24) or anti-rabbit secondary (for GAPDH, Spike and FLAG), antibody conjugated with horseradish peroxidase (Cell Signalling, #7076 and #7074, respectively) at 1:3,000 in PBS-T for 1 h at room temperature. Membranes were exposed to Clarity Western ECL substrate (Bio-Rad) according to the manufacturer’s guidelines and exposed to autoradiographic film.

### RBD ELISA

Recombinant RBD was coated onto 96-well ELISA plates at 1 µg/ml per well in carbonate/bicarbonate coating buffer (0.6 M pH9.6) at 4–8 °C overnight. Plates were blocked for 1 at RT with PBS-T containing 1% BSA, after which a 3-fold dilution series of monoclonal antibodies (starting concentration 5 µg/ml) or polyclonal COVID-19 plasma/serum (starting concentration 50 µg/ml), or a 4-fold dilution series of ACE2 recombinant proteins (starting concentration 8 µg/ml), were diluted in PBS and added for 1 h, at 37 °C. Plates were washed 3 times with PBS-T, followed by the addition of anti-human-Fc HRP conjugate diluted 1:10,000 added for 1 h at 37 °C. 1-step Ultra TMB (3,3′,5,5′- 40 Tetramethylbenzidine) (Thermo Fisher Scientific) was added to each well, incubated for 5–10 min at RT and the reactions stopped with an equivalent volume of 1 M Sulphuric acid solution, after which the optical density (OD) at 450 nm was measured using a GloMax Microplate Reader (Promega). Samples were analysed by plotting 50% effective concentration (EC50) using GraphPad Prism (8.2.1) software.

## Results

### Different bat Sarbecovirus clades show varied bat ACE2 usage

Sarbecoviruses have been isolated from different bat species across Afro-Eurasia, phylogenetically clustering into established virus clades, when aligned using the RBD amino acid sequences. Representative viruses from each clade were selected for analysis based on sequence availability and experimental data in publications [5,6,12,17,36] (Fig 1A; highlighted in red). This included the clade Ia viruses SARS-CoV-1, WIV-1 and Rs4231 (yellow), clade Ib viruses SARS-CoV-2, RaTG13, BANAL-20-52, BANAL-20-236 and BANAL-20-103 (orange), clade II viruses RacCS203, RpYN06, Rf1/2004 and Rp3/2004 (blue), clade III viruses RhGB07, RfGB02 (green), and the clade V virus Rc-o319 (red). Clade IV viruses were not included in this study. The selected viruses have been isolated primarily in Asia (clade I, II, IV and V), and in parts of Europe and Africa (clade III), as indicated (Fig 1B). The bat species from which these viruses were isolated are globally widespread (individual geographic distributions of each species are shaded as shown; overlay in Fig 1B and individually in S1 Fig). Of note, Rhinolophus species*, Pteropus giganteus, Lyroderma lyra, Rousettus leschenaultii and Taphozous melanaopogon* bat species overlap in their geographic reach, particularly in Southeast Asia, whereas the *Desmodus rotundus, Antrozous pallidus and Myotis lucifugus* species intersect in their range only in the Americas (Fig 1B).

**Fig 1:**
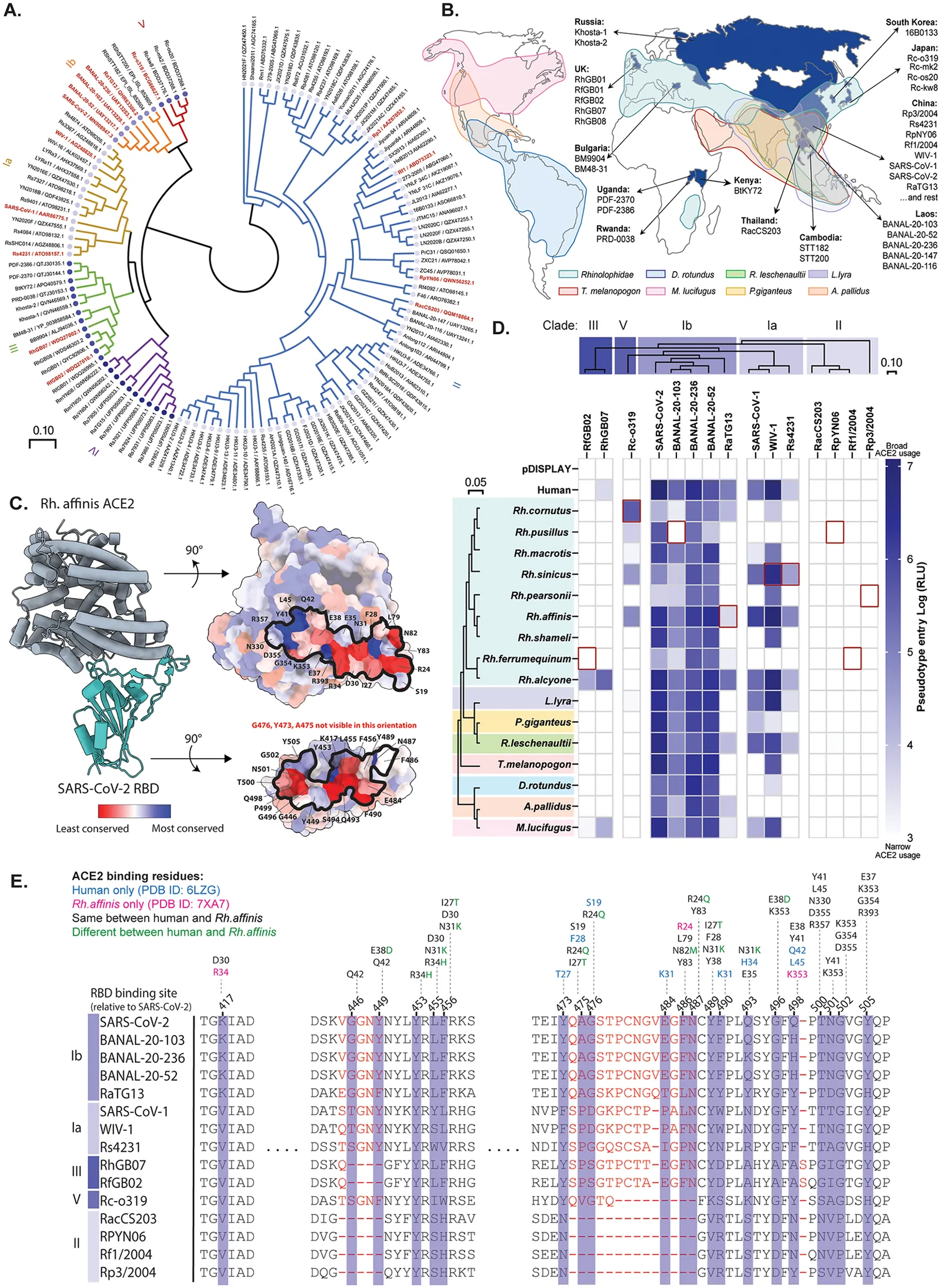
Phylogenetic and geographic distribution of bat sarbecoviruses and their bat ACE2 tropism. **(A)** Evolutionary history of bat sarbecovirus RBDs (NCBI reference shown for 124 amino acid sequences) was inferred by using the Maximum Likelihood method and Whelan and Goldman model. Initial tree(s) for the heuristic search were obtained automatically by Neighbour-Join and BioNJ algorithms to a matrix of pairwise distances (225 total positions) estimated using the JTT model with discrete Gamma distribution (5 categories (+G, parameter = 0.3524)), and then selecting the topology with superior log-likelihood value. Evolutionary analyses were conducted in MEGA 11. Bat sarbecovirus RBDs cluster into clades: clade Ia (yellow), clade Ib (orange), clade II (blue), clade III (green), clade IV (purple) and clade V (red). Viruses selected for analysis in this study are highlighted in red. **(B)** World map highlighting the geographic location where sarbecoviruses investigated in this study were first isolated (coloured in blue), along with the distribution of the bat species these were isolated from (https://www.iucnredlist.org/). **(C)** Structural images showing the binding of *Rhinolophus affinis* ACE2 with SARS-CoV-2 RBD (PDB 7XA7). Offset images highlight the conserved amino acid residues across the different bat ACE2s relative to *Rh. affinis* ACE2, and the conservation of bat sarbecovirus RBDs compared to SARS-CoV-2, screened in this study. The ACE2-RBD interacting residues are denoted. **(D)** Maximum likelihood phylogeny of the bat sarbecoviruses full-length Spike and bat ACE2 amino acid sequences are shown. Bat sarbecovirus Spikes were used to generate lentiviral-based pseudotypes and used to infect BHK-21 cells overexpressing different bat ACE2s or human ACE2. A heatmap illustrating receptor usage is shown, representing the mean log RLU of 3−5 separate experiments. A vector-only control (pDISPLAY) was included to demonstrate specificity and to set the background signal. The cognate bat receptor for each virus, where possible is highlighted with a red box. Absence of a red square is indicative of the lack of cognate receptor from our screen, either due to unavailability of ACE2 sequence, or because the original bat host is yet unknown (RhGB07: *Rh. hipposideros*; SARS-CoV-2, SARS-CoV-1: unknown; BANAL-20-52: *Rh. malayanus*; BANAL-20-236: *Rh. marshalli*; RacCS203 – *Rh. acuminatus*). **(E)** Sequence alignment of bat sarbecovirus RBDs at the ACE2-binding interface, with key amino acid residues denoted, using SARS-CoV-2 numbering. Regions of amino acid deletions between sequences are denoted in red. The corresponding human and *Rh.affinis* ACE2 residues that bind SARS-CoV-2 RBD are highlighted, with black indicating amino acid residues that are the same between human ACE2 and *Rh. affinis* ACE2, green indicating the same residues but a different amino acid, and the remainder highlighting unique binding residues for human ACE2 (blue) or *Rh. affinis* ACE2 (pink) only. The data underlying this Figure can be found in S1 Data.

To understand how bat ACE2 tropism varies across the sarbecovirus sub-genus, a receptor usage screen was conducted in cells overexpressing human ACE2 (hACE2) or 16 different bat ACE2 receptors, which included a range of *Rhinolophus* species (*Rh. cornutus, Rh. pusillus, Rh. macrotis, Rh. sinicus, Rh. pearsonii, Rh. affinis, Rh. shameli, Rh. ferrumequinum, Rh. Alcyone*) and representative bat species from other families (*L. lyra, P.giganteus, R. leschenaultii, T. melanopogon, D. rotundus, A. pallidus and M. lucifugus)*. When their sequence conservation was analysed in the context of the structure of SARS-CoV-2 RBD bound to *Rh. affinis* ACE2 (PDB 7XA7), the selected bat sarbecoviruses and bat ACE2s showed high levels of divergence at the binding interface, suggesting that the receptor usage of these viruses with bat ACE2s assessed in this study would likely vary (Fig 1C). Transfected cells were subsequently transduced with a fixed volume of bat sarbecovirus Spike-based pseudotypes equivalent to 10^5^–10^6^ RLU (those highlighted in red in Figs 1A and S2A; S1 and S2 Tables; S1 Data). Expression of ACE2 proteins in transfected cells, as well as SARS-CoV-2 Spike and p24 (internal lentivirus control) in purified pseudotypes, was confirmed in parallel (S2B and S2C Fig). To summarise, a diverse pattern of receptor usage was seen across the viruses tested (Fig 1D; S1 Data). Spike proteins from clade Ib sarbecoviruses, which includes SARS-CoV-2, had a broader (more *generalist*) receptor usage pattern, with RaTG13 being a notable exception. Indeed, only a few species of bat ACE2s were less permissive to SARS-CoV-2 or BANAL Spike-mediated entry. Clade Ia, which includes SARS-CoV-1, had a more intermediate receptor usage pattern, with clearer examples of restriction, even within the Rhinolophus bat family, e.g., *Rh. ferrumequinum* and *Rh. pusillus*. Nevertheless, WIV-1 and Rs4231, which were both isolated from *Rh. sinicus*, showed robust usage of this ACE2 receptor. In contrast, the clade III and V Spikes exhibited more restricted (*specialist*) ACE2 usage, primarily using the cognate bat ACE2 from which they were isolated, e.g., *Rh. cornutus* ACE2 usage by Rc-o319. As expected, the clade II viruses were unable to use any of the bat ACE2 receptors tested (Fig 1D, S1 data) [1,2,5,18,37,38]. Sequence alignment of the bat sarbecovirus RBDs, relative to SARS-CoV-2, revealed various deletions: at positions 446−449 in clade III viruses, at positions 480−487 for clade V viruses, and at positions 445−449 and 475−487 for clade II viruses (equivalent SARS-CoV-2 Wu-hu-1 numbering used throughout this study). The RBD-ACE2 interaction sites of *Rh. affinis* and human ACE2 were also compared based on known structures (human ACE2: PDB 6LZG, *Rh. affinis* ACE2: PDB 7XA7), noting differences in amino acid residues at positions 24, 27, 31, 34, 38 and 82. We also noted unique binding sites for human ACE2 that were not present for *Rh. affinis* ACE2, and vice versa (Fig 1E).

Coronavirus Spike proteins are also capable of inducing cell-cell fusion. To examine concordance between pseudotype entry and fusion, we repeated the assays in a quantitative cell-cell fusion assay, showing similar trends in quantifiable luciferase signals and GFP^+^ syncytia formation (S3A and S4 Figs; S1 Data). Our cell-cell fusion assay also showed high concordance with the pseudotyped-based entry assay by Pearson’s correlation (S3B Fig; S1 Data). The entry of bat sarbecoviruses with bat ACE2s also correlated to protein binding and affinity, i.e., those viruses that showed the highest levels of virus entry also bound most strongly in RBD-based flow cytometry assays (S5A and S5B Fig). Further, purified recombinant bat sarbecovirus RBDs were used to assess binding to recombinant human ACE2 and two bat ACE2s (*Rh. cornutus, Rh. leschenaultti*) in ELISA; however, these assays appeared to be less sensitive and do not allow determination of affinity, rather provide an approach to relatively rank binding across ACE2 orthologues (S5C Fig; S1 Data). Recombinant SARS-CoV-2 and RhGB07 RBDs were also used to assess binding to the three ACE2 species for which RhGB07 entry was observed (human, *Rh. Alcyone*, *M. lucifugus*). We identified good binding of SARS-CoV-2 RBD to these ACE2s, which was markedly reduced for RhGB07 RBD, correlating to the pseudotype entry phenotypes (Figs 1D and S5D; S1 Data).

### RhGB07 structure reveals a modified putative RBD-ACE2 interaction interface

Our functional screens revealed interesting differences between viruses within the same clade, particularly clade III viruses. For example, although RhGB07 exhibited a specialist receptor usage profile, it was able to enter cells overexpressing human ACE2, whereas RfGB02 could not (Fig 1D). To understand the structural determinants of generalism and specialism, as well as zoonotic potential across sarbecoviruses we therefore focused on resolving the structure of the specialist Spike, RhGB07. Previously, we produced recombinant 2P RhGB07 Spike with a putative basic region mutated and tested this in BLI binding assays [10]. To reveal the molecular details of potential interactions between ACE2 and RhGB07 Spike, we attempted to determine a cryoEM structure of human ACE2 and RhGB07 Spike. We prepared grids for cryoEM by co-incubating either human or bat ACE2 (*Rh. ferrumequinum*) with RhGB07 Spike before freezing. Initial representative 2D classes did not reveal the presence of any bound ACE2 to Spike (S6A Fig; S3 Table). Further classification revealed the presence of only 3-RBD closed/down Spike particles (in the dataset containing hACE2 and RhGB07). We could clearly define a limited number of particles and when C3 symmetry was imposed we could refine a map to 2.97 Å (S6A Fig). The model was built by docking Alphafold2 predictions as individual domains using phenix.voyager followed by manual model-building, specifically within the NTD, RBD and the putative basic region of the RhGB07. The structure revealed a trimeric organisation of protomers, following a domain organisation typical of sarbecoviruses. As noted in the 2D classes, the structure revealed a completely closed structure that shows no variation between RBD up or down (Fig 2A).

**Fig 2:**
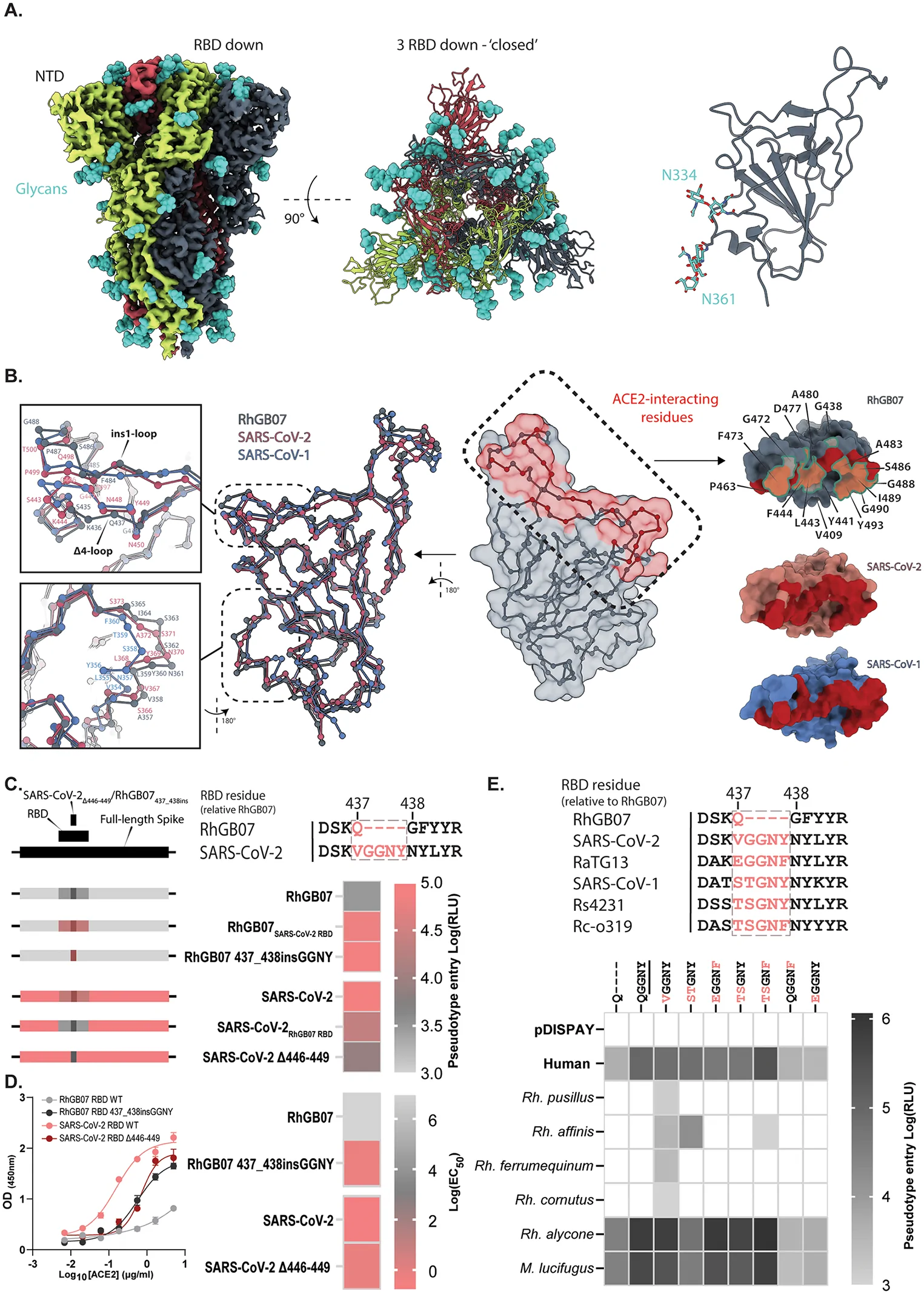
Cryo-EM structure of RhGB07 trimeric spike in a 3-RBD down state. **(A) Left:** CryoEM density of the trimer spike coloured by the protomer chains the density originates from. Glycans are highlighted in teal. **Centre:** the trimeric spike viewed from the top rendered in cartoon representation. **Right:** the isolated RhGB07 RBD represented as cartoon in grey with two glycans of interest shown. **(B)** The C-alpha trace of the RBD of RhGB07 rendered as balls-and-sticks superimposed with the RBD of SARS-CoV-1 (PDB 7DF3) and SARS-CoV-2 (PDB 5X5B). The insets show a zoomed-in view of two regions of the RBD that show the greatest changes in the position of the C-alpha of the RBD. The region containing the ins1-loop and delta4-loop coincides with putative ACE2-interacting residues based on mapping the equivalent residues of SARS-CoV-1 and SARS-CoV-2. The ACE2-interacting residues were determined by superimposing SARS-CoV-1 and RhGB07 RBD onto the SARS-CoV-2 RBD – ACE2 (PDB: 6M0J) structure and identifying interacting residues. Highlighted residues of RhGB07 show the putative interacting residues that differ in identity to SARS-CoV-2. **(C)** Schematic depicting mutants generated of RhGB07 and SARS-CoV-2 Spikes with either full RBD swaps, or insertions/deletions, respectively. Lentiviral pseudotypes bearing Spikes of RhGB07 or SARS-CoV-2 mutants (RhGB07 wildtype, RhGB07_SARS-CoV-2 RBD_, RhGB07437_438insGGNY, SARS-CoV-2 wildtype, SARS-CoV-2_RhGB07 RBD_, SARS-CoV-2 Δ446−449), were used to infect BHK-21 cells overexpressing human ACE2. A heatmap illustrating entry is shown, representing the mean log RLU of 3 separate experiments. **(D)** ELISA data depicting the binding curves and calculated EC_50_ values (µg/ml) of recombinant RhGB07, SARS-CoV-2, RhGB07 437_438insGGNY or SARS-CoV-2 Δ446−449 RBDs with recombinant human ACE2. **(E)** Alignment of the Δ4-loop region of RhGB07 with SARS-CoV-2, SARS-CoV-2, RaTG13, Rs4231, and Rc-o319 (RhGB07 numbering), with differences in amino acid residues shown. RhGB07 was mutated to introduce an insertion (437_438insGGNY, underlined) or additional mutations (mutated residues coloured in pink) in the Δ4-loop. A heatmap illustrating entry data is shown for pseudoviruses bearing these mutant Spikes, used to infect BHK-21 cells overexpressing *Rh pusillus*, *Rh. affinis*, *Rh. ferrumequinum*, *Rh. cornutus*, *Rh. alcyone and M. lucifugus* ACE2s. Data represents the mean log RLU of 4 separate experiments. A vector-only control (pDISPLAY) was included to demonstrate specificity and to set the background signal. The data underlying this Figure can be found in S1 Data.

Comparison with other Clade III RBD apo-structures, PRD-0038, Khosta-1, Khosta-2 and BtKY72 revealed high similarity between the C-alpha traces (S7A and S7B Fig). However, regions of differences were noted that coincide with putative ACE2-interacting residues based, particularly in the ins1-loop and Δ4-loop, which could impact ACE2 usage and receptor tropism. It was also noted that the closed-state PRD-0038 RBD structure resembled SARS-CoV-2 RBD with a fatty acid, linoleic acid, bound [14]. An examination of the RhGB07 RBD revealed no observable density (S7C Fig) [39]. Similarly, the NTD, that in some clade 3 binds biliverdin, also did not reveal any density (S7C Fig) [40]. Comparisons of the sequence conservation between RhGB07 and the other bat sarbecovirus Spikes examined in this study revealed the RBD and NTD as regions of highest variability (S7D Fig). Further comparisons between the structures of the generalist sarbecovirus Spikes from SARS-CoV-1 and SARS-CoV-2 with RhGB07 revealed structural similarity except at the sites of deletion and insertions (Fig 2B). Within the RBD, we determined the loops that mediate ACE2 interaction in SARS-CoV-1 and SARS-CoV-2 adopt strikingly different 3D conformations as compared to RhGB07 and also contain residues of different identities to RhGB07 despite the overall RBD structure appearing highly identical (Fig 2B). For example, the ‘ins1-loop’ where there is a single residue insertion in RhGB07 compared to SARS-CoV-2, positions serine 486 (RhGB07 numbering) in place of the Q498 residue in SARS-CoV-2. Changes at these hotspot loops may be determinants for RhGB07-ACE2 binding selectivity.

To examine the extent to which the Δ4-loops contribute to the restricted receptor tropism of RhGB07, mutants of RhGB07 and SARS-CoV-2 were generated for comparison. Firstly, full RBD chimeric swaps were generated, resulting in, as expected, a modest increase in human ACE2 usage for RhGB07_SARS-CoV-2 RBD_ pseudotypes, and a slight decrease in pseudotype entry for SARS-CoV_RhGB07 RBD_ (Fig 2C; S1 Data). Next, the direct importance of the Δ4-loop was investigated by inserting the SARS-CoV-2 GGNY residues between positions 437 and 438 in the RhGB07 Spike (RhGB07 437_438insGGNY) or deleting the corresponding region in SARS-CoV-2 (SARS-CoV-2 Δ446−449). A further decrease in human ACE2 usage was noted with the SARS-CoV Δ446−449 mutant, and a similar human ACE2 usage for RhGB07 437_438insGGNY, when compared to RhGB07_SARS-CoV-2 RBD_ (Fig 2C; S1 Data). This indicates that this region is important in mediating virus entry, likely due to altered ACE2 binding affinity, confirmed by ELISA (Fig 2D; S1 Data). To examine the wider significance of this result to sarbecovirus biology, further mutants of RhGB07 were generated to examine whether host range could also be altered by mutating the Δ4-loop region to reflect other generalist sarbecoviruses. RhGB07 was mutated to make it more SARS-CoV-2-like (RhGB07 437_438insVGGNY), SARS-CoV-1-like (RhGB07 437_438insSTGNY), Rs4231-like (RhGB07 437_438insTSGNY), RaTG13-like (RhGB07 437_438insEGGNF), or Rc-o319-like (RhGB07 437_438insTSGNF). Importantly, the receptor tropism of these mutants was examined with a small panel ACE2s that were previously shown to be differentially used by our bat sarbecovirus (Fig 1D). The use of human, *Rh.alycone* and *M. lucifugus* ACE2s increased with all mutants except the SARS-CoV-1-like mutant (RhGB07 437_438insSTGNY), which remained similar to wildtype RhGB07 (Fig 2E; S1 Data). Interestingly, the RhGB07 437_438insGGNY mutation alone was insufficient to broaden bat ACE2 tropism, but additional mutations to make the virus SARS-CoV-2-like (RhGB07 437_438insVGGNY) (generalist), increased usage of *Rh. pusillus*, *Rh affinis*, *Rh. ferrumequinum* and *Rh. cornutus* ACE2s (Fig 2E; S1 Data), highlighting the importance of the Q437V substitution. Also of note, an increased usage of *Rh. affinis* ACE2 was observed with the SARS-CoV-1-like mutant (RhGB07,437_438insSTGNY), but not the other bat ACE2s examined, mirroring the ACE2-usage profile of SARS-CoV-1 (Fig 1D). However, while the Rc-o319-like mutant (RhGB07 437_438insTSGNF) also exhibited an increase in *Rh. affinis* ACE2 usage, this was not observed for the RaTG13-like mutant (RhGB07 437_438insEGGNF) or Rs4231-like mutant (RhGB07 437_438insTSGNY), despite Fig 1D showing both RaTG13 and Rs4231 sarbecovirus pseudotypes are able to enter cells overexpressing *Rh. affinis* ACE2 (Fig 2E; S1 Data). Additionally, RhGB07 437_438QGGNF and RhGB07 437_438insEGGNY mutants alone (reflecting the Δ4-loop region sequences of RaTG13 and Rc-o319) were unable to increase receptor tropism, and in fact exhibited a decrease in human, *Rh. alcyone* and *M. lucifugus* ACE2 usage (Fig 2E; S1 Data). These data indicate a synergistic role for these residues in the Δ4-loop, but also the likely involvement of other residues in the RBD or Spike that contribute to receptor tropism.

Together, these functional and structural data suggest that sarbecoviruses have distinct receptor usage profiles, differences which can likely be attributed to variations within the RBD-ACE2 interaction interface, for example in the Δ4-loop. To understand whether patterns of generalism or specialism in the bat reservoir host are conserved in potential intermediate reservoirs we expanded our analysis to a wider range of mammalian ACE2 proteins.

### Bat sarbecoviruses also have broad tropism for mammalian ACE2

Based on previously established ACE2 restrictions [15,17,18,21,41], evidence for a role in spillover of sarbecoviruses into humans [42–44], and/or the identification of SARS-CoV-2 reverse zoonotic spillover [25–27,44,45] we selected 19 additional mammalian ACE2s for analysis including human, livestock/pets (dog, cat, pig), rodents (mouse, rat, Syrian hamster), potential intermediate reservoirs (red fox, civet, ferret, pangolin, mink, raccoon dog, white-tailed deer, red deer), and monkeys (marmoset, squirrel monkey, lemur, macaque) (S2 Table). Clade II Spikes that did not use any of the screened bat ACE2s in the initial screen were excluded from further analysis. Similar to the bat ACE2s, clade Ib Spike pseudotyped viruses exhibited broad tropism with the majority of mammalian ACE2s tested, with notable restrictions for SARS-CoV-2 with mouse, rat, ferret, mink, marmoset and squirrel monkey ACE2, as published previously [15]. Interestingly, these restrictions were less obvious for the closely related BANAL viruses, including BANAL-20-52 whose RBD is almost identical to SARS-CoV-2, whereas RaTG13 was much more restricted. The mammalian ACE2 usage profile of the clade III specialists RfGB02 and RhGB07, as well as the clade V specialist Rc-o319, were more divergent than with the bat ACE2s. For example, whilst RhGB07 Spike was able to use the ACE2 from many mammals, including pangolin and raccoon dog to enter cells, Rc-o319 was entirely restricted with this panel. The lack of an absolute correlation between generalism and specialism in bats and other mammals was also seen for clade Ia pseudoviruses, which demonstrated almost universal usage of all ACE2s in our mammalian ACE2 receptor usage, aside from New World monkeys (Fig 3A; S1 Data). Comparison of mammalian ACE2 protein sequences (relative to human ACE2) revealed a much higher sequence conservation than seen for bat ACE2s (Fig 1C), particularly at the RBD-binding interface, with divergence observed at amino acid residues 24, 34, 79 and 82, and to a lesser extent at positions 30, 31 and 353 (Fig 3B and 3C).

**Fig 3:**
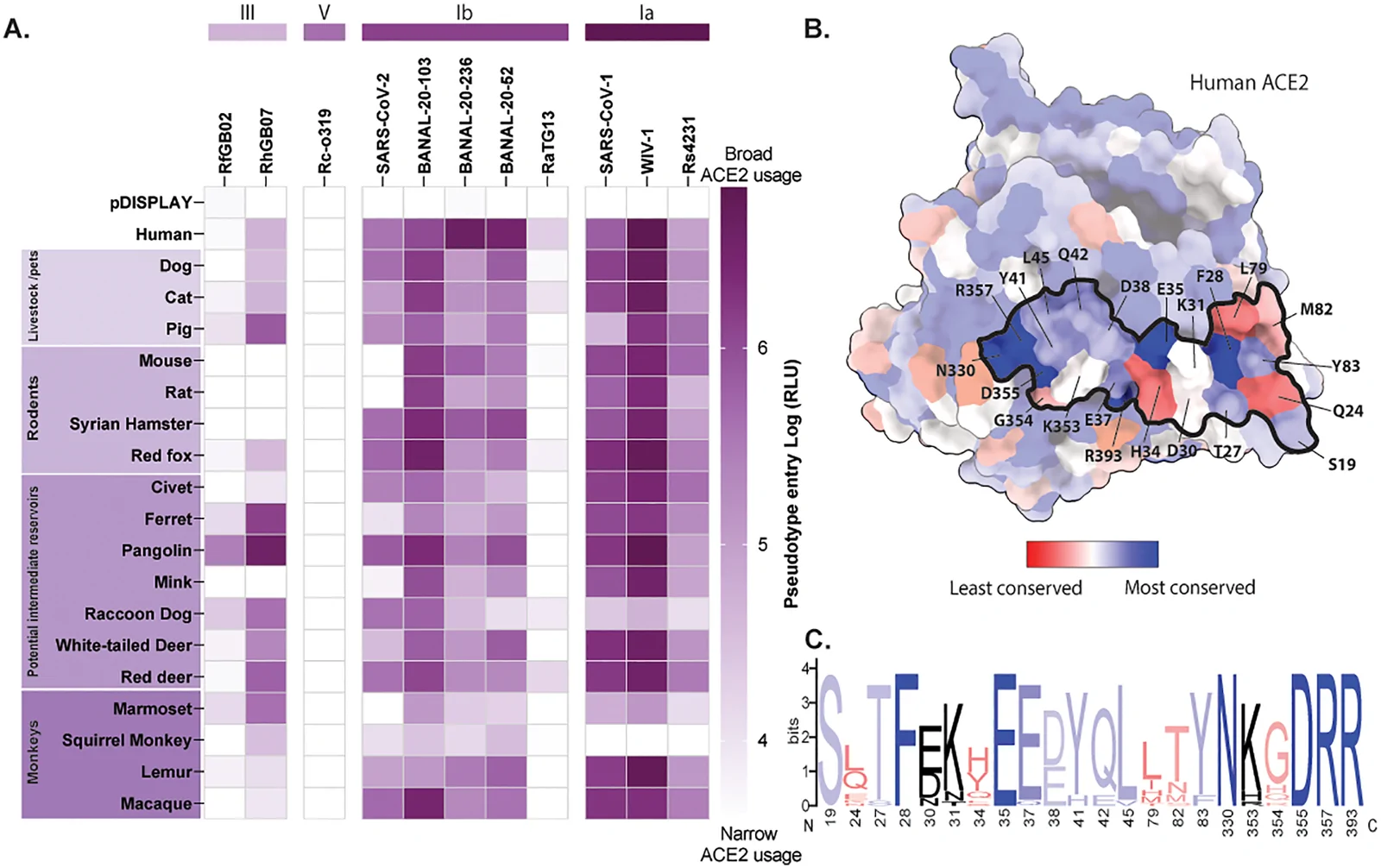
Bat sarbecovirus usage of mammalian ACE2. **(A)** Heatmap showing the receptor usage of bat sarbecovirus-pseudotyped viruses with mammalian ACE2s, including human, livestock/pets, rodents, potential intermediate hosts and monkeys. The mean log RLU from 3 separate experiments is shown and a pDISPLAY control to illustrate the background levels of entry. **(B)** Surface representation of human ACE2 coloured with mammalian ACE2 conservation (human ACE2: PDB 6M0J), with the outlined region denoting the RBD-interacting sites, with individual amino acids labelled. **(C)** WebLogo (University of California, Berkley, USA) plots summarising the amino acid divergence within the mammalian ACE2 sequences used in this screen. The single-letter amino acid code is used with the vertical height presenting the relative frequency of that amino acid at each position. The positions at which ACE2 interacts with SARS-CoV-2 RBD are shown, N-terminal to C-terminal, with different colours representing amino acid conservation between the mammalian ACE2s examined in this study. The data underlying this Figure can be found in S1 Data.

### Bat and mammalian ACE2 usage has changed in line with continued SARS-CoV-2 evolution in humans

The D614G-containing SARS-CoV-2 lineage, B.1 was responsible for the first wave of the COVID-19 pandemic, with subsequent waves caused by genetically divergent variants including Alpha, Beta, Delta, and Omicron. All these variants, in particular Omicron, were defined by the introduction of important mutations within the Spike protein (Figs 4A and S8A; S1 Data). To examine changes to bat and mammalian ACE2 receptor usage by these SARS-CoV-2 variants, we measured the entry efficiency of Spike-based pseudotypes in cells overexpressing ACE2 orthologues. For bat ACE2s, we observed enhanced (compared to B.1) bat ACE2 usage with the Alpha, Beta and Delta variants approaching or exceeding the levels recorded for human ACE2, however, the overall pattern of receptor usage did not change. In contrast, a divergent pattern was seen with Omicron BA.1, including shifted receptor usage of *Rh. cornutus, Rh. pusillus, Rh. ferrumequinum* and *L. lyra* ACE2 (Fig 4B, left panel; S1 Data).

**Fig 4:**
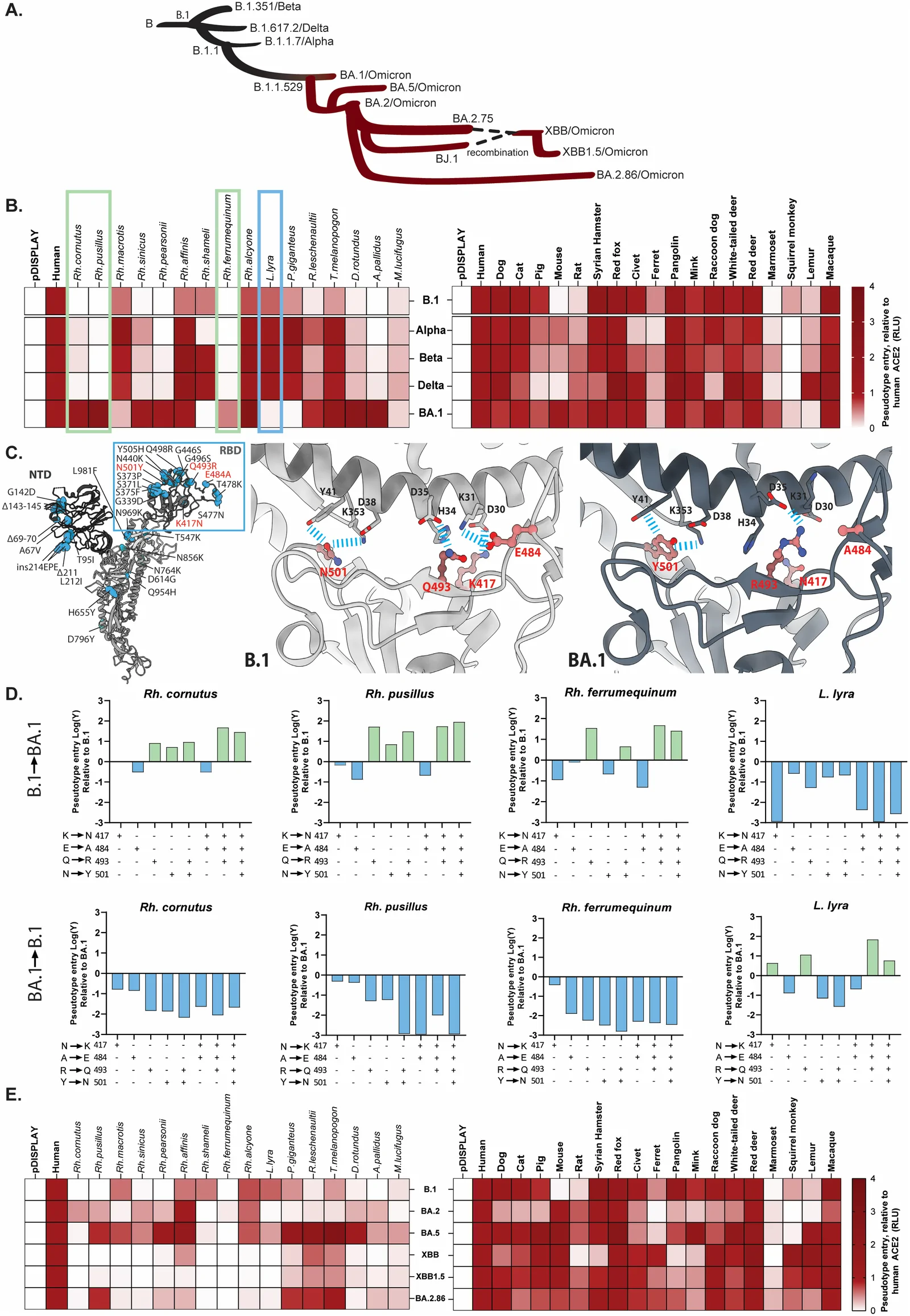
Species tropism of bat and mammalian ACE with SARS-CoV-2 variants. **(A)** Schematic of the emergence of SARS-CoV-2 variants depicting the divergence of the B lineage variants, including those variants of concern that grew to global dominance (B.1, Alpha, Beta, Delta, Omicron [black lines]), and the Omicron subvariants [red lines], some of which emerged via recombination events (dotted lines). Heatmap showing the bat and mammalian ACE2 usage of SARS-CoV-2 variants with **(B)** the B.1, Alpha, Beta, Delta and BA.1 variants, or **(E)** BA.2 and its subvariants BA.4/5, XBB, XBB1.5, BA.2.86. Data are shown as the mean fold change in pseudotype entry (RLU) of 3 separate experiments, relative to human ACE2. **(C)** Structure of SARS-CoV-2 BA.1 (PDB 7XO7) with mutations listed compared to B.1. Zoomed images of the Spike protein for B.1 (PDB 6M0J) and BA.1 (PDB 7WBP), highlighting key residues at positions 417, 484, 493 and 501 and the resultant interactions with human ACE2. **(D)** Specific amino acids within the B.1 RBD were mutagenised to make the RBD more BA.1-like (K417N, E484A, Q493R or N501Y) (top panel), or within BA.1 RBD to make the RBD more B.1-like (N417K, A484E, R493Q or Y501N) (bottom panel), either by themselves or in combination. Entry of these mutants was tested with *Rh. cornutus, Rh. pusillus*, *Rh. ferrumequinum*, and ***L.*** *Lyra* ACE2s. Data is shown as log fold-change in pseudotype entry, with green bars indicating an increase in virus entry and blue bars a decrease in entry, relative to B.1 (top panel) or BA.1 (bottom panel), highlighted also in Fig 4B. The data underlying this Figure can be found in S1 Data.

In contrast, the species tropism for a wider selection of mammalian ACE2s was somewhat unchanged, maintaining the generalist properties of B.1, with the same notable restrictions, e.g., New World monkey (marmoset and squirrel monkey) and ferret. Interestingly, an increase in mouse and rat ACE2 usage was detected in all variants harbouring N501Y mutations in the Spike RBD, as previously shown (Fig 4B, right panel; S1 Data) [21].

Focussing on the significant differences in bat ACE2 receptor usage between B.1 and Omicron BA.1, we next investigated which mutations in the Spike were responsible. Compared to B.1, Omicron BA.1 has a significant number of amino acid substitutions in Spike, with 13 mutations in the RBD alone, particularly at residues that interact directly with ACE2, with the 417N and 484A mutations reducing potential interactions (Fig 4C). Based on our structural understanding of RBD residues that interact directly with ACE2, and/or previous evidence of a direct contribution to altered species tropism, we generated B.1→BA.1 substitution mutants (K417N, E484A, Q493R, N501Y) either individually or in combination. In addition, inverse mutations (BA.1→B.1) were built into a BA.1 backbone, with all mutants being functional when titrated on cells stably expressing human ACE2 (S8B Fig; S1 Data).

We then assessed the entry phenotype of these mutated Spikes with bat ACE2s that showed a switch towards increased (*Rh. cornutus, Rh. pusillus, Rh*. *ferrumequinum*) or decreased receptor usage (*L. lyra)* with BA.1 compared to B.1. We noted that whilst the K417N and E484A mutants on their own, or in combination, had little impact on receptor tropism, the Q493R and N501Y mutations, either in isolation or in combination with other mutations, resulted in increased receptor usage of *Rh. cornutus* and *Rh. pusillus* ACE2. For the *Rh. ferrumequinum* ACE2, the Q493R mutation alone was sufficient to switch receptor usage. For *L. lyra*, the decrease in receptor usage was largely attributable to the change at position 417 (Fig 4D, top panel; S1 Data). When the corresponding mutations were introduced into the BA.1 backbone, a similar phenotype was observed, with amino acid changes at positions 493 (R493Q) and 501 (Y501N) being detrimental to *Rh. cornutus Rh. pusillus* and *Rh. ferrumequinum* ACE2 receptor usage, especially when in combination. Likewise, the 417 (N→K) and 493 (R→Q) mutations in BA.1, as single mutations or together, were able to revert the observed restriction between BA.1 Spike and *L. lyra* ACE2 usage (Fig 4D, bottom panel; S1 Data).

Finally, we also examined the receptor usage of contemporaneous Omicron sub-lineages that have emerged and risen to dominance since 2022. Interestingly, aside from BA.4/5, and to a lesser extent BA.2.86, many of the Omicron variants tested (BA.2, XBB and XBB1.5), exhibited a reduced or complete loss of receptor usage for many bat ACE2 receptors (Fig 4E, left panel; S1 Data). This was not evident for the wider library of mammalian ACE2 species, which remained widely used by the Omicron sub-linages tested (Fig 4E, right panel; S1 Data). Indeed, there was evidence of previous restrictions being overcome by variants such as XBB and BA.2.86, e.g., New World monkey and ferret ACE2s. In summary, the continued evolution of SARS-CoV-2 in humans has shifted the receptor usage pattern of this sarbecovirus away from an ancestral bat phenotype, towards one that is more generalistic for other mammals, including humans.

### Multiple bat sarbecoviruses share antigenic similarity to SARS-CoV-2

Several bat sarbecovirus Spikes in our study showed a broad host range for mammalian ACE2s, including human ACE2 (Fig 3A), suggesting the potential for spillover into human populations. To ascertain whether pre-existing immunity following infection with SARS-CoV-2 can provide cross-protection against related bat sarbecoviruses should they emerge in the human population, we examined their antigenic similarity by ELISA and VNT with a panel of COVID-19 convalescent plasma/serum obtained from the Medicines and Healthcare products Regulatory Agency (MHRA), UK. The antibody panel includes the World Health Organization (WHO) International Standards for anti-SARS-CoV-2 immunoglobulins, and the International Reference Panel material for antibodies to SARS-CoV-2 variants of concern B.1 (wildtype, WT), Alpha, Gamma, Delta, and Omicron BA.1. We also tested plasma from vaccinees with high and low neutralising antibody titres to SARS-CoV-2, as well as negative plasma collected from healthy donors pre-2019 (Fig 5A). By ELISA, we observed high levels of human IgG-based cross-specific binding to the RBDs of clade Ia and Ib viruses. Interestingly, we also noted binding to the RhGB07 and Rc-o319 RBD by most of the sera/plasma tested despite their phylogenetic divergence. (S9 Fig; S1 Data). The same sera/plasma also exhibited moderate-to-high neutralisation titres against equivalent pseudotyped viruses (BANAL-20-103, BANAL-20-236: ns; BANAL-20-52, * *p* < 0.05; Rs4231: ** *p* < 0.005; WIV-1: *** *p* < 0.0005, compared to SARS-CoV-2) (Fig 5B and 5C; S1 Data). Due to the inefficient usage of human ACE2 by Rc-o319 and RhGB07 pseudotypes, the target cells for these virus neutralisation assays were instead transiently transfected with *Rh. cornutus* ACE2 and *Rh. alcyone* ACE2, respectively. Compared to SARS-CoV-2 we noted lower neutralisation titres against RhGB07 (***, *p* < 0.0005), and lower still against Rc-o319 (***, *p* < 0.0005) (Fig 5B and 5C; S1 Data). Interestingly, the binding and neutralisation phenotype observed for these two viruses, which were the most antigenically distant in our screen, were similar to those observed for Omicron (BA.1). This is perhaps unsurprising given that most of the sera/plasma tested was taken before BA.1’s emergence; however, it does highlight the role of immune pressure in driving SARS-CoV-2 evolution (S8A–S5C Fig; S1 Data). Another interesting observation was that in some ELISAs we did not detect binding, e.g., S2-03 did not bind to the RhGB07 RBD, and, 21/296 and S2-04 did not bind to WIV-1 RBD, however, we were still able to detect neutralisation of respective pseudotypes, suggesting neutralisation sites outside the RBD (Figs 5B and S8; S1 Data). In summary, both polyclonal sera and plasma from SARS-CoV-2 convalescent individuals shows robust evidence for the presence of cross-neutralising pan-sarbecovirus antibodies.

**Fig 5:**
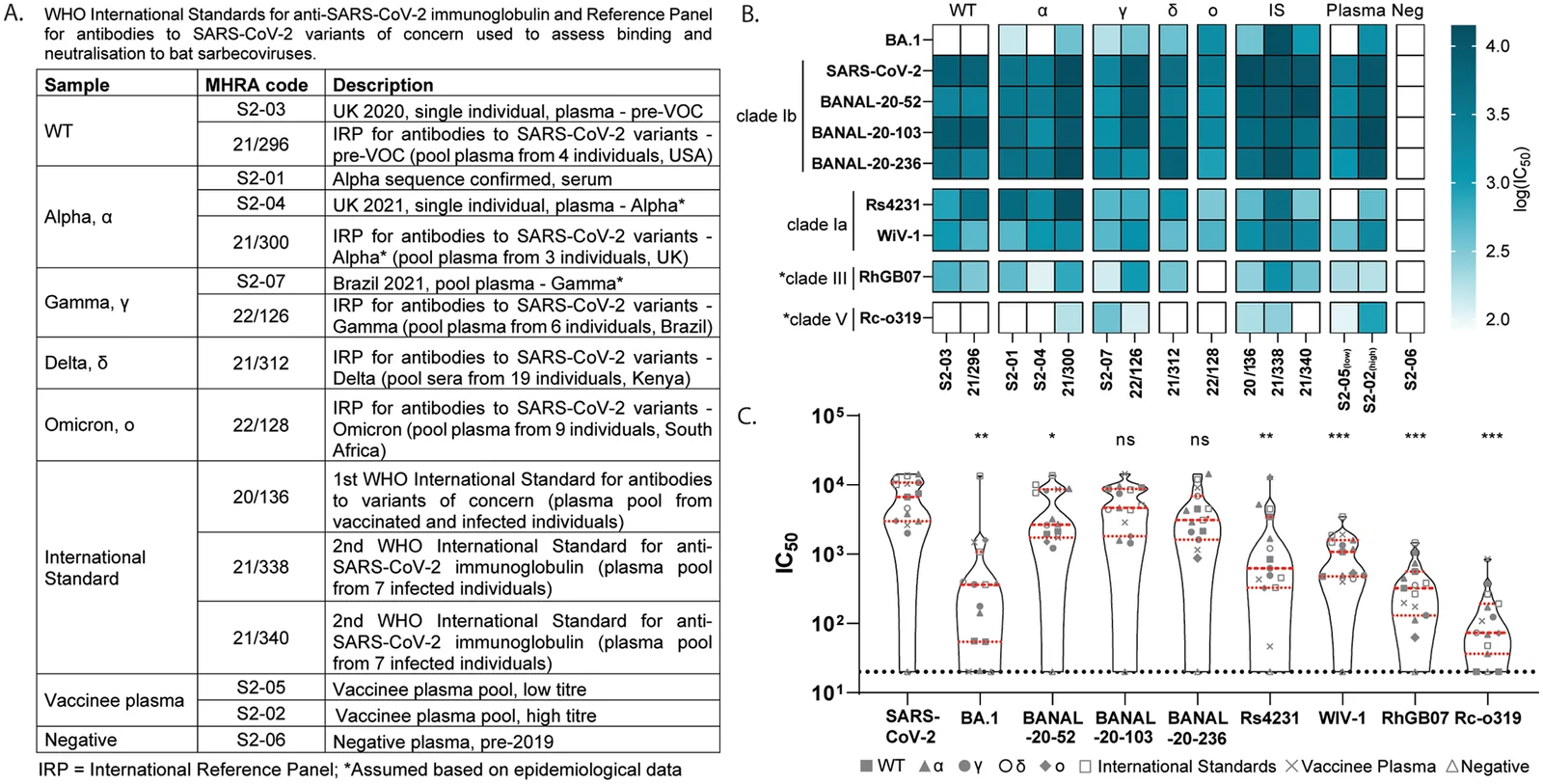
Antigenicity of bat sarbecoviruses using SARS-CoV-2 polyclonal convalescent sera. **(A)** Table outlining the different convalescent plasma/serum used to investigate the antigenicity of bat sarbecoviruses. These include samples from individuals following infection with the wildtype (WT), Alpha, Gamma, Delta, or Omicron BA.1 variants, either confirmed by PCR or assumed based on epidemiological data. The sample set also includes WHO International Standards provided by MRHA (NIBSC), pooled vaccinee plasma (low and high titre) and negative plasma, pooled from individuals pre-2019. Information about the COVID-19 standards and reagents can be found here: WHO/BS/2022.2427: Establishment of the 2nd WHO International Standard for anti-SARS-CoV-2 immunoglobulin and Reference Panel for antibodies to SARS-CoV-2 variants of concern. **(B)** Heatmap showing the log (IC_50_) values of neutralisation (the dilution at which there was a 50% reduction in luciferase activity) of SARS-CoV-2, BANAL-20-52, BANAL-20-103, BANAL-20-236, Rs4231, WIV-1, RhGB07, Rc-o319 and BA.1 viruses in the presence of the MHRA (NIBSC) reference reagents. HEK293T cells stably expressing human ACE2 were used for all neutralisation assays, apart from RhGB07 and Rc-o319, where HEK293T cells overexpressing *Rh. alcyone* or *Rh. cornutus* were used, respectively (indicated by an asterisk). **(C)** Violin plots show collated IC_50_ values for the neutralisation of these viruses with each sera set, with the median, upper and lower quartiles (red lines) and limit of detection (black dotted line) shown. A one-way ANOVA with Dunnet’s multiple comparison was performed, comparing to SARS-CoV-2, with significance values indicated (* = *p* < 0.05, ** = *p* < 0.005, *** = *p* < 0.0005, ns = non-significant). The data underlying this Figure can be found in S1 Data.

### Pan-sarbecovirus monoclonal antibodies are identifiable following breakthrough SARS-CoV-2 infection

Building on this cross-neutralisation data, we confirmed the RBD as the predominant target for this immune recognition. Using SARS-CoV-2 as an exemplar ACE2 generalist and Rs4231 as a specialist (Figs 1D, 3A, and 5), we generated RBD chimeras of these two Spikes and confirmed their functionality (S10A Fig; S1 Data). We opted against the more phylogenetically and phenotypically distant viruses, RhGB07 and Rc-o319, because of their inefficient human ACE2 usage, which would limit direct comparison with SARS-CoV-2. Using a panel of RBD-specific monoclonal antibodies (mAbs) isolated from SARS-CoV-2 breakthrough infections [46], we then conducted neutralisation assays with these chimeras. We detected a reduction in IC_50_ when the Rs4231 RBD was introduced into the SARS-CoV-2 backbone (*p* < 0.0001), whereas the converse was observed when the SARS-CoV-2 RBD was inserted into the Rs4231 backbone (*p* < 0.001) (Fig 6A; S1 Data). We also confirmed that the differences in ACE2 receptor usage tropism between these two Spikes mapped to the RBD (S10B Fig; S1 Data), e.g., switching mouse and marmoset ACE2 usage (S10B Fig; S1 Data). These data confirmed the RBD as a major target for cross-specific antibodies, and that both receptor generalism/specialism and antigenicity functionally partition to the same Spike protein sub-domain.

**Fig 6:**
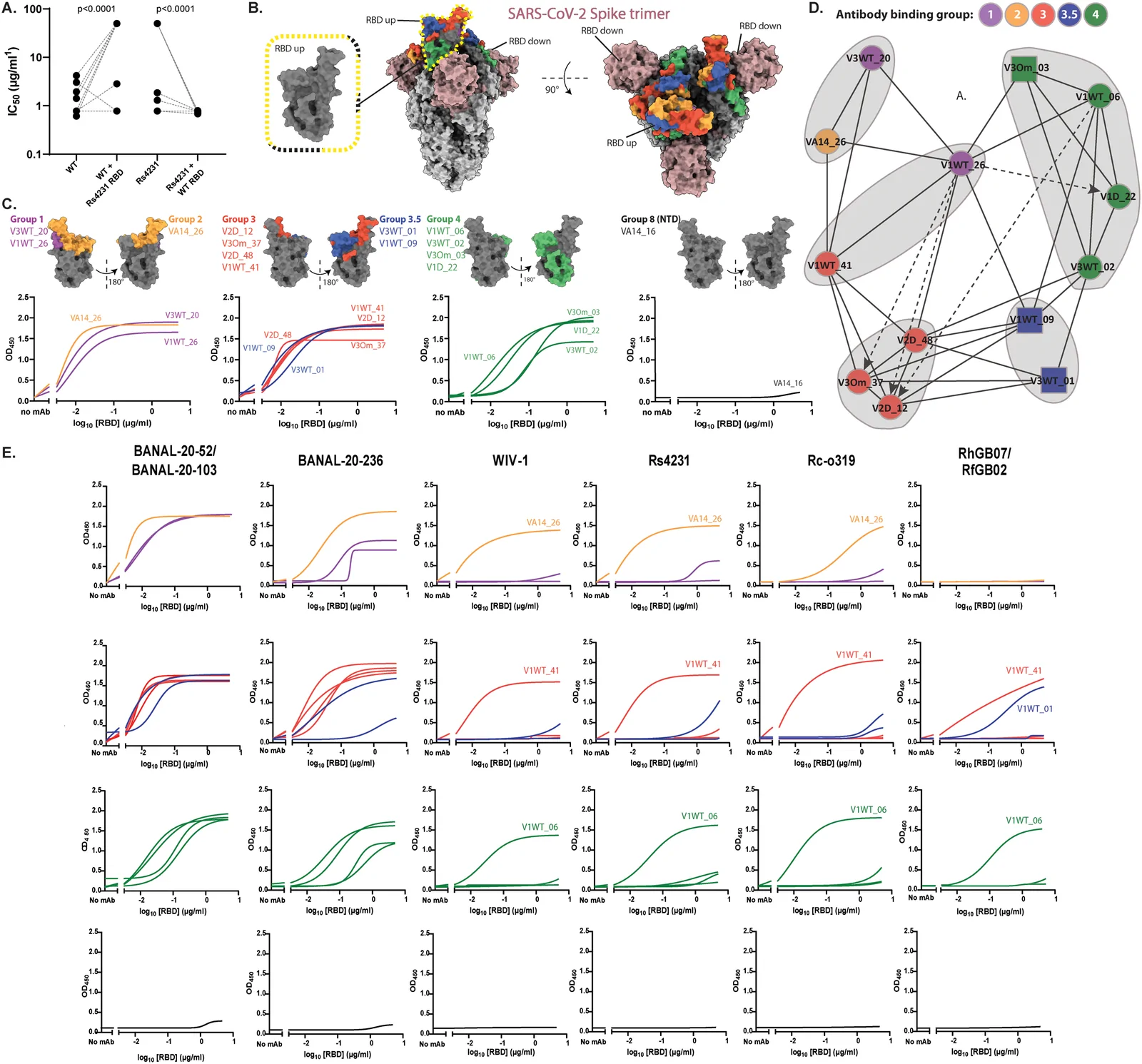
Binding of monoclonal antibodies isolated from SARS-CoV-2 breakthrough infections to bat sarbecovirus RBDs. **(A)** Neutralisation of SARS-CoV-2 and Rs4231 RBD chimeras with SARS-CoV-2 RBD-specific monoclonal antibodies (mAb) (IC_50_ µg/ml), with dotted lines linking matched samples. The *p* values are shown, calculated by comparing the change in mean IC_50_ value and by using Tukey’s multiple comparison tests, respectively**. (B)** Spike structure of SARS-CoV-2 with 2 RBD-down and 1 RBD-up (PDB 7QTI) with the binding epitope regions of the different mAb competition groups is shown with both a side- and top-view (Group 1 = purple, Group 2 = orange, Group 3 = red, Group 3.5 = blue, Group 4 = green, Group 8 = non-RBD, NTD-specific, defined previously [46–48]. **(C)** SARS-CoV-2 RBD structures (PDB 7QTI) highlighting a broad epitope footprint of the mAb competition groups corresponding binding data. Each of the plots shows a single mAb binding in an ELISA using purified **(C)** SARS-CoV-2 or **(E)** other bat sarbecovirus RBDs. Plots show raw optical density (OD) values with curves calculated using a least-squares regression non-linear fit. **(D)** Epitope binning by surface plasmon resonance (SPR) identifies 5 epitope communities broadly aligning with the antibody competition groups. Grey circles indicate communities by setting the associated dendrogram threshold to 0.45. Solid lines indicate symmetrical blocking, regardless of which mAb is the ligand. Dotted lines indicate asymmetrical blocking, with the arrowhead indicating the direction of blocking, pointing towards the antibody immobilised as a ligand. Circles indicate antibodies for which ligand and analyte data was available, squares indicate only analyte data was available. The data underlying this Figure can be found in S1 Data.

The mAbs used in our screen have been characterised in more detail previously, based on binding and competition assays with mAbs to SARS-CoV-2 RBD (Group 1 [purple], Group 2 [orange], Group 3 [red], Group 3.5 [blue], Group 4 [green] and Group 8 [non-RBD binder]) [46] (Fig 6B). To confirm these mAb groups and to provide additional resolution, binding ELISAs and epitope binning competition assays were performed by assessing binding to SARS-CoV-2 RBD. In initial ELISAs, all RBD-specific mAbs bound to the SARS-CoV-2 RBD, whereas the NTD-specific mAb VA14_16 failed to recognise this protein, as expected (Fig 6C; S1 Data). Epitope binning was then performed by high-throughput surface plasmon resonance (SPR) on the Carterra platform. In general, the same antibody binding groups were preserved, with additional blocking interactions between groups observed (Figs 6D and S11). Blocking interactions between group 3, 3.5, and 4 antibodies varied, indicating the presence of subtle differences in antibody binding within these groups (Fig 6D). For example, antibodies VA14_26 (group 2) and V1WT_41 (group 3) were characterised as having similar blocking phenotypes, indicative of epitope overlap (Fig 6D). Interestingly, the V1WT_26 antibody (group 1) showed blockage of group 2, 3, and 4 antibodies, despite group 1 mAbs occupying binding a distinct region of the RBD [46], suggesting possible steric hindrance (Fig 6D).

Building on the partial neutralisation of Rs4231 with these mAbs, we performed a more comprehensive assessment of their binding (by ELISA) phenotype, against other bat sarbecoviruses in our library. As with the polyclonal sera tested above, SARS-CoV-2, BANAL-20-52 and BANAL-20-236 RBDs were bound well by all the mAbs tested. In contrast, the more divergent WIV-1, Rs4231, Rc-o319 and RhGB07 RBDs were bound less robustly (Fig 6E; S1 Data). However, significant binding was maintained by antibodies in group 2 (VA14_26, except RhGB07), group 3 (V1WT_41) and group 4 (V1WT_06) (Fig 6C; S1 Data). The lack of binding of VA14_26 with RhGB07 RBD is likely due to differences in amino acid residues present at the antibody-RBD interface that result in weaker, repulsive interactions (K370E and R400K in RhGB07 compared to SARS-CoV-2, WIV-1 and Rc-o319) (S12A Fig). Interestingly, these 3 mAbs were also able to bind the RBD of the non-ACE2-using clade II virus, RacCS203, albeit to a lesser degree (S12B Fig; S1 Data). As expected, none of the group 8 mAbs (NTD-specific) bound to any RBD (Fig 6E; S1 Data).

A representative subset of these sarbecovirus RBDs and mAbs were next analysed for complementary binding kinetics and affinity by ELISA and SPR, respectively. Generally, there was good correlation between the assays, wherein mAbs with low EC_50_ values also exhibited lower *K*_D_ (dissociation constant) values, indicated higher affinity (Fig 7A; S1 Data). However, some RBD/mAb interactions characterised as non-binding by ELISA showed low binding by SPR and a greatly increased *K*_D_ (Figs 7A and S12C; S4 Table; S1 Data). For example, V3Om_03 showed minimal to no binding to the more divergent RBDs in ELISA, but in SPR a low level of binding was detected for Rs4231 and Rc-o319 (Fig 7A; S4 Table; S1 Data). Interestingly, the converse trend was observed for mAb V3WT_20 with RhGB07 RBD, with binding identified by ELISA but not by SPR (Fig 7A; S4 Table; S1 Data).

**Fig 7:**
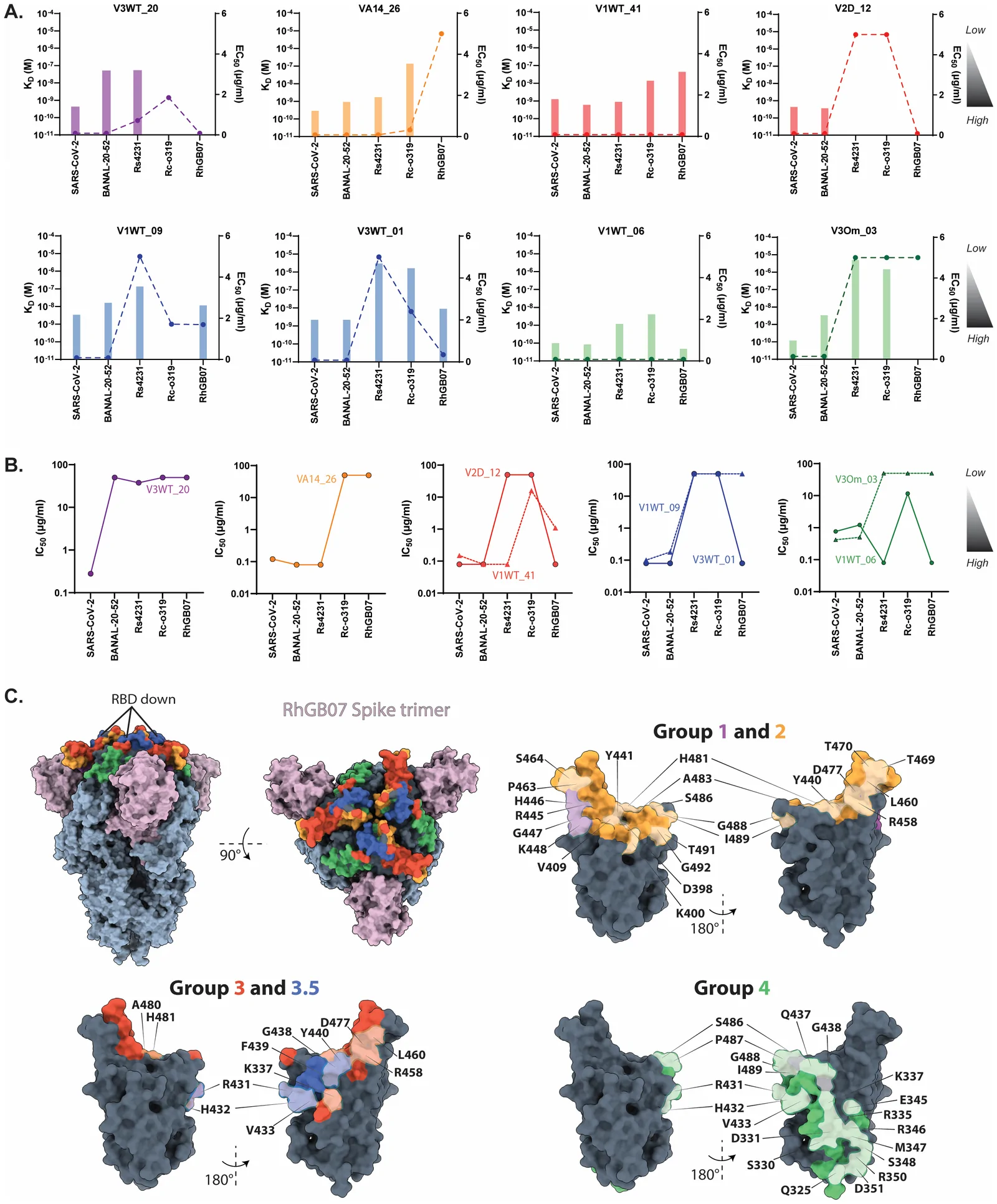
Antigenicity of bat sarbecoviruses using monoclonal antibodies isolated from SARS-CoV-2 breakthrough infections. **(A)** Dissociation constant (K_D_ **(M)**, 2−4 replicates + sd) of mAb binding kinetics calculated by SPR (bars) and ELISA EC_50_ (µg/ml, 2 replicates + sd) values (broken lines with filled symbols) for a subset of mAbs and sarbecovirus RBDs. For K_D_, no bar indicates no binding by SPR. For EC_50_, lower limit of detection: <0.08 µg/ml, upper limit of detection: >5 µg/ml. **(B)** Neutralisation (IC_50_) titres shown for each mAb against bat sarbecovirus pseudotyped viruses (µg/ml), with limits of detection indicated (lower limit: <0.08 µg/ml; upper limit: >50 µg/ml for IC_50_). HEK293T cells stably expressing human ACE2 were used for all neutralisation assays. apart from RhGB07 and Rc-o319, where HEK293T cells overexpressing *Rh. alcyone* or *Rh. cornutus* were used, respectively. Scale bars (right) represent direction of increased binding or neutralisation. **(C)** RhGB07 trimer and RBD, with antibody competition groups highlighted, and are coloured with the same colouring as the SARS-CoV-2 trimer in (Fig 6B). Regions of divergence in binding epitopes in RhGB07 compared to SARS-CoV-2 are depicted, with amino acid residues denotes. Colours in all graphs/structures indicate mAb binding competition groups (Group 1 = purple, Group 2 = orange, Group 3 = red, Group 3.5 = blue, Group 4 = green). EC_50_, K_D_ and IC_50_ values be found in S4 Table. The data underlying this Figure can be found in S1 Data.

Finally, we examined the neutralisation of various pseudotyped spikes by these mAbs. These experiments showed a similar trend, with BANAL-20-52 exhibiting binding EC_50_s and neutralisation IC_50_ values of <0.08 µg/ml, close to SARS-CoV-2 (Fig 7B; S4 Table; S1 Data). Mirroring the ELISA data, Rs4231 (clade Ia), RhGB07 (clade III) and Rc-o319 (clade V) were less well neutralised; however, the same three monoclonal antibodies (VA14_26, V1WT_41 and V1WT_06) showed good efficacy, highlighting competition groups 3, 3.5 and 4 antibodies as being important mediators of cross-neutralisation (Fig 7B; S4 Table; S1 Data). Neutralisation IC_50_ titres also correlated with K_D_, with a larger *K*_D_ correlating with weakened or abrogated neutralisation. In contrast, the three antibodies which exhibited broad RBD binding and neutralisation (VA14_26, V1WT_41, and V1WT_06) displayed a much narrower fluctuation in *K*_D_ consistent with their ability to bind all the RBDs tested (Figs 7A, 7B, and S12C; S4 Table; S1 Data). Mapping the broad footprints of where these mAb competition groups might bind onto the Spike trimer of RhGB07 highlighted a significant number of amino acid residues that differ to SARS-CoV-2 RBD (Fig 7C). RhGB07 has a 3 RBD-down, closed conformation, which may contribute to mAb accessibility, particularly the Group, 3.5 and 4 mAbs, which target the most exposed epitopes on in the RhGB07 RBD down/closed state (Fig 7C). Our companion article describes in more detail the structural basis for the broad specificity of these antibodies to sarbecovirus RBDs [49].

## Discussion

Identifying differences in receptor usage between closely related viruses and the key amino acid substitutions that underpin restriction are important tools for understanding the mechanisms of spillover. However, performing this work in a wholistic way, i.e., one that develops broad, translatable knowledge that improves our understanding of general rules governing spillover is more likely to have tangible benefits on pandemic prevention and preparedness. Herein, we describe that across the sarbecovirus sub-genus we could only find broad generalist ACE2-using viruses within the same clade (clade I) as SARS-CoV and SARS-CoV-2, and, that these viruses appear to all be antigenically similar. Interestingly, even for the more specialised ACE2-using sarbecoviruses, we were still able to detect low levels of cross-neutralisation, which we could attribute to specific antibody classes. These data, combined with high post-pandemic levels of COVID-19 immunity in human populations, may mean the risk of future sarbecovirus zoonotic spillovers, at least for the ones described in this study, is diminished. However, additional surveillance and continued risk assessment are required for other sarbecoviruses, especially “unknowns”, with these studies providing additional information for understanding spillover potential.

To arrive at this conclusion, we first assessed the pattern of generalism and specialism across the various extant clades of sarbecoviruses. Like SARS-CoV-2, other clade Ib viruses were mostly bat ACE2 generalists. In contrast, clade Ia and to a greater extent, clades III and V were more specialised, using primarily the ACE2 receptor of the bat species from which the virus was isolated. Interestingly, within clade Ib, RaTG13 showed a distinct, more restricted pattern ACE2 usage, despite sharing 98% amino acid similarity with SARS-CoV-2 and clustering in the same clade as SARS-CoV-2 and the BANAL viruses. This is likely due to amino acid substitutions at key ACE2-binding sites, e.g., the F486L substitution in RaTG13 weakens hydrophobic interactions with side chain residues L79, M82 and Y83 in ACE2 [15]. Similarly, the clade Ib sarbecovirus RShSTT200 has also been shown to have restricted host range, including to human ACE2, which the authors attributed to amino acid differences in Spike, relative to SARS-CoV-2, e.g., P486F, T346R, D487N, Y496G, or deletions at positions 445–445 and 448–489 (SARS-CoV-2 numbering) [50]. Importantly, this implies that generalism and specialism cannot entirely be predicted by phylogenetic relationship and, indeed, a limitation of our study is that we did not look at more viruses to understand this further. Consistent with other studies, the clade II viruses did not use ACE2 as a functional receptor, despite robust Spike protein expression. This can largely be attributed to two deletions in the RBD spanning amino acids 445–449 (region 1) and 473–488 (region 2), relative to SARS-CoV-2, which have been described elsewhere as being indicative of ACE2 tropism [51]. However, substitutions of these regions, or the entire receptor-binding motif with SARS-CoV-2, failed to restore ACE2 binding or entry [17,51], so these are not the only restrictions to ACE2 usage. The clade III viruses RhGB07 and RfGB02 had restricted bat and slightly broader mammalian ACE2 usage, with RhGB07 being able to utilise hACE2, as previously described [10], with RhGB07 RBD exhibiting lower binding by ELISA to *Rh. alycone, M. lucifugus* and human ACE2, compared to SARS-CoV-2 RBD (S5D Fig). Quantifying K_D_ values of these binding interactions by SPR or by bio-layer interferometry would elucidate the affinity of RhGB07 RBD with these ACE2s, to better understand the interplay of affinity with receptor entry. Interestingly, although the RBD sequences of RfGB02 and RhGB07 are identical at the RBD/ACE2 binding interface, the former could not use hACE2 - indicating again sequences outside the direct ACE2/RBD interface can influence ACE2 usage [10]. Our structural data for RhGB07 identified that the RhGB07 deletion at position 449 (region 1) potentially disrupts electrostatic interactions with residues 38 and 42 of ACE2, which have also been shown to be important for bat ACE2 receptor tropism [19], which may explain the specialist receptor tropism of this virus. Similar results have been seen for a related clade III virus, PRD-0038, which branches separately from RhGB07 and RfGB02 [14]. Although this virus also uses hACE2 poorly, its host range extends to geographically relevant bat species, *Rh. alcyone*, *Rh. landeri* and specific alleles of *Rh. ferrumequinum* ACE2. One or two amino acid substitutions in Spike—T487W and K482Y—were shown to be sufficient to increase tropism for these bat ACE2s [14]. Conversely, Khosta-2 (another clade III virus) was shown to be able to bind human ACE2 in structural models and infect human cells in vitro using full-length Spike, when in the presence of trypsin [52,53]. In our study, deletion of the 446–449 region in SARS-CoV-2 and insertion of these residues in RhGB07 indeed showed an increase in entry and binding with human (Fig 2C and 2D). However, RhGB07 mutants generated to mimic other bat sarbecoviruses only resulted in an increase in bat ACE2 tropism for the broad generalist viruses (RhGB07 437_438insVGGNY, as in SARS-CoV-2) (Fig 2B), again highlighting the wider importance of other regions in the RBD and/or Spike in restricted ACE2 usage. Kosugi and colleagues, showed a similar result whereby ‘filling in’ the deletion in the clade Ib Spike, RShTT200 did not confer human ACE2 usage, whereas combinatorial substitutions in other regions of Spike was more successful [50]. Lastly, the clade V Spike Rc-o319 exhibited similarly broad restrictions in bat ACE2 usage and was unable to use human or other mammalian ACE2s. Other clade V sarbecoviruses Rc-os20, Rc-kw8 and Rc-mk2 have displayed similar restrictions, using primarily the cognate *Rh. cornutus* ACE2 receptor [50]. The Rc-o319 RBD contains a 10 amino-acid deletion in region 2 (481–499) compared to the SARS-CoV-2 RBD, which likely contributes to this specialist receptor usage profile [51]. This has been confirmed by recent structural data which highlights structural incompatibility between the Rc-o319 RBD and human ACE2, which require combinatorial mutations in specific RBM regions to gain medium-to-high affinity for human ACE2 [54]. Of note, viruses from clade IV were not included due to the unavailability of complete sequences at the time of commencing our study. Indeed, one limitation of our approach is that sequences were selected based on data from similar studies [10,14,18,36,52,55], however, this led to overrepresentation of certain clades. In the future, we could use more unbiased criterion to select sequences, as we recently described for the alphacoronaviruses, where Spike libraries were constructed algorithmically to maintain maximum genetic diversity [56]. Nevertheless, other studies have since investigated the tropism of a clade IV virus, RaTG15, which shares 72.6% amino acid identity with SARS-CoV-2 RBD. The RaTG15 RBD bound to *Rh. affinis* and Malayan pangolin ACE2, and full-length Spike pseudoviruses were able to use these receptors for entry, however, no obvious binding or entry with hACE2 was detected [36]. A short deletion present in RaTG15 at amino acid positions 444–447, as well as variation at key ACE2 receptor-binding sites (486, 493, 494, 501) is likely to explain this specialism. Generally speaking, variation at the ACE2-binding interface likely impacts the affinity of different bat sarbecovirus Spikes for ACE2, as suggested in recent studies by Wang and colleagues and Kosugi and colleagues, which describe four regions in the Spike RBM that form contacts with ACE2, resulting in high affinity binding if 3–4 of these interfaces are intact, weak-to-moderate binding if 2 interfaces are intact, and little-to-no binding if only 1 interface is present [40,50]. This was shown for the clade Ib Spike RShSTT200 [50], the clade III Spikes BtKY72 and BM48-31 [40], and the clade V Spike Rc-o319 [54]. The optimal binding interfaces may also differ between clades, for example, the clade III RBD BtKY72 is able to bind *Rh. affinis* ACE2 with high affinity due to the presence of 3 of these binding interfaces, despite the deletion at positions 445–449 (SARS-CoV-2 numbering) [40]; conversely, for the clade Ib virus RShSTT200, which also contains the 445–449 deletion, interactions with the other three interfaces still result in weak binding with *Rh. affinis* ACE2 [50].

Although this study focussed on bat sarbecoviruses, related viruses have now been isolated from *Paguma larvata* (palm civet, e.g., SARS CoV SZ3 in China) and *Manius javanica* (pangolin, e.g., BetaCoV/P4L in China), respectively [18]. These hosts have been proposed as potential intermediate reservoirs for SARS-CoV-2, along with the raccoon dog [57], so future host range analyses should include these sarbecoviruses to understand the breadth of species tropism of possible virus intermediates. That being said, the larger unanswered question that remains is to understand what are the environmental selection pressures that maintain receptor generalism for some sarbecoviruses but not others. It is attractive to postulate that the overlapping host range of multiple bat species in Southeast Asia leads to significant selection pressure for generalism in resident sarbecoviruses (mostly clade 1), ecological pressures that are less prevalent in regions where specialists were isolated. However, the identification of specialists within clade 1b like RaTG13 is indicative of a more complicated and perhaps stochastic pattern.

Another important consideration is to understand the impact of the extensive, ongoing human adaptation of SARS-CoV-2 on its ‘reverse zoonosis’ potential. The oscillating pattern of bat ACE2 usage by SARS-CoV-2 variants was an interesting observation, especially the eventual drift away from bat tropism by later Omicron variants. We identified the Q493R mutation in Omicron BA.1, either by itself or in combination with other RBD-specific mutations (N501Y, E484A and K417N) as being an important determinant of this trend. Q493R has previously been shown to be important in overcoming bat ACE2 restrictions, due to an increased affinity for ACE2 residues 35 and 31, which were described as key determinants for bat ACE2 usage [58], along with ACE2 amino acid positions 27 and 42 [19,41]. Interestingly, in the BA.5 variant position 493 is reverted (R493Q), and we saw a corresponding loss of *Rh. cornutus* and *Rh. ferrumequinum* ACE2 usage here. Other notable animal ACE2 restrictions observed are for the platyrrhines ACE2s (marmoset and squirrel monkey), which exhibit lower levels of entry compared to catarrhines (lemur and macaque). This is attributable to ACE2 variation at positions 41 and 42 and the loss of important hydrogen bonds (which help binding to positions 446 and 500 in Spike) [59]. Despite shifts in bat ACE2 usage most SARS-CoV-2 variants have maintained their generalist receptor usage with a wider selection of mammal ACE2s. The impact of this generalism is now more widely appreciated than in 2020, as evidenced by white-tailed deer infections in the USA [25,27], mink in Denmark [44], and hamsters in China [45].

The second objective of our study was to relate this generalism and specialism to antigenicity. For example, the broad host range we observed for many clade I sarbecoviruses raises concerns that these viruses might spillover into human populations. Accordingly, we investigated the breadth of protection provided by COVID-19-derived immunity. For clade I viruses, we observed broad evidence for cross-neutralisation by sera from convalescent individuals, regardless of the infecting variant. Indeed immunisation of human ACE2-transgenic hamsters with a trivalent SARS-CoV-2/SHC014/XBB Spike nanoparticle vaccine also exhibited complete protection against challenge with other clade I viruses, WIV-1 and SHC014 [60], supporting our data demonstrating pan-clade I neutralisation from clade I-derived convalescent sera. We also noted some evidence of neutralisation against more specialised ACE2 users, e.g., RhGB07 and Rc-o319, with patterns of neutralisation that mirrored those observed for the antigenically distinct SARS-CoV-2 variant BA.1. However, the overall affinity and avidity of Spike for human ACE2 is worth considering and understanding, in relation to these more distantly related viruses. For example, a study examining the receptor tropism of another clade III virus—Khosta-2—exhibited binding of the RBD of this virus with human ACE2, but viral pseudotypes of expressing the Khosta-2 RBD were not neutralised by SARS-CoV-2-derived mAbs or vaccine sera [53]. Furthermore, we previously showed that RaTG13 Spike-based pseudotypes were neutralised more efficiently than SARS-CoV-2, using sera from COVID-19 vaccinated healthcare workers (with or without evidence of prior infection) [16]. A later cryoEM structure of the RaTG13 Spike revealed a more stable, closed conformation for the trimeric protein with low relative affinity for hACE2, compared to SARS-CoV-2 [61,62]. Our structural data for the RhGB07 trimer, which was also only found in a three-RBD down closed confirmation, is consistent with a similar pattern. The low overall affinity for human ACE2 of these bat virus RBDs, unlike BA.1, for example, could mean that even low-affinity nAbs are able to displace the RBD from human ACE2. In this context, extending our analysis to include sera from vaccinated individuals in the UK would be of interest to understand whether vaccine-derived immunity can enhance binding and neutralisation of bat sarbecoviruses. Nevertheless, to understand the cross-neutralisation we observed with convalescent sera in more detail, we instead expanded our study to include monoclonal antibodies derived from SARS-CoV-2 breakthrough infections. We identified three high-affinity antibodies, which belonged to three different RBD competition groups that were able to bind all sarbecovirus RBDs in our screen (apart from RhGB07), including the non-ACE2-using RacCS203. These mAbs were previously classified by competition ELISAs, using mAbs that bind known epitopes in Spike [46–48] and further characterised in our study by SPR. In our companion article, we determine the molecular mechanisms by which these three mAbs retain broad and potent binding. These data provide promising insights into the regions of Spike that could be targeted to design pan-sarbecovirus immunogens, or indeed the potential for these monoclonals to be used as pan-sarbecovirus therapeutics, informed by the conserved epitopes identified herein.

As discussed above, our findings provide promising insights into the role COVID-immunity may play in preventing future sarbecovirus spillover. However, an outstanding question that remains from our research is, ‘are there human ACE2-using configurations for coronavirus Spike RBDs that are entirely antigenically distinct from SARS-CoV-2?’ In simpler terms, is there another human-ACE2-using serotype of sarbecoviruses in the wild? One hypothesis, favourable from a public health perspective, is that any ‘specialist’ sarbecovirus that evolves to use human ACE2 (a ‘generalist’) will become antigenically recognisable by the population’s COVID-primed immune response. This would set the bar for spillover higher than anticipated; however, the emergence of very long branch SARS-CoV-2 variants, like BA.1 and BA.2.86, clearly highlights a great deal of plasticity in the RBD in binding human ACE2 very efficiently, while still navigating significant immune selection pressure. In addition, viruses like RhGB07—specialists that can still partially use hACE2—provide clear evidence that hACE2 usage can arises relatively early on the spectrum of specialism to generalism.

## Supporting information

S1 Fig Geographic range of bat species investigated in this study. The geographic location in which the bat sarbecoviruses analysed in this study have been isolated is coloured in blue on the world map, with separate maps depicting the range the bat species investigated in this study span. *Rhinolophidae* species have been grouped together and span across Europe, Asia and some parts of southern Africa. The *T. melanopogon, P. giganeteus, L. Lyra* and *R. leschenautii* species all span across southeast Asia, in particular overlapping in regions of India and parts of China. *The M.lucifugus, D. rotundus* and *A.pallidus* bat species can be found nesting in the Americas.
(TIF)

S2 Fig Pseudotype virus titrations and protein expression of ACE2s and sarbecovirus Spikes. **(A)** Lentiviral pseudotypes expressing different bat sarbecovirus Spikes were titrated on BHK-21 cells overexpressing human ACE2 (SARS-CoV-2, BANAL-20–52, BANAL-20–103, BANAL-20–236, RaTG13, SARS-CoV-1, WIV-1, Rs4231, Rf1, Rp3, RacCS203, RpYN06), *Rh. cornutus* ACE2 (Rc-o319) or *Rh. alcyone* ACE2 (RhGB07, RfGB02). Virus titres were used to normalise input virus for subsequent assays. **(B)** Expression of tagged ACE2 proteins was carried out in lysates from BHK-21 cells transfected with ACE2 constructs and probed for HA expression by Western blotting. **(C)** Sarbecovirus Spikes were used to generate lentivral-based pseudotypes, and the viral supernatant purified by ultracentrifugation under a 20% sucrose cushion. Purified viruses were probed with an anti-FLAG antibody (except RhGB07 and RfGB02, which are untagged), an anti-Spike antibody (S, full-length Spike; S1/S2, cleaved variant) and the lentivirus internal control, anti-p24 antibody. The data underlying this Figure can be found in S1 Data and S1 Raw Images.
(TIF)

S3 Fig Entry of bat sarbecoviruses by cell-cell fusion. **(A)** Heatmap showing virus entry by cell-cell fusion. HEK293T cells stably expressing half of a split GFP-Renilla (RLuc-GFP 1−7) luciferase reporter were transfected with bat sarbecovirus Spikes, and co-cultured with BHK-21 cells expressing the other half of the split reporter (RLuc-GFP 8−11), transfected with bat ACE2s. Productive cell-cell fusion results in reconstitution of the split reporter, from which luciferase signals can be measured. Data is shown as log (RLU) and is the average of three separate experiments. A pDISPLAY vector control was included to set the baseline value to account for background signals. The red boxes indicate the predicted cognate bat receptor for each bat sarbecovirus, where known. **(B)** XY scatter plots for each of the viruses assessed for their receptor usage profile, correlating the log (RLU) of pseudoparticle entry (X) against cell-cell fusion entry (Y), excluding clade II viruses that do not use ACE2 as their entry receptor (RfGB02: *r* = 0.8244, *p* < 0.0001; RhGB07: *r* = 0.9335, *p* < 0.0001; Rc-o319: *r* = 0.9467, *p* < 0.0001; SARS-CoV-2: *r* = 0.6684, *p* = 0.0024; BANAL-20–103: *r* = 0.5308, *p* = 0.0234; BANAL-20–236: *r* = 0.6096, *p* = 0.0072; BANAL-20–52: *r* = 0.7343, *p* = 0.0005; RaTG13: *r* = 0.7719, *p* = 0.0002; SARS-CoV-1: *r* = 0.9005, *p* < 0.0001; WIV-1: *r* = 0.7724, *p* = 0.0002; Rs4231: *r* = 0.8259, *p* < 0.0001). Each data point is labelled with the ACE2-corresponding ACE2 receptor and a Pearson’s correlation calculated with 95% confidence intervals. The Pearson r value and p value are both documented. The data underlying this Figure can be found in S1 Data.
(TIF)

S4 Fig GFP+ syncytia formation of bat sarbecovirus entry by cell–cell fusion. **(A)** GFP and brightfield images of cell-cell fusion of representative bat sarbecovirus Spikes (transfected into HEK293T effector cells stably expressing rLuc-GFP 1–7) with bat ACE2s (transfected into BHK-21 cells expressing rLuc-GFP 8–11) 24 h after co-culture. Four fields of view were taken per well at 10x magnification. Scale bar is shown, 400 µm. **(B)** Analysis of images generated was performed using the IncuCyte S2 software, calculating the total sum of object with fluorescence above the background threshold, measured as total integrated intensity, and expressed as green count units (GCU) µm^2^ and shown in a heatmap. The data underlying this Figure can be found in S1 Data.
(TIF)

S5 Fig Gating strategy and analysis for flow cytometry of ACE2-expressing constructs bound to sarbecovirus RBDs. **(A)** BHK-21 cells were transfected with HA-tagged bat ACE2-expressing constructs (S2 Table) and baited with His tagged-purified RBD proteins for bat sarbecoviruses. Cells were stained with anti-HA PE-conjugated antibody (ACE2) and anti-His APC-conjugated antibody (RBD). Live and singlet BHK-21 cells were gated as PE + APC+ (ACE2 + RBD+), relative to mock-transfected cells (pDISPLAY). **(B)** The SE% Dymax metric was used to calculate the percentage of ACE2 + RBD+ cells, shown for all RBDs tested with our library of bat ACE2 and human ACE2. Representative datasets are shown for mock-transfected cells and raccoon dog ACE2 with SARS-CoV-2 RBD, with their respective SE Dymax % positive value noted. **(C)** Heatmap showing the binding of purified bat sarbecovirus proteins to ACE2s of human, *M. lucifugus Rh. cornutus* by ELISA (pink, EC_50_ µg/ml) or flow cytometry (blue, % ACE2^+^RBD^+^ cells). **(D)** ELISA binding curves of human, *M. lucifugus* and *Rh. alcyone* ACE2s with SARS-CoV-2 and RhGB07 RBDs, depicted also as a heatmap with calculated EC_50_ µg/ml. EC_50_ values were also correlated with entry data from Fig 1D. Scale bars indicate direction of increased binding (pink) or entry (blue). Data is representative of a mean of 3 separate experiments. A cross in a box indicates this data was not obtained. The data underlying this Figure can be found in S1 Data.
(TIF)

S6 Fig Cryo-EM Processing Workflow. **(A)** Processing workflow for the determination of the RhGB07 full-length trimeric spike. **(B)** Cryo-EM density coloured by local resolution of the full-length trimer and of the isolated RBD. Selected regions represented with their cryo-EM density are also shown. **(C)** FSC curve showing a determined resolution for the full-length spike of 2.97 Å **(D)** Viewing direction distribution of particles used in the reconstruction. **(E)** Relative Signal versus Viewing direction plot of particles. Cryo-EM data collection, refinement and validation statistics can be sound in S3 Table.
(TIF)

S7 Fig Comparison of RhGB07 with other sarbecoviruses. **(A)** The C- α trace of the RBD of RhGB07 rendered as balls-and-sticks superimposed with the RBD apostructures of PRD-0038 (PDB 8U29), Khosta-1 (PDB 9J6G), Khosta-2 (PDB: 9J5U) and BtKY72 (PDB 8ZY0). The insets highlight regions of interest of the RBD that are either involved in binding of ACE2 or have presented structural changes across clades. **(B)** The C-α trace of the RBD of RhGB07 rendered as balls-and-sticks superimposed with the RBD of PRD-0038 apo or PRD-0038 bound to R. Alcyone ACE2 (PDB: 8U0T). The insets show a zoomed-in view of two regions of the RBD that show the greatest changes in the position of the C-α of the RBD. The region containing the ins1-loop and Δ4-loop coincides with putative ACE2-interacting residues based on mapping the equivalent residues. **(C)** RBD (top) and NTD (bottom) of RhGB07 cartoon depicted with the cryo-EM density contoured in this region. Residues comprising a putative fatty acid binding pocket as seen in SARS-CoV-2 RBD [39] or a putative biliverdin binding pocket as seen in other coronavirus NTD [40] are highlighted. No density was present in either of these pockets corresponding to a fatty acid or biliverdin, respectively. **(D)** Sequence conservation of selected sarbecoviruses were mapped onto the RhGB07 spike. Areas least conserved are in red and most conserved are blue. The least conserved regions are at the top of the RBD, the NTD and close to a putative polybasic site.
(TIF)

S8 Fig SARS-CoV-2 variants entry and binding with a library of bat ACE2. **(A)** Schematic of the Spike proteins for B.1, Alpha, Delta, Omicron BA.1, Omicron BA.2 and its subvariants BA.4/5, XBB, XBB1.5 and BA.2.86. Key regions of the N-terminal domain (NTD), receptor-binding domain (RBD), S1 and S2 are highlighted and the mutations that arose in these variants have been annotated. The BA.4 and BA.5 Spike is identical, and the difference in XBB1.5 compared to XBB is denoted by an asterisk, which is a reversion of the mutation at position 494 (R493Q). **(B)** Infection of HEK293T cells stably expressing hACE2 with SARS-CoV-2, WT and BA.1 pseudoparticles, along with individual mutants generated of these variants at positions 417, 484, 493 and 501, either singly or in combination. Virus was titrated 5-fold on cells and Firefly luciferase signals measured; a negative control (non-enveloped, NE) was also included. The data underlying this Figure can be found in S1 Data.
(TIF)

S9 Fig Binding of convalescent sera from SARS-CoV-2 infections with sarbecovirus RBDs. **(A)** Heatmap of calculated human IgG binding end-point titres and **(B)** absorbance binding curves for convalescent sera (details in Fig 5A) with SARS-CoV-2, BANAL-20–52, BANAL-20–103, BANAL-20–236, Rs4231, WIV-1, RhGB07 and Rc-o319 RBDs. Boxes with crosses indicate that sample was not tested. Dotted lines represent the limit of detection for the assay (mean + 3SD of no sera control well), with error bars shown for replicate values (mean + SD). The data underlying this Figure can be found in S1 Data.
(TIF)

S10 Fig RBD Chimeras of SARS-CoV-2 and Rs4231 show a switch in ACE2 entry phenotype. **(A)** Infection of HEK293T cells stably expressing hACE2 with SARS-CoV-2, Rs4231 or chimeric pseudoparticles (Rs4231 + SARS-CoV-2 RBD, SARS-CoV-2 + Rs4231 RBD). Virus was titrated 5-fold on cells and Firefly luciferase signals measured; a negative control (non-enveloped, NE) was also included. **(B)** Pseudoparticle entry assay using SARS-CoV-2 and Rs4231 chimeras, on a subset of ACE2s that had striking entry profile in the initial screen (human, *Rh. sinicus*, *Rh. shameli, Rh. macrotis, T. melanopogan,* mouse, Syrian hamster, pig, raccoon dog and marmoset ACE2). The heatmap shows log(RLU) of psuedotype entry, with pDISPLAY included as a control to establish background luciferase signal. The data underlying this Figure can be found in S1 Data.
(TIF)

S11 Fig Binding dendrogram of SARS-CoV-2 monoclonal antibodies analysed by epitope binning. **(A)** Dendrogram represents the relatedness of the epitopes of the antibodies. The cut-off for creating antibody communities is shown by the red line at 0.45. **(B)** Heatmap of antibody blocking and sandwiching interactions. Red boxes indicate blocking interactions, green indicates sandwiching interactions. Self-self blocking is indicated by red boxes with thicker black outlines. Antibodies immobilised as ligands are listed on the left and analytes injected are listed across the top. Where a monoclonal antibody failed to bind as a ligand, it has been excluded.
(TIF)

S12 Fig Binding of sarbceovirus RBDs with monoclonal antibodies isolated from SARS-CoV-2 breakthrough infections. The **(A)** WIV-1-RBD (blue)/ VA14_26 complex (green) (WIV-1 PDB 8WLZ [49]) is shown in cartoon representation. Only the VH and VL domains of the Fab of VA14_26 are shown for clarity. Residues of interest on the WIV-1 RBD that make contacts with VA14_26, R396, K366, A372 are coloured in orange. Contact residues on VA14_26 are coloured in purple. The inset shows a zoomed in highlight of this interface. A surface representation of VA14_26 coloured by electrostatic potential highlights that the WIV-1 RBD residues R396 and K366 that are positively charged bind and localise in a highly negative patch of the Fab demonstrating that VA14_26 interactions are mediated by charge attraction. **(B)** The WIV-1 RBD, along with Rc-o319 (PDB 9W78), SARS-CoV-2 (PDB 7DF3) and RhGB07 (PDB 9RDT), are rendered as cartoon representation or surfaces with electrostatic potential colouring. The residues of interest on the RBD are highlighted on the cartoon representation or of the surface. A critical interacting residue of the VA14_26, E106, is highlighted. This residue is attracted to the positively charged surface of the WIV-1 RBD. Homologous residues present in other RBD, decrease (Rco319) or reverse (RhGB07) this positive charge surface that decreases affinity of the VA14_26 antibody for these RBDs. **(C)** Each plot shows a single mAb binding, coloured by its designated antibody competition group (Group 1 = purple, Group 2 = orange, Group 3 = red, Group 3.5 = blue, Group 4 = green, Group 8 = non-RBD, NTD-specific) in an ELISA using purified RacCS203 RBD. The curves were calculated using a least-squares regression non-linear fit. Binding (EC_50_, µg/ml) titres are shown for each mAb against RacCS203, with limits of detection indicated (lower limit: <0.08g/ml upper limit: 5 µg/ml). Red boxes indicate mAbs that exhibit high binding with all sarbecoviruses tested. **(C)** Representative SPR kinetics binding curves showing the association and dissociation of mAb VA14_26 with bat sarbecovirus RBDs. Each line represents a RBD concentration, starting at 5 µg/ml and titrated 2-fold (8 dilutions). Calculated K_D_ values are also shown. The data underlying this Figure can be found in S1 Data.
(TIF)

S1 Table Bat sarbecovirus Spike constructs used in this study for receptor screens and neutralisation assays. (TIF)

S2 Table Codon-optimised ACE2 expression plasmids used in this study for receptor screens. (TIF)

S3 Table Cryo-EM data collection, refinement, and validation statistics. (TIF)

S4 Table Binding of bat sarbecovirus RBDs and neutralisation of bat sarbecovirus Spike pseudotypes with monoclonal antibodies. (TIF)

S1 Data Underlying primary data gathered in this study. (XLSX)

S1 Raw Images Uncropped western blot images in this study. (PDF)

## Abbreviations

ACE2: angiotensin converting enzyme 2
CSV: Comma-separated values
EC50: effective concentration 50%
EDC: ethyl-3-(3-dimethylaminopropyl)carbodiimide hydrochloride
GCU: green count units
IC50: inhibitory concentration 50%
mAb: monoclonal antibodies
MHRA: Medicines and Healthcare products Regulatory Agency
NaOAc: sodium acetate
NHS: N-hydroxysuccinimide
NTD: N-terminal domain
OD: optical density
RBD: receptor binding domain
RLU: relative light units
SARS: severe acute respiratory syndrome
SPR: surface plasmon resonance
TFS: Thermofisher Scientific
TMB: 3,3’,5,5’- 40 Tetramethylbenzidine
WHO: World Health Organization

## References

[1] Li W, Shi Z, Yu M, Ren W, Smith C, Epstein JH, et al. Bats are natural reservoirs of SARS-like coronaviruses. Science. 2005;310(5748):676–9. doi: 10.1126/science.1118391 16195424

[2] Hu B, Zeng L-P, Yang X-L, Ge X-Y, Zhang W, Li B, et al. Discovery of a rich gene pool of bat SARS-related coronaviruses provides new insights into the origin of SARS coronavirus. PLoS Pathog. 2017;13(11):e1006698. doi: 10.1371/journal.ppat.1006698 29190287 PMC5708621

[3] Ren W, Qu X, Li W, Han Z, Yu M, Zhou P, et al. Difference in receptor usage between severe acute respiratory syndrome (SARS) coronavirus and SARS-like coronavirus of bat origin. J Virol. 2008;82(4):1899–907. doi: 10.1128/JVI.01085-07 18077725 PMC2258702

[4] Wacharapluesadee S, Tan CW, Maneeorn P, Duengkae P, Zhu F, Joyjinda Y, et al. Evidence for SARS-CoV-2 related coronaviruses circulating in bats and pangolins in Southeast Asia. Nat Commun. 2021;12(1):972. doi: 10.1038/s41467-021-21240-1 33563978 PMC7873279

[5] Zhou H, Ji J, Chen X, Bi Y, Li J, Wang Q, et al. Identification of novel bat coronaviruses sheds light on the evolutionary origins of SARS-CoV-2 and related viruses. Cell. 2021;184(17):4380-4391.e14. doi: 10.1016/j.cell.2021.06.008 34147139 PMC8188299

[6] Murakami S, et al. Isolation of bat sarbecoviruses, Japan. Emerg Infect Dis, 2022. 28(12): p. 2500–3. 10.3201/eid2812.220801 PMC9707576 36417954

[7] Murakami S, Kitamura T, Suzuki J, Sato R, Aoi T, Fujii M, et al. Detection and characterization of bat sarbecovirus phylogenetically related to SARS-CoV-2, Japan. Emerg Infect Dis. 2020;26(12):3025–9. doi: 10.3201/eid2612.203386 33219796 PMC7706965

[8] Delaune D, Hul V, Karlsson EA, Hassanin A, Ou TP, Baidaliuk A, et al. A novel SARS-CoV-2 related coronavirus in bats from Cambodia. Nat Commun. 2021;12(1):6563. doi: 10.1038/s41467-021-26809-4 34753934 PMC8578604

[9] Wells HL, et al. The evolutionary history of ACE2 usage within the coronavirus subgenus Sarbecovirus. bioRxiv. 2021. 10.1093/ve/veab007 PMC7928622 33754082

[10] Tan CCS, Trew J, Peacock TP, Mok KY, Hart C, Lau K, et al. Genomic screening of 16 UK native bat species through conservationist networks uncovers coronaviruses with zoonotic potential. Nat Commun. 2023;14(1):3322. doi: 10.1038/s41467-023-38717-w 37369644 PMC10300128

[11] Roelle SM, Shukla N, Pham AT, Bruchez AM, Matreyek KA. Expanded ACE2 dependencies of diverse SARS-like coronavirus receptor binding domains. PLoS Biol. 2022;20(7):e3001738. doi: 10.1371/journal.pbio.3001738 35895696 PMC9359572

[12] Ge X-Y, Li J-L, Yang X-L, Chmura AA, Zhu G, Epstein JH, et al. Isolation and characterization of a bat SARS-like coronavirus that uses the ACE2 receptor. Nature. 2013;503(7477):535–8. doi: 10.1038/nature12711 24172901 PMC5389864

[13] Starr TN, Zepeda SK, Walls AC, Greaney AJ, Alkhovsky S, Veesler D, et al. ACE2 binding is an ancestral and evolvable trait of sarbecoviruses. Nature. 2022;603(7903):913–8. doi: 10.1038/s41586-022-04464-z 35114688 PMC8967715

[14] Lee J, Zepeda SK, Park Y-J, Taylor AL, Quispe J, Stewart C, et al. Broad receptor tropism and immunogenicity of a clade 3 sarbecovirus. Cell Host Microbe. 2023;31(12):1961-1973.e11. doi: 10.1016/j.chom.2023.10.018 37989312 PMC10913562

[15] Conceicao C, Thakur N, Human S, Kelly JT, Logan L, Bialy D, et al. The SARS-CoV-2 Spike protein has a broad tropism for mammalian ACE2 proteins. PLoS Biol. 2020;18(12):e3001016. doi: 10.1371/journal.pbio.3001016 33347434 PMC7751883

[16] Cantoni D, Mayora-Neto M, Thakur N, Elrefaey AME, Newman J, Vishwanath S, et al. Pseudotyped Bat Coronavirus RaTG13 is efficiently neutralised by convalescent sera from SARS-CoV-2 infected patients. Commun Biol. 2022;5(1):409. doi: 10.1038/s42003-022-03325-9 35505237 PMC9065041

[17] Temmam S, Vongphayloth K, Baquero E, Munier S, Bonomi M, Regnault B, et al. Bat coronaviruses related to SARS-CoV-2 and infectious for human cells. Nature. 2022;604(7905):330–6. doi: 10.1038/s41586-022-04532-4 35172323

[18] Wells HL, Letko M, Lasso G, Ssebide B, Nziza J, Byarugaba DK, et al. The evolutionary history of ACE2 usage within the coronavirus subgenus Sarbecovirus. Virus Evol. 2021;7(1):veab007. doi: 10.1093/ve/veab007 33754082 PMC7928622

[19] Yan H, Jiao H, Liu Q, Zhang Z, Xiong Q, Wang B-J, et al. ACE2 receptor usage reveals variation in susceptibility to SARS-CoV and SARS-CoV-2 infection among bat species. Nat Ecol Evol. 2021;5(5):600–8. doi: 10.1038/s41559-021-01407-1 33649547

[20] Feng Y, Yuan M, Powers JM, Hu M, Munt JE, Arunachalam PS, et al. Broadly neutralizing antibodies against sarbecoviruses generated by immunization of macaques with an AS03-adjuvanted COVID-19 vaccine. Sci Transl Med. 2023;15(695):eadg7404. doi: 10.1126/scitranslmed.adg7404 37163615 PMC11032722

[21] Thakur N, Gallo G, Newman J, Peacock TP, Biasetti L, Hall CN, et al. SARS-CoV-2 variants of concern alpha, beta, gamma and delta have extended ACE2 receptor host ranges. J Gen Virol. 2022;103(4):10.1099/jgv.0.001735. doi: 10.1099/jgv.0.001735 35377298

[22] Zhang Y, Wei M, Wu Y, Wang J, Hong Y, Huang Y, et al. Cross-species tropism and antigenic landscapes of circulating SARS-CoV-2 variants. Cell Rep. 2022;38(12):110558. doi: 10.1016/j.celrep.2022.110558 35303476 PMC8898742

[23] Zhou J, Peacock TP, Brown JC, Goldhill DH, Elrefaey AME, Penrice-Randal R, et al. Mutations that adapt SARS-CoV-2 to mink or ferret do not increase fitness in the human airway. Cell Rep. 2022;38(6):110344. doi: 10.1016/j.celrep.2022.110344 35093235 PMC8768428

[24] Niu Z, Zhang Z, Gao X, Du P, Lu J, Yan B, et al. N501Y mutation imparts cross-species transmission of SARS-CoV-2 to mice by enhancing receptor binding. Signal Transduct Target Ther. 2021;6(1):284. doi: 10.1038/s41392-021-00704-2 34315847 PMC8313414

[25] Pickering B, Lung O, Maguire F, Kruczkiewicz P, Kotwa JD, Buchanan T, et al. Divergent SARS-CoV-2 variant emerges in white-tailed deer with deer-to-human transmission. Nat Microbiol. 2022;7(12):2011–24. doi: 10.1038/s41564-022-01268-9 36357713 PMC9712111

[26] Kuchipudi SV, Surendran-Nair M, Ruden RM, Yon M, Nissly RH, Vandegrift KJ, et al. Multiple spillovers from humans and onward transmission of SARS-CoV-2 in white-tailed deer. Proc Natl Acad Sci U S A. 2022;119(6):e2121644119. doi: 10.1073/pnas.2121644119 35078920 PMC8833191

[27] Hale VL, Dennis PM, McBride DS, Nolting JM, Madden C, Huey D, et al. SARS-CoV-2 infection in free-ranging white-tailed deer. Nature. 2022;602(7897):481–6. doi: 10.1038/s41586-021-04353-x 34942632 PMC8857059

[28] Thakur N, Conceicao C, Isaacs A, Human S, Modhiran N, McLean RK, et al. Micro-fusion inhibition tests: quantifying antibody neutralization of virus-mediated cell-cell fusion. J Gen Virol. 2021;102(1):jgv001506. doi: 10.1099/jgv.0.001506 33054904 PMC8116787

[29] Thakur N, Gallo G, Elreafey AME, Bailey D. Production of recombinant replication-defective lentiviruses bearing the SARS-CoV or SARS-CoV-2 attachment spike glycoprotein and their application in receptor tropism and neutralisation assays. Bio Protoc. 2021;11(21):e4249. doi: 10.21769/BioProtoc.4249 34859135 PMC8595443

[30] Newman J, Thakur N, Peacock TP, Bialy D, Elrefaey AME, Bogaardt C, et al. Neutralizing antibody activity against 21 SARS-CoV-2 variants in older adults vaccinated with BNT162b2. Nat Microbiol. 2022;7(8):1180–8. doi: 10.1038/s41564-022-01163-3 35836002 PMC9352594

[31] Ni D, Turelli P, Beckert B, Nazarov S, Uchikawa E, Myasnikov A, et al. Cryo-EM structures and binding of mouse and human ACE2 to SARS-CoV-2 variants of concern indicate that mutations enabling immune escape could expand host range. PLoS Pathog. 2023;19(4):e1011206. doi: 10.1371/journal.ppat.1011206 37018380 PMC10109501

[32] Jumper J, Evans R, Pritzel A, Green T, Figurnov M, Ronneberger O, et al. Highly accurate protein structure prediction with AlphaFold. Nature. 2021;596(7873):583–9. doi: 10.1038/s41586-021-03819-2 34265844 PMC8371605

[33] Millán C, McCoy AJ, Terwilliger TC, Read RJ. Likelihood-based docking of models into cryo-EM maps. Acta Crystallogr D Struct Biol. 2023;79(Pt 4):281–9. doi: 10.1107/S2059798323001602 36920336 PMC10071562

[34] Emsley P, Lohkamp B, Scott WG, Cowtan K. Features and development of Coot. Acta Crystallogr D Biol Crystallogr. 2010;66(Pt 4):486–501. doi: 10.1107/S0907444910007493 20383002 PMC2852313

[35] Meng EC, et al. UCSF ChimeraX: Tools for structure building and analysis. Protein Science. 2023;32(11):e4792. 10.1002/pro.4792 PMC10588335 37774136

[36] Guo H, Hu B, Si H-R, Zhu Y, Zhang W, Li B, et al. Identification of a novel lineage bat SARS-related coronaviruses that use bat ACE2 receptor. Emerg Microbes Infect. 2021;10(1):1507–14. doi: 10.1080/22221751.2021.1956373 34263709 PMC8344244

[37] Wacharapluesadee S, Tan CW, Maneeorn P, Duengkae P, Zhu F, Joyjinda Y, et al. Evidence for SARS-CoV-2 related coronaviruses circulating in bats and pangolins in Southeast Asia. Nat Commun. 2021;12(1):972. doi: 10.1038/s41467-021-21240-1 33563978 PMC7873279

[38] Delaune D, Hul V, Karlsson EA, Hassanin A, Ou TP, Baidaliuk A, et al. A novel SARS-CoV-2 related coronavirus in bats from Cambodia. Nat Commun. 2021;12(1):6563. doi: 10.1038/s41467-021-26809-4 34753934 PMC8578604

[39] Toelzer C, Gupta K, Yadav SKN, Borucu U, Davidson AD, Kavanagh Williamson M, et al. Free fatty acid binding pocket in the locked structure of SARS-CoV-2 spike protein. Science. 2020;370(6517):725–30. doi: 10.1126/science.abd3255 32958580 PMC8050947

[40] Wang J, Ma Y, Li Z, Yuan H, Liu B, Li Z, et al. SARS-related coronavirus S-protein structures reveal synergistic RBM interactions underpinning high-affinity human ACE2 binding. Sci Adv. 2025;11(11):eadr8772. doi: 10.1126/sciadv.adr8772 40085715 PMC11908486

[41] Wang Q, Noettger S, Xie Q, Pastorio C, Seidel A, Müller JA, et al. Determinants of species-specific utilization of ACE2 by human and animal coronaviruses. Commun Biol. 2023;6(1):1051. doi: 10.1038/s42003-023-05436-3 37848611 PMC10582019

[42] Song Z, et al. From SARS to MERS, thrusting coronaviruses into the spotlight. Viruses. 2019;11(1). 10.3390/v11010059 PMC6357155 30646565

[43] Smyth DS, Trujillo M, Gregory DA, Cheung K, Gao A, Graham M, et al. Tracking cryptic SARS-CoV-2 lineages detected in NYC wastewater. Nat Commun. 2022;13(1):635. doi: 10.1038/s41467-022-28246-3 35115523 PMC8813986

[44] Tan CCS, Lam SD, Richard D, Owen CJ, Berchtold D, Orengo C, et al. Transmission of SARS-CoV-2 from humans to animals and potential host adaptation. Nat Commun. 2022;13(1):2988. doi: 10.1038/s41467-022-30698-6 35624123 PMC9142586

[45] Kok K-H, Wong S-C, Chan W-M, Wen L, Chu AW-H, Ip JD, et al. Co-circulation of two SARS-CoV-2 variant strains within imported pet hamsters in Hong Kong. Emerg Microbes Infect. 2022;11(1):689–98. doi: 10.1080/22221751.2022.2040922 35135441 PMC8890519

[46] Seow J, Shalim ZA, Graham C, Kimuda S, Pillai A, Lechmere T, et al. Broad and potent neutralizing antibodies are elicited in vaccinated individuals following Delta/BA.1 breakthrough infection. mBio. 2023;14(5):e0120623. doi: 10.1128/mbio.01206-23 37747187 PMC10653880

[47] Seow J, Graham C, Hallett SR, Lechmere T, Maguire TJA, Huettner I, et al. ChAdOx1 nCoV-19 vaccine elicits monoclonal antibodies with cross-neutralizing activity against SARS-CoV-2 viral variants. Cell Rep. 2022;39(5):110757. doi: 10.1016/j.celrep.2022.110757 35477023 PMC9010245

[48] Graham C, Seow J, Huettner I, Khan H, Kouphou N, Acors S, et al. Neutralization potency of monoclonal antibodies recognizing dominant and subdominant epitopes on SARS-CoV-2 Spike is impacted by the B.1.1.7 variant. Immunity. 2021;54(6):1276-1289.e6. doi: 10.1016/j.immuni.2021.03.023 33836142 PMC8015430

[49] Upadhyay A, et al. Mechanisms for cross-neutralisation of diverse bat sarbecoviruses. bioRxiv. 2025;:2025.09.17.674891. doi: 10.1101/2025.09.17.674891

[50] Kosugi Y, Matsumoto K, Lytras S, Plianchaisuk A, Tolentino JE, Fujita S, et al. Molecular basis of sarbecovirus evolution and receptor tropism in natural hosts, potential intermediate hosts, and humans. Cell Rep. 2025;44(9):116220. doi: 10.1016/j.celrep.2025.116220 40934083

[51] Si J-Y, Chen Y-M, Sun Y-H, Gu M-X, Huang M-L, Shi L-L, et al. Sarbecovirus RBD indels and specific residues dictating multi-species ACE2 adaptiveness. Nat Commun. 2024;15(1):8869. doi: 10.1038/s41467-024-53029-3 39402048 PMC11473667

[52] Roelle SM, Shukla N, Pham AT, Bruchez AM, Matreyek KA. Expanded ACE2 dependencies of diverse SARS-like coronavirus receptor binding domains. PLoS Biol. 2022;20(7):e3001738. doi: 10.1371/journal.pbio.3001738 35895696 PMC9359572

[53] Seifert SN, Bai S, Fawcett S, Norton EB, Zwezdaryk KJ, Robinson J, et al. An ACE2-dependent Sarbecovirus in Russian bats is resistant to SARS-CoV-2 vaccines. PLoS Pathog. 2022;18(9):e1010828. doi: 10.1371/journal.ppat.1010828 36136995 PMC9498966

[54] Wang J, et al. Structural basis for Sarbecovirus Rc-o319 spike adaptation to Rhinolophus cornutus bat ACE2 and constraints on switching to human ACE2. bioRxiv. 2026;:2025.09.17.676762. 10.1371/journal.ppat.1014245 PMC13232947 42166485

[55] Wu Z, Han Y, Wang Y, Liu B, Zhao L, Zhang J, et al. A comprehensive survey of bat sarbecoviruses across China in relation to the origins of SARS-CoV and SARS-CoV-2. Natl Sci Rev. 2022;10(6):nwac213. doi: 10.1093/nsr/nwac213 37425654 PMC10325003

[56] Gallo G, Di Nardo A, Lugano D, et al. Heart-nosed bat alphacoronaviruses use human CEACAM6 to enter cells. Nature. 2026;653:180–9. doi: 10.1038/s41586-026-10394-x PMC13149331 42020746

[57] Hsueh F-C, Shi K, Mendoza A, Bu F, Zhang W, Aihara H, et al. Structural basis for raccoon dog receptor recognition by SARS-CoV-2. PLoS Pathog. 2024;20(5):e1012204. doi: 10.1371/journal.ppat.1012204 38709834 PMC11098500

[58] Shukla N, Roelle SM, Snell JC, DelSignore O, Bruchez AM, Matreyek KA. Pseudotyped virus infection of multiplexed ACE2 libraries reveals SARS-CoV-2 variant shifts in receptor usage. PLoS Pathog. 2024;20(5):e1012044. doi: 10.1371/journal.ppat.1012044 38768238 PMC11142672

[59] Melin AD, Janiak MC, Marrone F 3rd, Arora PS, Higham JP. Comparative ACE2 variation and primate COVID-19 risk. Commun Biol. 2020;3(1):641. doi: 10.1038/s42003-020-01370-w 33110195 PMC7591510

[60] Halfmann PJ, Loeffler K, Duffy A, Kuroda M, Yang JE, Wright ER, et al. Broad protection against clade 1 sarbecoviruses after a single immunization with cocktail spike-protein-nanoparticle vaccine. Nat Commun. 2024;15(1):1284. doi: 10.1038/s41467-024-45495-6 38346966 PMC10861510

[61] Li P, Guo R, Liu Y, Zhang Y, Hu J, Ou X, et al. The Rhinolophus affinis bat ACE2 and multiple animal orthologs are functional receptors for bat coronavirus RaTG13 and SARS-CoV-2. Sci Bull (Beijing). 2021;66(12):1215–27. doi: 10.1016/j.scib.2021.01.011 33495713 PMC7816560

[62] Wrobel AG, Benton DJ, Xu P, Roustan C, Martin SR, Rosenthal PB, et al. SARS-CoV-2 and bat RaTG13 spike glycoprotein structures inform on virus evolution and furin-cleavage effects. Nat Struct Mol Biol. 2020;27(8):763–7. doi: 10.1038/s41594-020-0468-7 32647346 PMC7610980

