# Supplementary material for: Breadth of ACE2 receptor usage predicts host range and antigenic relatedness across bat sarbecoviruses
Source: PLoS Biol. 2026 Oct 1;24(10):e3003944. doi: 10.1371/journal.pbio.3003944 (PMC13630225; doi:10.1371/journal.pbio.3003944)

# Supp Figure 2B
# Uncropped Western blots
# Anti-HA antibody (ACE2s)

ThermoScientific PageRuler Plus prestained protein ladder

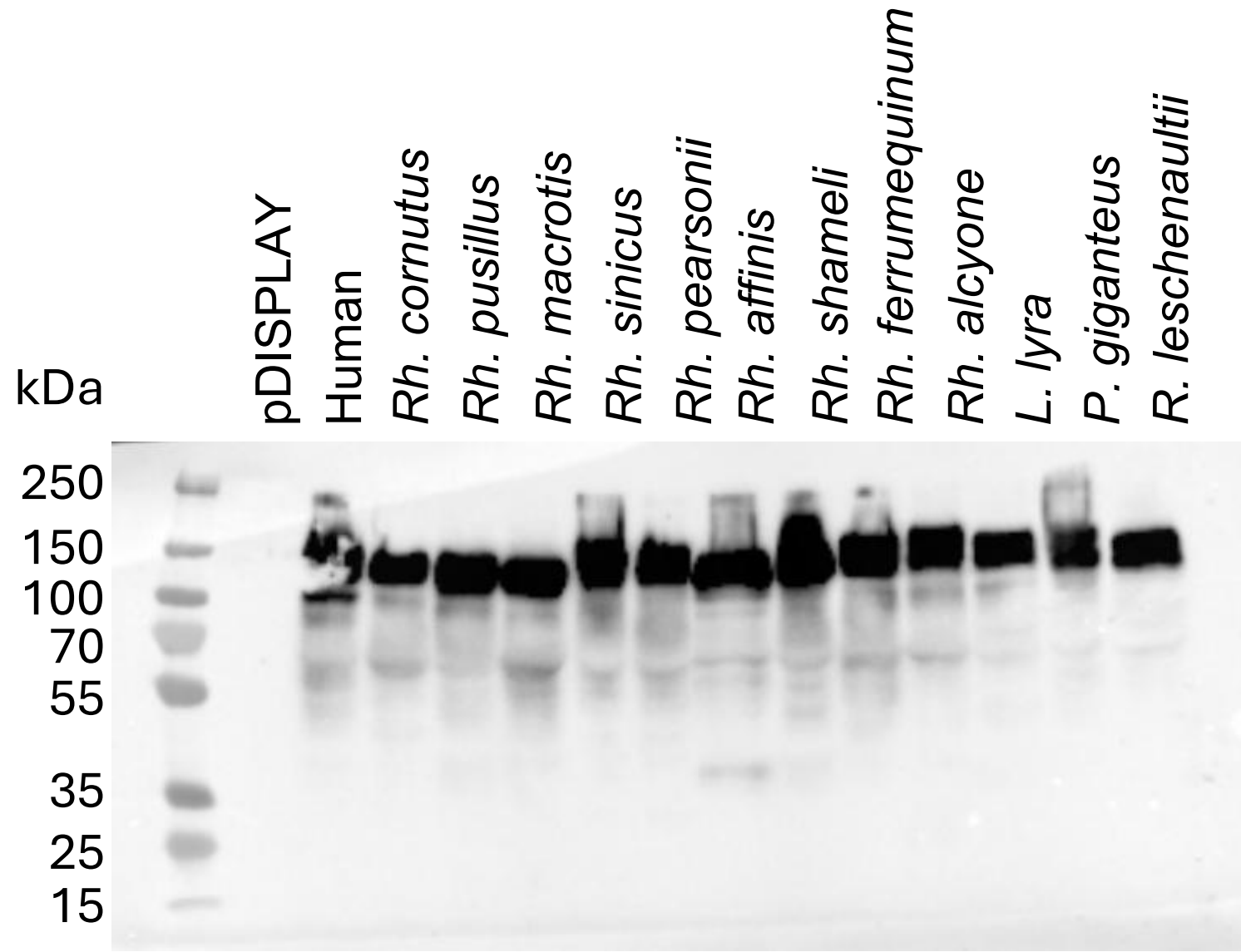


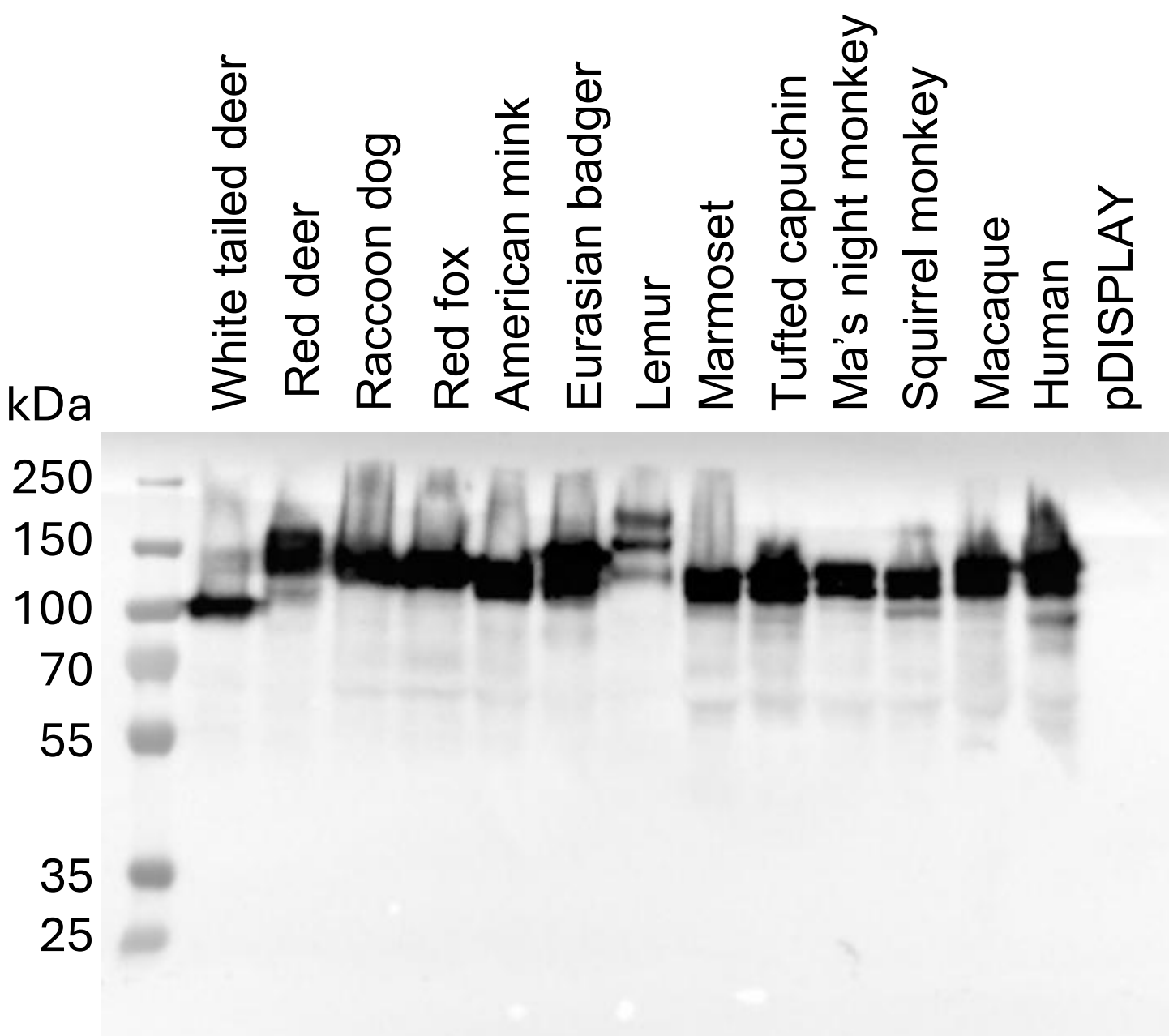


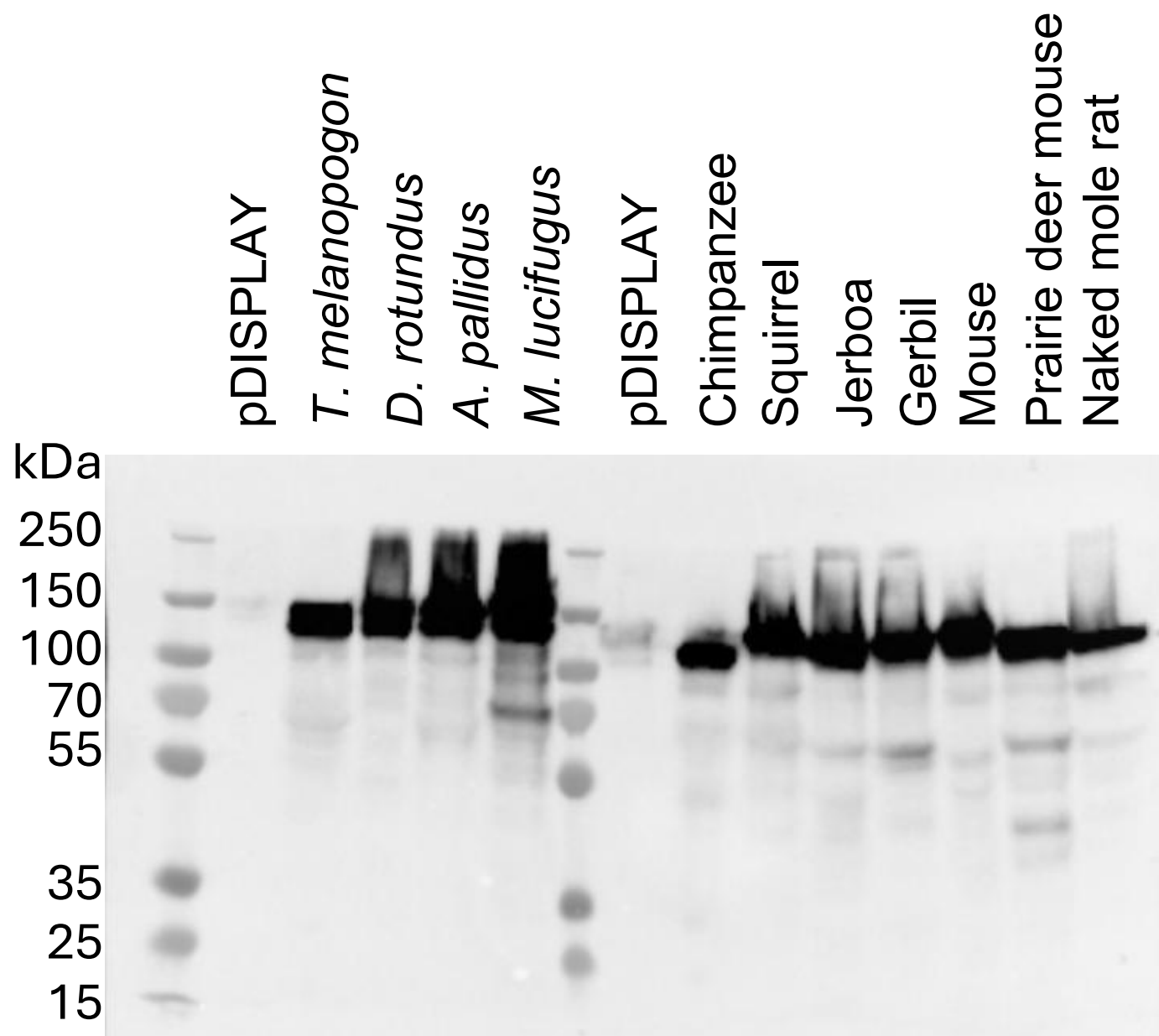

# Supp Figure 2B
# Uncropped Western blots
# GAPDH (ACE2s)

ThermoScientific PageRuler Plus prestained protein ladder

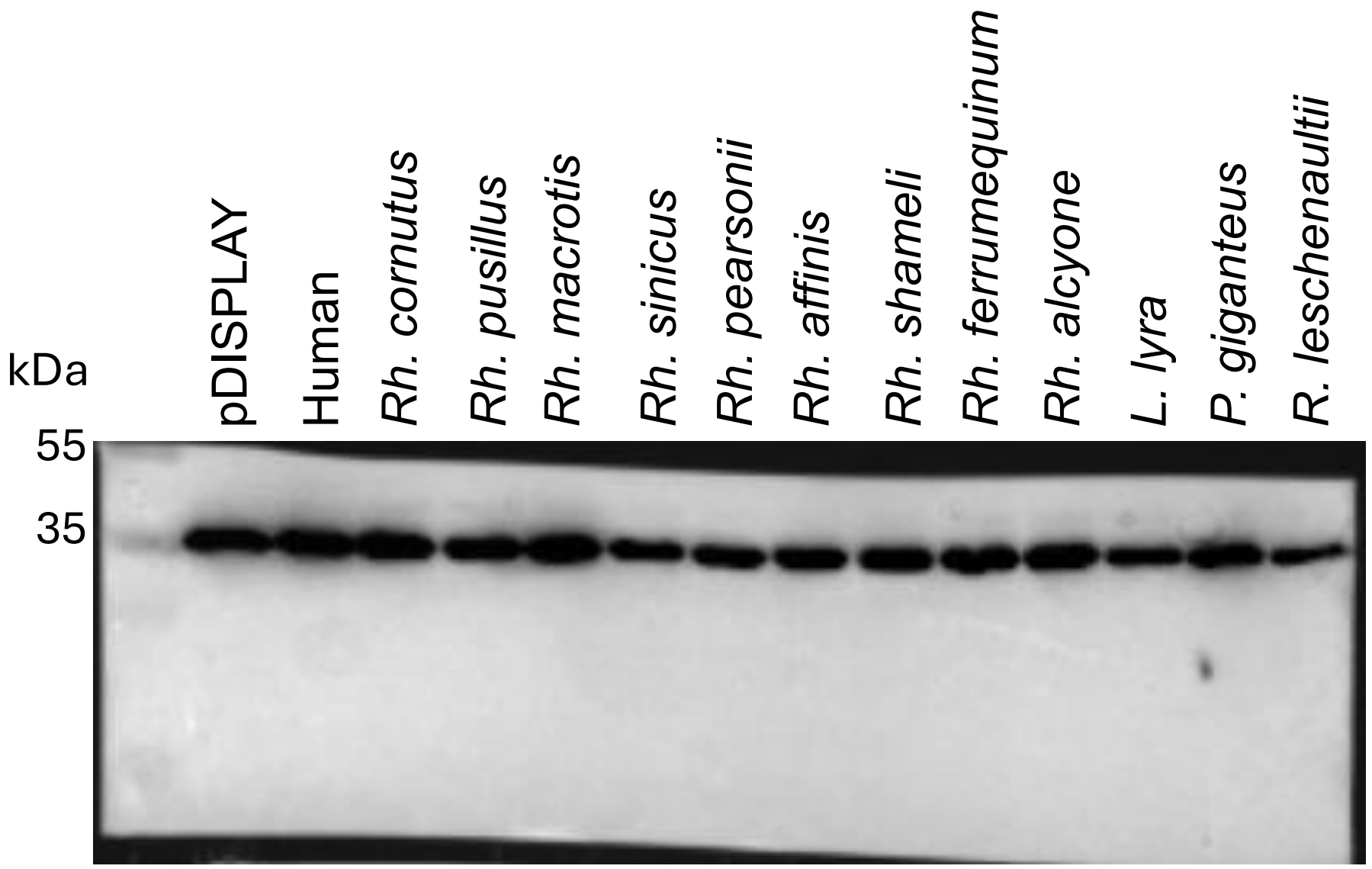


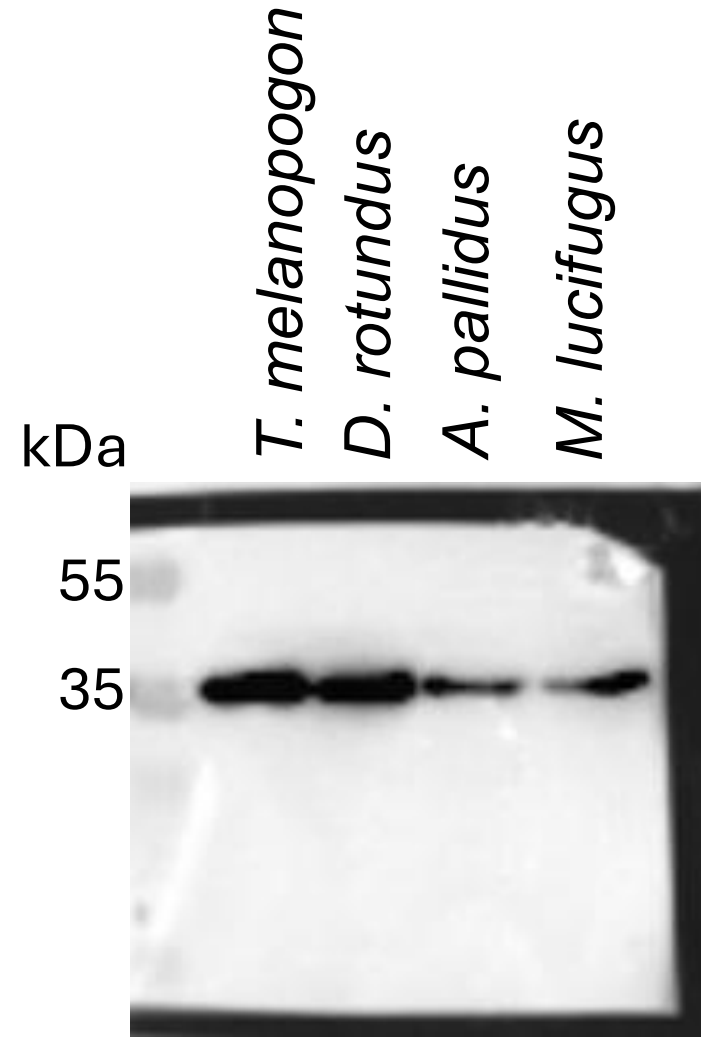


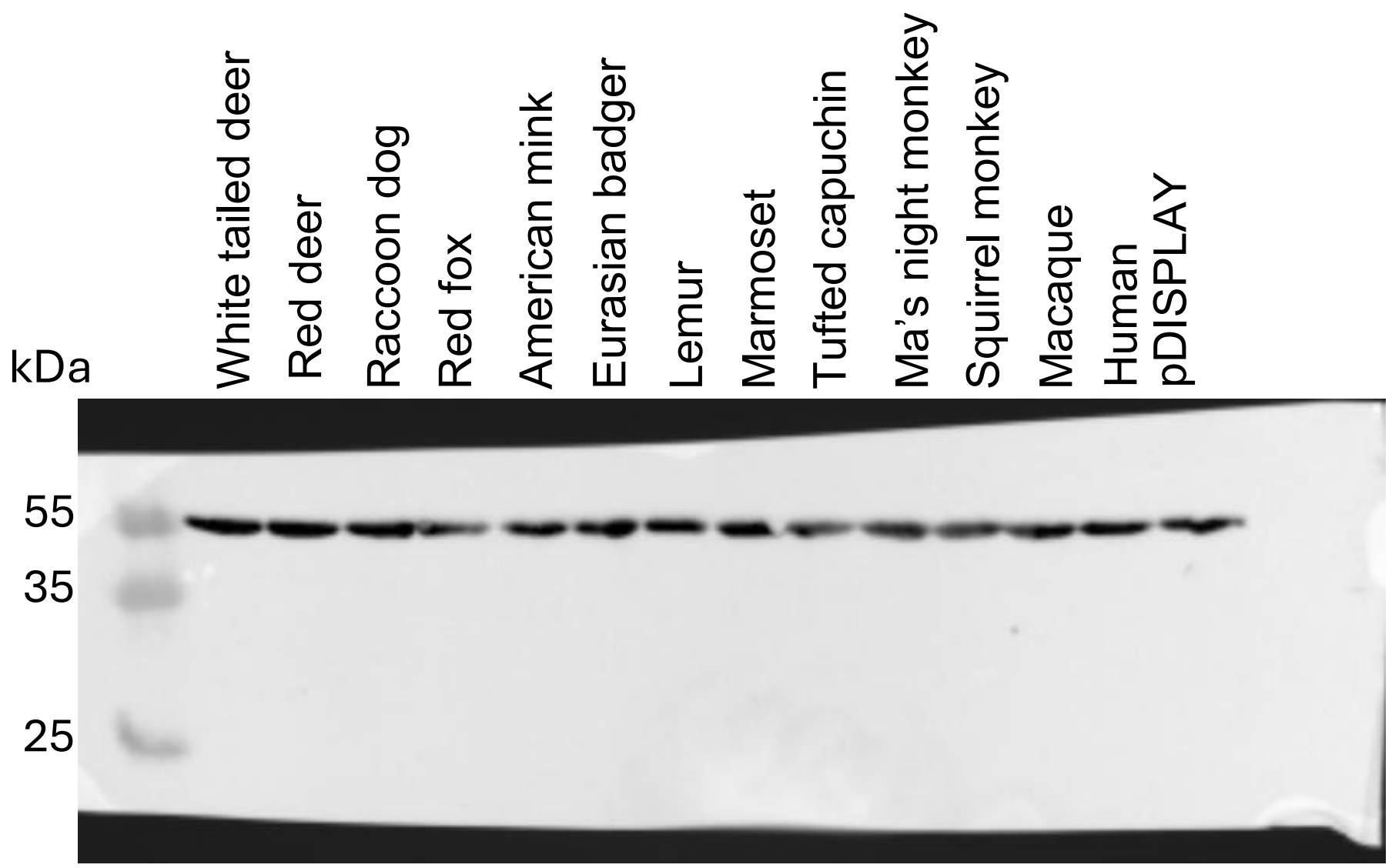


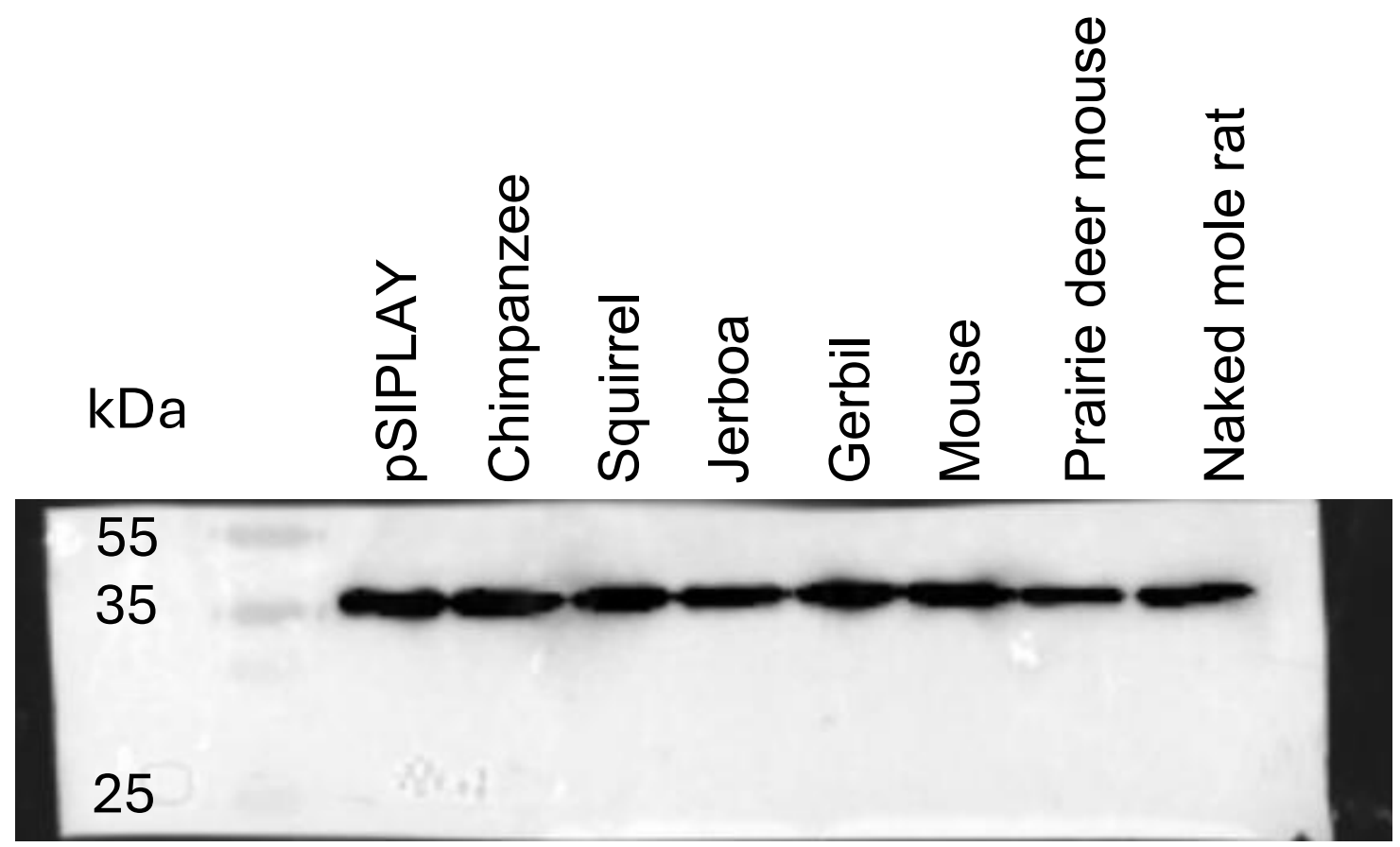

**Supp Figure 2C**
**Uncropped Western blots**
**Anti-FLAG, p24 (Purified Spikes)**

ThermoScientific PageRuler Plus prestained protein ladder

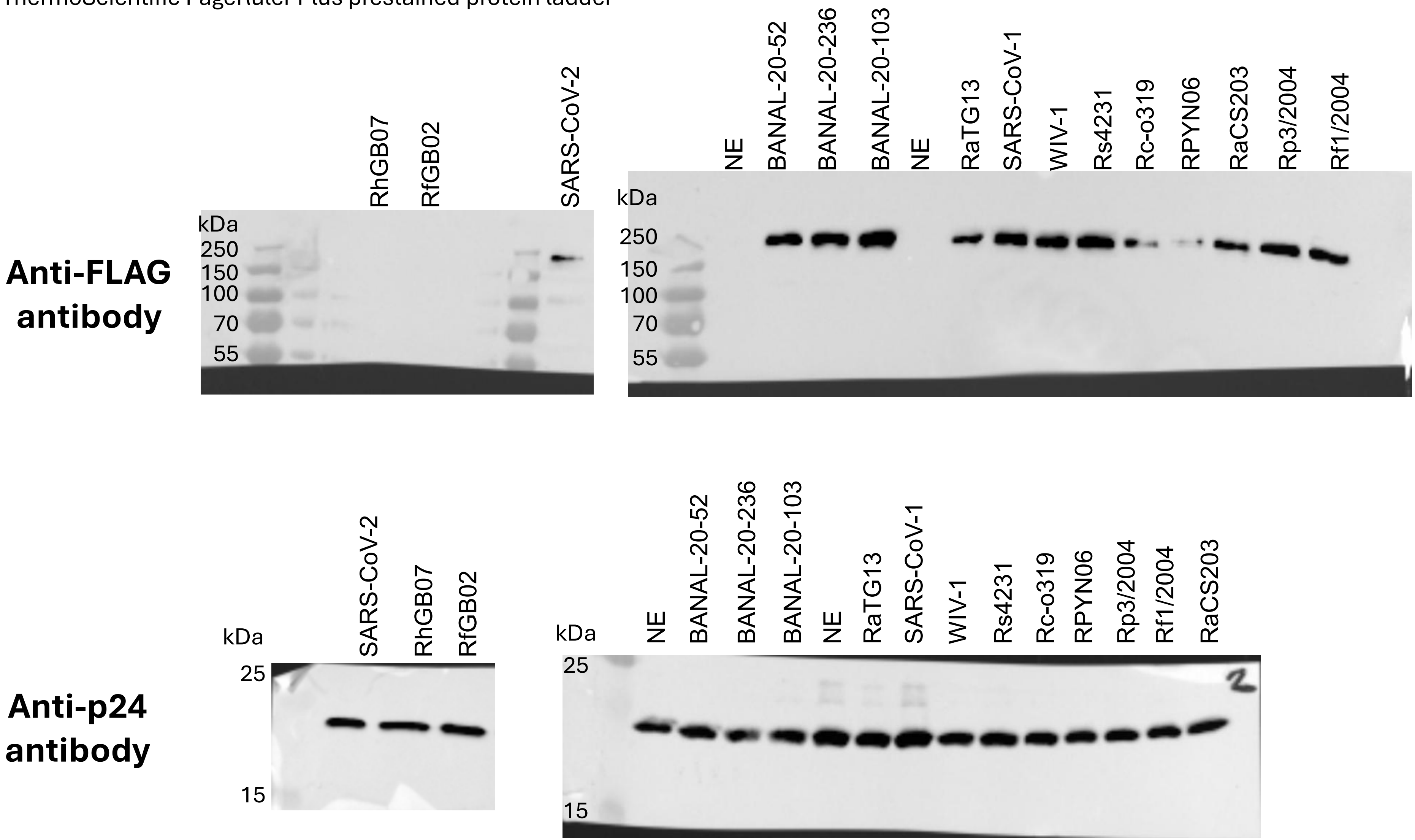

# Supp Figure 2B
# Uncropped Western blots
# Anti-Spike antibody (Purified Spikes)

ThermoScientific PageRuler Plus prestained protein ladder

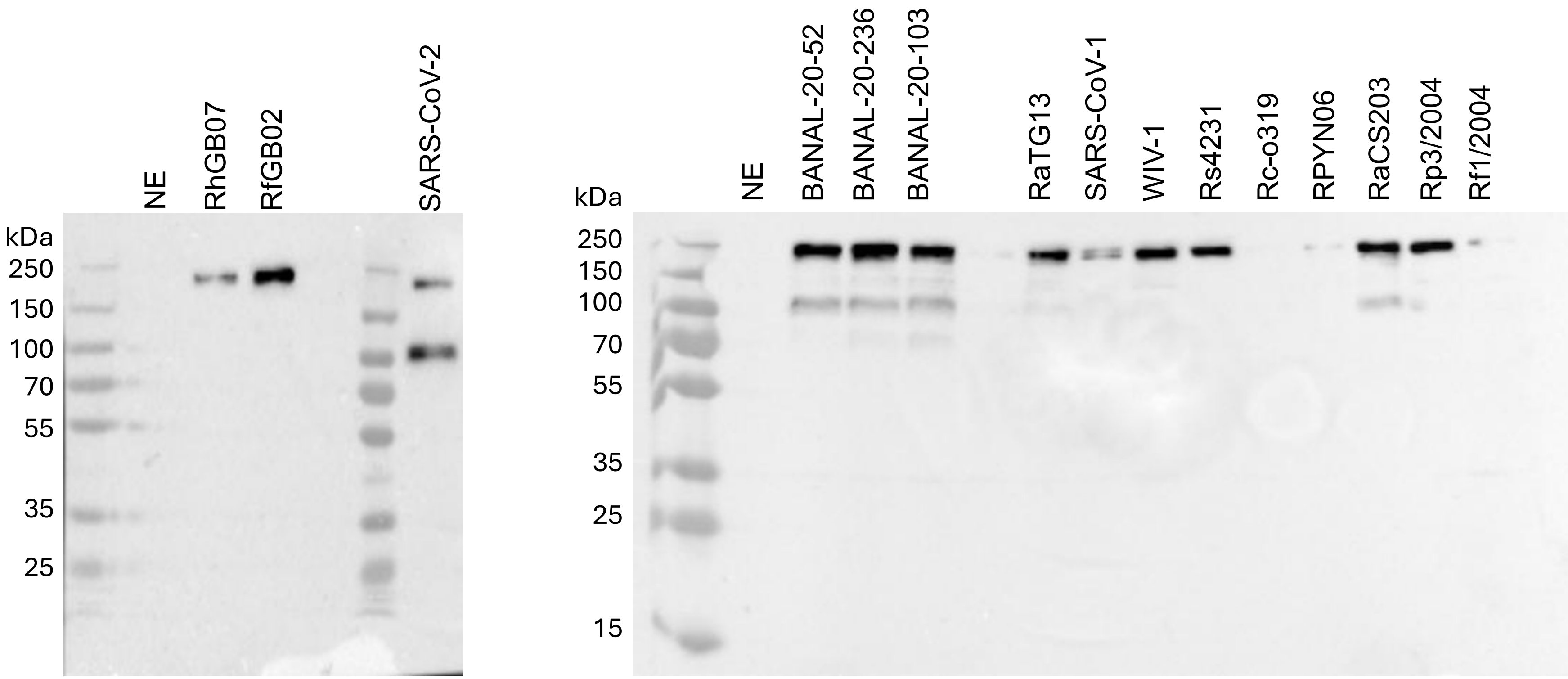

Supplement: Supplementary file 1 [file pbio.3003944.s018.pdf]
