# Supplementary material for: Breadth of ACE2 receptor usage predicts host range and antigenic relatedness across bat sarbecoviruses
Source: PLoS Biol. 2026 Oct 1;24(10):e3003944. doi: 10.1371/journal.pbio.3003944 (PMC13630225; doi:10.1371/journal.pbio.3003944)
Supplement: Supplementary file 2 [file pbio.3003944.s021.pdf]

PBIOLOGY-D-25-03032R1: Response to Reviewers: May 2026

Dear Editors and Reviewers,

Firstly, we would like to thank you for your positive comments on the quality of our work and for recognizing the novelty of our study and its importance in improving our understanding of sarbecovirus host range, antigenicity and risk. We are also grateful for the suggestions made and questions raised, which we address point-by-point below. Further, the requirement from the journal to include relevant literature that was published since initial submission has resulted in changes to Figure 1A to include recently available bat sarbecovirus RBD sequences. We also attach a tracked changes version of the amended manuscript.

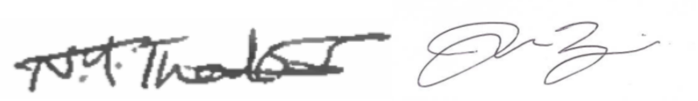

Yours sincerely,

Drs. Nazia Thakur and Dalan Bailey

On behalf of all co-authors 1/5/2026

Editor's note:

In light of the reviews, which you will find at the end of this email, we would like to invite you to revise the work to thoroughly address the reviewers' reports. As you will see below, majority of reviewers are positive about the relevance and novelty of the study, yet some concerns have raised during revision regarding missing data to support the hypotheses.

1. **Reviewer 1 requests validation of the RBD/ACE2 interaction hypothesis, to provide further support of the RBD vs human/bat ACE2 interactions like protein purification and mutations, and to clarify some claims.**
2. **Reviewer 2 raised some concerns about the structural analysis, asking for quantitative RBD–ACE2 binding data, raw fusion images, clarification of missing ACE2-bound structures, corrections to structural comparisons and FSC curves, resolution of contradictions in antibody binding versus neutralization, and integration of recent literature on RBM architecture and ACE2-binding interfaces. S/He also mentions that obtaining at least one RhGB07 Spike structure would be ideal to have more appropriate controls.**
3. **Reviewer 3 notes that much of the ACE2-usage background is already known and argues that the manuscripts novelty depends on strengthening the RhGB07 structural and functional characterization, requesting binding affinity data, structural analysis, clearer pseudovirus normalization, inclusion of SARS-CoV-1 in antigenicity analyses, and broader sampling of SARS-CoV-1–related sarbecoviruses.**
4. **Reviewer 4 had concerns with the cryo-EM analysis, such as the low fraction of particles used, the use of strict C3 symmetry potentially obscuring RBD conformations, lack of evidence for ACE2 presence on grids, absence of biophysical validation of Spike–ACE2 interaction, and missing atomic model validation. Additionally, the reviewer questions the epitope binning approach using full IgGs**

instead of Fabs and the fairness of structural comparisons across apo and ACE2-bound states.

We agree with all reviewer concerns and would require some additional experimental revisions to address them, as we consider that this would strengthen the work.

We thank the PLOS Biology Editors for their summary and the reviewers for their enthusiasm for the paper. We have addressed these major points in detail below in our point-by-point responses.

Reviewer #1:

This manuscript maps receptor usage, host range and antigenicity across a panel of bat sarbecovirus spikes and mammalian ACE2 orthologues. It shows that the clade I viruses (which includes SARS-CoV-2 and BANAL isolates) tend to be generalists and able to use receptors across species while clade III and V viruses are more specialist for particular bat ACE2s. They also demonstrate a cryo-EM structure of the clade-III Spike that shows differences in receptor tropism and helps to explain the shifts in ACE2 usage as SARS-CoV-2 evolved. The work is well done and agrees with previously published work showing the generalist ACE2 binding of SARS-CoV-2.

Major comments below:

1. There is little data supporting the RBD/ACE2 interaction hypothesis. The RhGB07 cryo-EM structure is well done, but the authors discuss that specific deletions and glycans explain specialist tropism without validation. Please discuss the confidence in these conclusions or include additional experiments to validate the findings.

We thank the reviewer for their comments and have now included additional experiments to address this; specifically our conclusions that deletions in RhGB07 are deterministic in its ‘specialist’ tropism. In particular, we focused on the deletion in the Δ4-loop (445-449 loop; SARS-CoV-2 numbering), which has been shown to be important for ACE2 binding, particularly via Y449 which forms hydrogen bonds with D38 and Q42 in human ACE2, and G446 which also forms a hydrogen bond with Q42 in human ACE2. We first generated full RBD swaps between RhGB07 and SARS-CoV-2, noting an increase in the chimeric RhGB07 pseudotype’s use of human ACE2 when containing a SARS-CoV-2 RBD ($\text{RhGB07}_{\text{SARS-CoV-2 RBD}}$) and a corresponding decrease in chimeric SARS-CoV-2 pseudotype’s use of human ACE2 with a RhGB07 RBD (SARS-CoV-2 $_{\text{RhGB07 RBD}}$) (**new Figure 2C, below**). Targeted mutants focusing on the Δ4-loop (RhGB07 437_438insGGNY or SARS-CoV-2 Δ446-449) recapitulated this phenotype (**new Figure 2C, below**), allowing us to partially narrow down tropism to this particular region. We also provide corresponding binding data by ELISA, wherein this insertion or deletion was introduced into recombinant RhGB07 or SARS-CoV-2 RBDs, respectively. In these assays an increase in RhGB07 437_438insGGNY RBD binding was observed for human ACE2, and a decrease in human ACE2 binding with SARS-CoV-2 Δ446-449 RBD (**new Figure 2D, below**). Additional mutations to this RBM of the RhGB07 RBD were generated to examine the wider significance in the context of generalist and specialist bat sarbecoviruses for a smaller panel of bat ACE2s. Mutants to make RhGB07 more “SARS-CoV-2-like, SARS-CoV-1-like, RaTG13-like, Rs4231-like or Rc-o319-like” were examined. These data highlighted however, that this insertion alone was not entirely deterministic in host range (**new**

Figure 2E, below), and suggests that other changes in the RBD and/or Spike are important for the receptor tropism observed for the sarbecoviruses tested.

Wang J et al., (SARS-related coronavirus S-protein structures reveal synergistic RBM interactions underpinning high-affinity human ACE2 binding - PMC) similarly show the importance of this Δ4-loop loop in ACE2 usage for another clade III bat coronavirus BM48-31, wherein insertion of KGGY residues in this loop was able to increase human ACE2 binding, detected by BLI. Importantly, all 4 amino acid insertions were required to see this phenotype, as increased human ACE2 binding was not observed when only inserting KG or GY (in combination with other mutations). However, the authors also show deletion of G447 and Y449 (SARS-CoV-2 numbering) in another clade III virus RBD, BtKY72, prevents binding with human ACE2 but compensatory mutations could restore ACE2 usage (e.g., residue 493 (SARS-CoV-2 numbering) becomes more important). We have added these additional figure panels to Figure 2 and altered the text to summarise these findings to clarify that these deletions are only partially responsible for the specialist ACE2 tropism of RhGB07 (lines 504-541), which reads:

*"To examine the extent to which the Δ4-loops contribute to the restricted receptor tropism of RhGB07, mutants of RhGB07 and SARS-CoV-2 were generated for comparison. Firstly, full RBD chimeric swaps were generated, resulting in, as expected, a modest increase in human ACE2 usage for RhGB07$_{SARS\text{-}CoV\text{-}2\ RBD}$ pseudotypes, and a slight decrease in pseudotype entry for SARS-CoV$_{RhGB07\ RBD}$ (**Figure 2C**). Next, the direct importance of the Δ4-loop was investigated, by inserting the SARS-CoV-2 GGNY residues between positions 437 and 438 in the RhGB07 Spike (RhGB07 437_438insGGNY) or deleting the corresponding region in SARS-CoV-2 (SARS-CoV-2 Δ446-449). A further decrease in human ACE2 usage was noted with the SARS-CoV Δ446-449 mutant, and a similar human ACE2 usage for RhGB07 437_438insGGNY, when compared to RhGB07$_{SARS\text{-}CoV\text{-}2\ RBD}$ (**Figure 2C**). This indicates that this region is important in mediating virus entry, likely due to altered ACE2 binding affinity, confirmed by ELISA (**Figure 2D**). To examine the wider significance of this result to sarbecovirus biology, further mutants of RhGB07 were generated to examine whether host range could also be altered by mutating the Δ4-loop region to reflect other generalist sarbecoviruses. RhGB07 was mutated to make it more SARS-CoV-2-like (RhGB07 437_438insVGGNY), SARS-CoV-1-like (RhGB07 437_438insSTGNY), Rs4231-like (RhGB07 437_438insTSGNY), RaTG13-like (RhGB07 437_438insEGGNF), or Rc-o319-like (RhGB07 437_438insTSGNF). Importantly, the receptor tropism of these mutants was examined with a small panel ACE2s that were previously shown to be differentially used by our bat sarbecovirus (**Figure 1D**). The use of human, Rh.alycone and M. lucifugus ACE2s increased with all mutants except the SARS-CoV-1-like mutant (RhGB07 437_438insSTGNY), which remained similar to wildtype RhGB07 (**Figure 2E**). Interestingly, the RhGB07 437_438insGGNY mutation alone was insufficient to broaden bat ACE2 tropism, but additional mutations to make the virus SARS-CoV-2-like (RhGB07 437_438insVGGNY) (generalist), increased usage of Rh. pusillus, Rh affinis, Rh. ferrumequinum and Rh. cornutus ACE2s (**Figure 2E**). highlighting the importance of the Q437V substitution. Also of note, an increased usage of Rh. affinis ACE2 was observed with the SARS-CoV-1-like mutant (RhGB07 437_438insSTGNY), but not the other bat ACE2s examined, mirroring the ACE2-usage profile of SARS-CoV-1 (**Figure 1D**). However, while the Rc-o319-like mutant (RhGB07 437_438insTSGNF) also exhibited an increase in Rh. affinis ACE2 usage, this was not observed for the RaTG13-like mutant (RhGB07 437_438insEGGNF) or Rs4231-like mutant (RhGB07 437_438insTSGNY), despite **Figure 1D** showing both RaTG13 and Rs4231 sarbecovirus pseudotypes are able to enter cells overexpressing Rh. affinis ACE2 (**Figure 2E**). Additionally, RhGB07 437_438QGGNF and RhGB07 437_438insEGGNY mutants alone (reflecting the Δ4-loop region sequences of RaTG13 and Rc-o319) were unable to increase receptor tropism, and in fact*

*exhibited a decrease in human, Rh. alcyone and M. lucifugus ACE2 usage (**Figure 2E**). These data indicate a synergistic role for these residues in the Δ4-loop, but also the likely involvement of other residues in the RBD or Spike that contribute to receptor tropism."*

We have also added a sentence to the discussion (lines 803 – 811):

*"In our study, deletion of the 446-449 region in SARS-CoV-2 and insertion of these residues in RhGB07 indeed showed an increase in entry and binding with human (**Figure 2C and D**). However, RhGB07 mutants generated to mimic other bat sarbecoviruses only resulted in an increase in bat ACE2 tropism for the broad generalist viruses (RhGB07 437_438insVGGNY, as in SARS-CoV-2) (**Figure 2B**), again highlighting the wider importance of other regions in the RBD and/or Spike in restricted ACE2 usage. Kosugi Y et al., showed a similar result whereby 'filling in' the deletion in the clade Ib Spike, RShTT200 did not confer human ACE2 usage, whereas combinatorial substitutions in other regions of Spike was more successful [49]."*

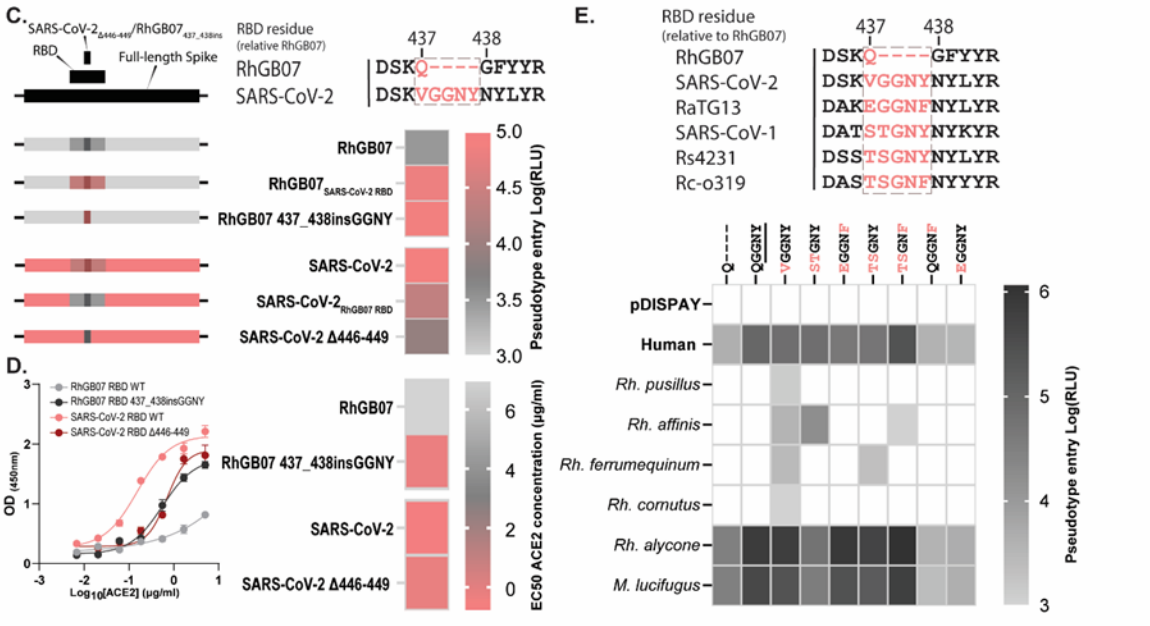

*Legend **Figure 2: (C)** Schematic depicting mutants generated of RhGB07 and SARS-CoV-2 Spikes with either full RBD swaps, or insertions/deletions, respectively. Lentiviral pseudotypes bearing Spikes of RhGB07 or SARS-CoV-2 mutants (RhGB07 wildtype, RhGB07SARS-CoV-2 RBD, RhGB07 437_438insGGNY, SARS-CoV-2 wildtype, SARS-CoV-2RhGB07 RBD, SARS-CoV-2 Δ446-449), were used to infect BHK-21 cells overexpressing human ACE2. A heatmap illustrating entry is shown, representing the mean log RLU of 3 separate experiments. **(D)** ELISA data depicting the binding curves and calculated EC50 values (µg/ml) of recombinant RhGB07, SARS-CoV-2, RhGB07 437_438insGGNY or SARS-CoV-2 Δ446-449 RBDs with recombinant human ACE2. **(E)** Alignment of the Δ4-loop region of RhGB07 with SARS-CoV-2, SARS-CoV-2, RaTG13, Rs4231, and Rc-o319 (RhGB07 numbering), with differences in amino acid residues shown. RhGB07 was mutated to introduce an insertion (437_438insGGNY, underlined) or additional mutations (mutated residues coloured in pink) in the Δ4-loop. A heatmap illustrating entry data is shown for pseudoviruses bearing these mutant Spikes, used to infect BHK-21 cells overexpressing Rh pusillus, Rh. affinis, Rh. ferrumequinum, Rh. cornutus, Rh. alcyone and M. lucifugus ACE2s. Data represents the mean log RLU of 4 separate experiments. A vector only control (pDISPLAY) was included to demonstrate specificity and to set the background signal.*

2. Additional data on RBD binding would be supportive of structure claims. Can you support the RhGB07 RBD vs human & relevant bat ACE2 interactions with purified protein? This would convert qualitative binding and entry phenotypes into interpretable KD/kon/koff values and strengthen mechanistic claims. This could be combined with mutations designed from the structural data of predicted loops and deletions.

We thank the reviewer for this comment. Previously there was no RhGB07 binding data in Supplementary Figure 5. This was because the initial RhGB07 Spike construct we received from our collaborator was un-tagged, so we were unable to perform the flow cytometry experiments and we could not compare binding in flow cytometry vs ELISA. However, in response to your comment we have since produced recombinant RhGB07 RBD and performed ELISAs alongside SARS-CoV-2 RBD, measuring binding against the 3 ACE2 proteins where we detected entry with RhGB07 pseudotypes (human (new **Figure 2D – see point 1 above**), *Rh. Alcyone* and *M. lucifugus*) (**new Supp Figure 5D, below**). Whereas we observed strong binding for these ACE2s with SARS-CoV-2, there was weaker binding throughout with the RhGB07 RBD, quantified by EC50 (µg/ml) in an accompanying heatmap. Correlation of the EC_{50} (**new Supp Figure 5D, below**) and RLU (data from Figure 1D) showed a good relationship between binding and entry.

The main text has been modified to include this data, which now reads (line 454-458): *"Recombinant SARS-CoV-2 and RhGB07 RBDs were also used to assess binding to the three ACE2 species for which RhGB07 entry was observed (human, Rh. Alcyone, M. lucifugus). We identified good binding of SARS-CoV-2 RBD to these ACE2s, which was markedly reduced for RhGB07 RBD, correlating to the pseudotype entry phenotype (**Figure 1D, Supp Figure 5D**)."*

In a previous manuscript in which we were involved and when RhGB07 was first described, we had already showed binding data of RhGB07 RBD with K_D values reported for human ACE2 (K_D 253nM), *Rh. ferrumequinum* ACE2 (K_D 128nM) and *M.lucifugus* ACE2 (K_D 209nM) https://www.nature.com/articles/s41467-023-38717-w#Sec2 , Figure 2B and F).

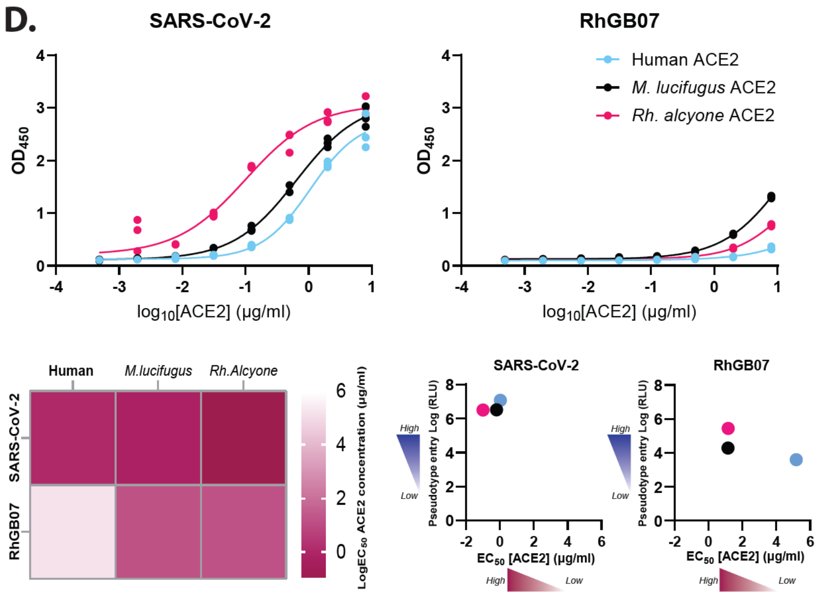

*Legend **Supp Figure 5: (D)** ELISA binding curves of human, M. lucifugus and Rh. alcyone ACE2s with SARS-CoV-2 and RhGB07 RBDs, depicted also as a heatmap with calculated EC_{50} µg/ml. EC_{50} values were also correlated with entry data from Figure 1D. Scale bars indicate direction of increased binding (pink) or entry (blue).*

3. Clarify language in the manuscript. Please replace broad claims ("pan-coronavirus vaccines") with precise phrasing ("pan-sarbecovirus immunogen design informed by

conserved epitopes identified here"), and explicitly note that protection against non-sarbecovirus betacoronaviruses is not supported by the data.

We have scanned the document and cannot find the term "pan-coronavirus" used anywhere. We do use the term "pan-sarbecovirus" (5 times), but we think this is relatively clear, i.e. that we are not making conclusions beyond this sub-genus. We have however, included the precise phrasing included above on lines 912-914 which now reads: *"These data provide promising insights into the regions of Spike that could be targeted to* ***design*** *pan-sarbecovirus* ***immunogens****, or indeed the potential for these monoclonals to be used as pan-sarbecovirus therapeutics,* ***informed by the conserved epitopes identified herein****."*

Reviewer #2 (Xiaoli Xiong):

PBIOLOGY-D-25-03032R1
"Understanding the future risk of bat coronavirus spillover into humans - correlating sarbecovirus receptor usage, host range, and antigenicity."

General Summary

SARS-CoV-1 and SARS-CoV-2, both originating from Rhinolophus bats, underscore the zoonotic risk posed by sarbecoviruses—a genetically diverse group of coronaviruses capable of crossing species barriers. These viruses use the angiotensin-converting enzyme 2 (ACE2) receptor for host cell entry, but their ACE2-binding capacity varies across lineages. Clade I viruses, including SARS-CoV-2 and the BANAL isolates from Laos, display broad or "generalist" ACE2 usage, whereas clades III and V exhibit more restricted, "specialist" patterns. RhGB07, a sarbecovirus identified in the United Kingdom, exemplifies this specialization, binding efficiently to only a limited range of ACE2 receptors. Structural analysis of the RhGB07 Spike protein revealed amino acid motifs responsible for this receptor specificity, offering insight into how host range is determined at the molecular level. The study further examines how receptor usage diversity correlates with antigenic similarity to SARS-CoV-2 and how viral evolution, including that of SARS-CoV-2 variants, affects species tropism. By comparing generalist and specialist profiles across bat and mammalian ACE2 libraries, the research highlights RhGB07 as a useful model for understanding receptor specialization and its implications for sarbecovirus ecology, cross-species transmission, and spillover risk. However, the structural and functional details of RhGB07 receptor specialization remain limited in this study.

Major Comments

This article includes the structural analysis of the RhGB07 Spike protein, and the authors have also attempted to capture the RhGB07 Spike in complex with hACE2 or R. ferrumequinum (Rh.ferr) ACE2. The following points may help strengthen the manuscript:

1. Supplementary Fig. 3: HEK293T cells expressing Rh.ferr ACE2 do not support cell-cell fusion with the RhGB07 Spike protein. Given this, what was the rationale for assembling the

Rh.ferr ACE2-RhGB07 Spike complex? It would be helpful to include quantitative binding data between these ACE2 proteins and RhGB07 S-RBD in Supplementary Fig. 3.

We thank the reviewer for this observation. Alongside the cell-cell fusion data not showing entry of RhGB07 Spike with *Rh.ferrumequinum* ACE2, we also did not see entry in our pseudotype assay in Figure 1D. A previous publication from co-authors of this paper, wherein RhGB07 was first described, also did not show *Rh.ferrumequinum* ACE2 supporting entry of RhGB07 Spike pseudovirus (Figure 2E), but did show binding (Figure 2F) (https://www.nature.com/articles/s41467-023-38717-w#Sec2). The work trying to resolve the RhGB07 Spike structure bound to ACE2 was started by our co-authors (Lau et al.,) before we began working together on this project. The rationale for assembling *Rh. ferrumequinum* ACE2 with RhGB07 was that RhGB07 was isolated from *Rh. hipposideros*, but at the time (and still now), there is no available sequence for *Rh. hipposideros* ACE2. Instead, the host for the closely related RfGB02 (which is *Rh. ferrumequinum*) was tested as this is the most related bat species after the actual host. Of note, the *Rh. ferrumequinum* sequence used in this paper is from the Chinese horseshoe bat, rather than the UK one (no sequence available), which may also account for differences in binding and entry, given also that RfGB02 also does not enter using *Rh. ferrumequinum* in our studies. We have now included binding data for RhGB07 with the ACE2s for which entry was observed in Figure 1D. Purified proteins were produced for RhGB07 RBD and the ACE2s of human, *Rh. alcyone* and *M. lucifugus* ACE2s and were compared with binding to SARS-CoV-2 RBD. Using an ELISA binding assay, we detected strong binding for these ACE2 proteins with SARS-CoV-2 RBD, which was weaker against RhGB07 RBD, quantified also as EC_{50} values (µg/ml) (**Supp Figure 5D**, main text lines 454-458). We were also able to show a good correlation between EC_{50} binding (**Supp Figure 5D**) and RLU entry (data Figure 1D). The main text (lines 454-458) now reads: "*Recombinant SARS-CoV-2 and RhGB07 RBDs were also used to assess binding to the three ACE2 species for which RhGB07 entry was observed (human, Rh. Alcyone, M. lucifugus). We identified good binding of SARS-CoV-2 RBD to these ACE2s, which was markedly reduced for RhGB07 RBD, correlating to the pseudotype entry phenotype (**Figure 1D, Supp Figure 5D**).*"

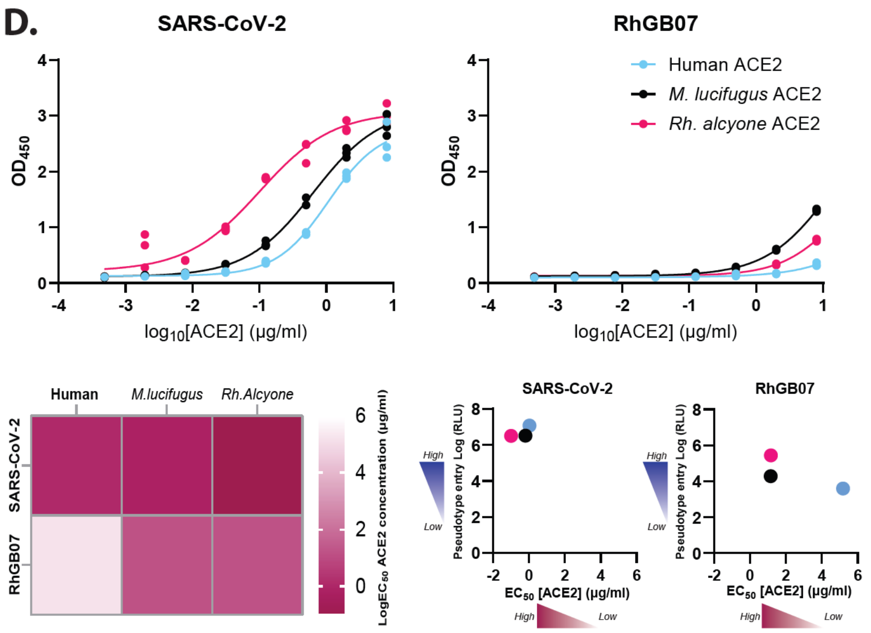

*Legend **Supp Figure 5: (D)** ELISA binding curves of human, M. lucifugus and Rh. alcyone ACE2s with SARS-CoV-2 and RhGB07 RBDs, depicted also as a heatmap with calculated EC_{50} µg/ml. EC_{50} values were also correlated with entry data from Figure 1D. Scale bars indicate direction of increased binding (pink) or entry (blue).*

2. Cell-cell fusion data: Please provide the original cell-cell fusion images in the Supplementary Figures for clarity and reproducibility.

In our initial submission, we did not measure cell-cell fusion by visualising GFP fluorescence as we generally use a quantitative metric of fusion, mearusing *Renilla*-luciferase based luminescence (our assay is dual luciferase and luminescence based). However, we agree with the reviewer that cell-cell fusion images would provide further clarity. We have therefore performed these additional assays (representative images of n=3) to visualise and quantify syncytia formation. We selected representative sarbecovirus Spikes from each clade (clade 1b – SARS-CoV-2, clade 1a – SARS-CoV-1, clade 3 – RhGB07, clade 5 – Rc-o319, and clade 2 – RacCS203) and performed the cell-cell fusion assay, as already described in the methods (line 198). However, in this experiment we co-cultured effector and producer cells in clear flat-bottomed 96-well plates rather than white plates and measured GFP+ cells on an Incucyte S3 live cell imager over time. We captured images at 24 h post co-culture to mirror the time at which luciferase readings were taken. The Incucyte S3 imager also has inbuilt software that allows for quantification of GFP+ cells based on fluorescence intensity and cell area (total integrated intensity metric), which means we can subtract background fluorescence and select cells with the correct morphology (BHK-21 cells are elongated spindle-shaped) for our analysis. As these were separate, additional experiments, we have not included the luciferase data, but we hope the reviewer will agree that both the fluorescence images and quantification of GFP+ cells (**new Supplementary Figure 4, below**) are comparable with the luciferase data already available in Supplementary Figure 3. We also include brightfield images alongside GFP images to show cell confluency and morphology (i.e., we can be confident that the GFP we are seeing is because there is no fusion, rather than there not being enough cells/cell death). We have added text to lines 446-447 to reflect the addition of this data: *"To examine concordance between pseudotype entry and fusion, we repeated the assays in a quantitative cell-cell fusion assay, showing similar trends* ***in quantifiable luciferase signals and GFP+ syncytia formation*** *(Supp Figure 3A,* ***Supp Figure 4****)."*

We have also included a few lines in the methods section to outline how our fluorescence images and analysis were conducted (lines 208-215), which reads: *"To quantify GFP expression, co-cultures were set up of effector and target cells as above into a clear flat-bottomed 96-well plate (Nunc) and imaged 24 h later using the IncuCyte S3 live cell imaging system (Essen BioScience). Four fields of view were taken per well at 10x magnification. Analysis of images generated was performed using the IncuCyte S3 software, setting fluorescence ("masks") and cellular thresholds ("objects") to remove background fluorescence. The total sum of objects with fluorescent masks was then calculated using the total integrated intensity metric, expressed as green count units (GCU) µm2."*

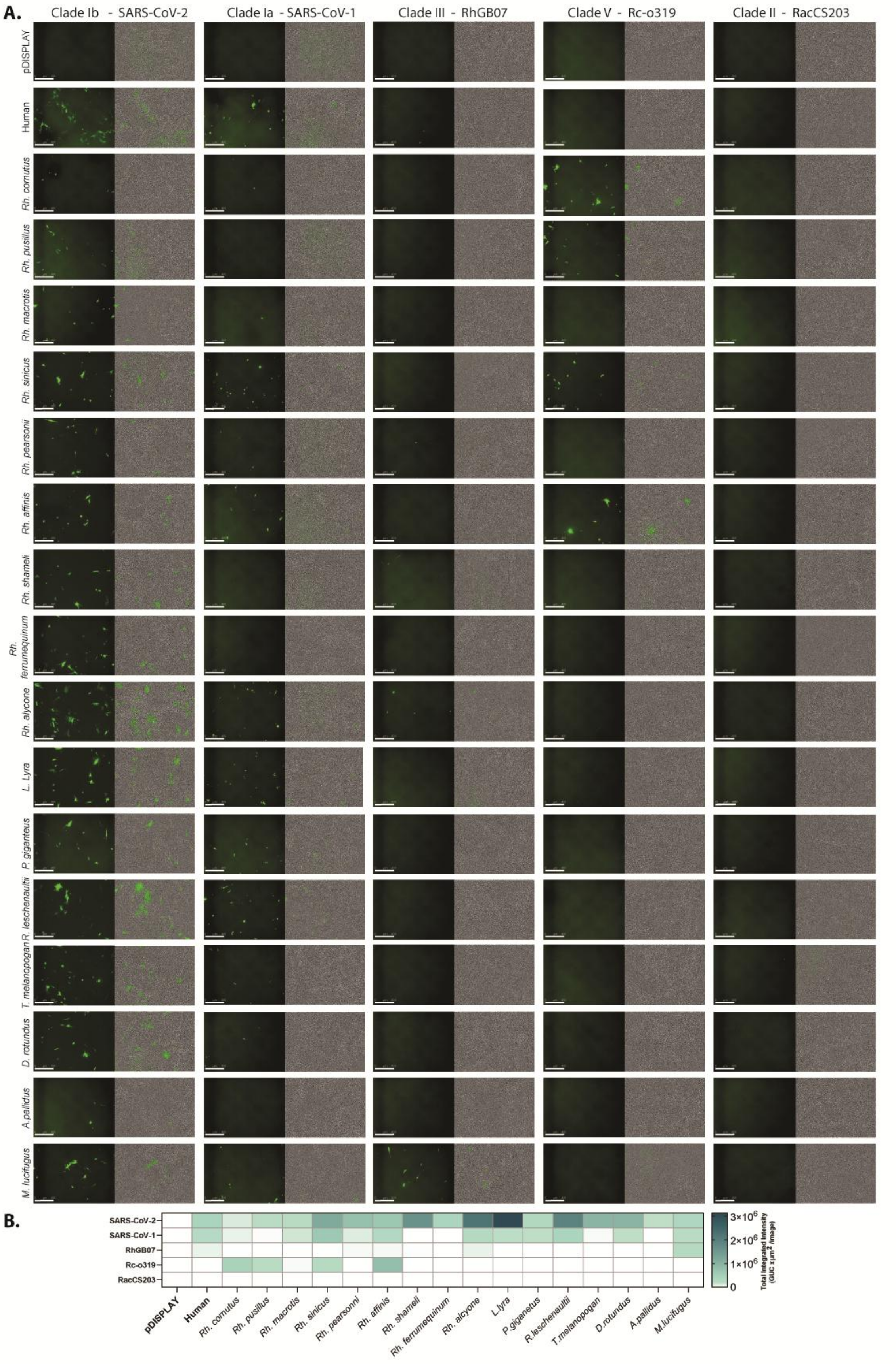

*Legend **Supplementary Figure 4: GFP+ syncytia formation of bat sarbecovirus entry by cell-cell fusion. (A)** GFP and brightfield images of cell-cell fusion of representative bat sarbecovirus Spikes (transfected into HEK293T effector cells stably expressing rLuc-GFP 1-7) with bat ACE2s*

(transfected into BHK-21 cells expressing rLuc-GFP 8-11) 24 h after co-culture. Four fields of view were taken per well at 10x magnification. Scale bar is shown, 400µm. ***(B)*** *Analysis of images generated was performed using the IncuCyte S2 software, calculating the total sum of object with fluorescence above the background threshold, measured as total integrated intensity, and expressed as green count units (GCU) µm^2 and shown in a heatmap.*

3. The reported C3-symmetric RBD-down structure of the RhGB07 Spike represents the only conformation obtained. Were any small-molecule densities—such as lipids or biliverdin—observed in the RBD or NTD domains?

We thank the reviewer for this comment. We note that we have inspected the density for the presence of such lipids (ie. Toelzer, C et al, 2020 Free fatty acid binding pocket in the locked structure of SARS-CoV-2 spike protein - PMC) or biliverdin (ie Wang, J. et al, 2025 SARS-related coronavirus S-protein structures reveal synergistic RBM interactions underpinning high-affinity human ACE2 binding | Science Advances). We did not mention it in the first version, but it is now explicitly mentioned it in the text (lines 487-490) as no such density was observed in either RBD or NTD domains; the text reads: *"It was also noted that the closed-state PRD-0038 RBD structure resembled SARS-CoV-2 RBD with a fatty acid, linoleic acid, bound [14]. An examination of the RhGB07 RBD revealed no observable density (Supp Figure 7C) [40]. Similarly, the NTD, that in some clade 3 binds biliverdin, also did not reveal any density (**Supp Figure 7C**) [41]."* We have added a new **Supplemental Figure panel 7C (below)** to illustrate this.

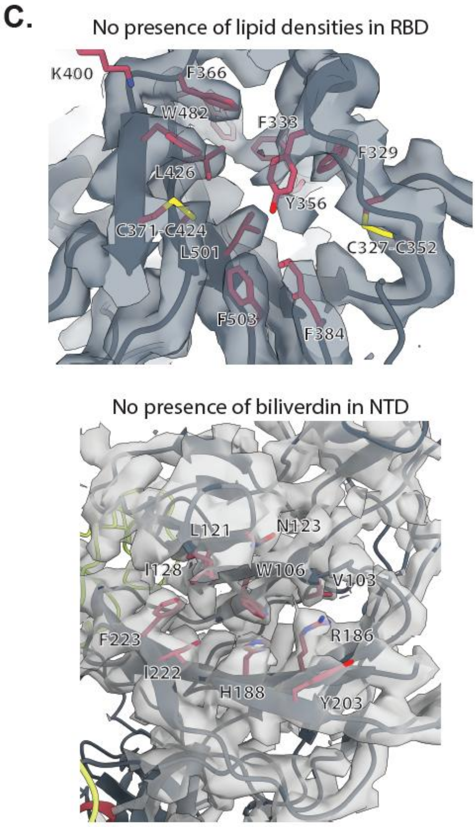

Legend ***Supplementary Figure 7: (C)*** *RBD (top) and NTD (bottom) of RhGB07 cartoon depicted with the cryo-EM density contoured in this region. Residues comprising a putative fatty acid binding pocket as seen in SARS-CoV-2 RBD [40] or a putative biliverdin binding pocket as seen in other coronavirus NTD [41] are highlighted. No density was present in either of these pockets corresponding to a fatty acid or biliverdin, respectively.*

4. If only a single RhGB07 Spike structure was obtained, then when comparing RBD conformations in Fig. 2B, the locked SARS-CoV-2 and SARS-CoV-1 RBD structures would be more appropriate controls than the SARS-CoV-2 RBD-ACE2 complex (PDB: 6M0J).

Thank you for this comment. We have now updated Figure 2B to use the locked RBD structures of SARS-CoV-1 (5X58) and SARS-CoV-2 (7DF3) (see below), because as suggested these are the better comparisons. Although there are slight differences, the insertions and deletions clearly still influence the residues that are putatively ACE2-interacting residues.

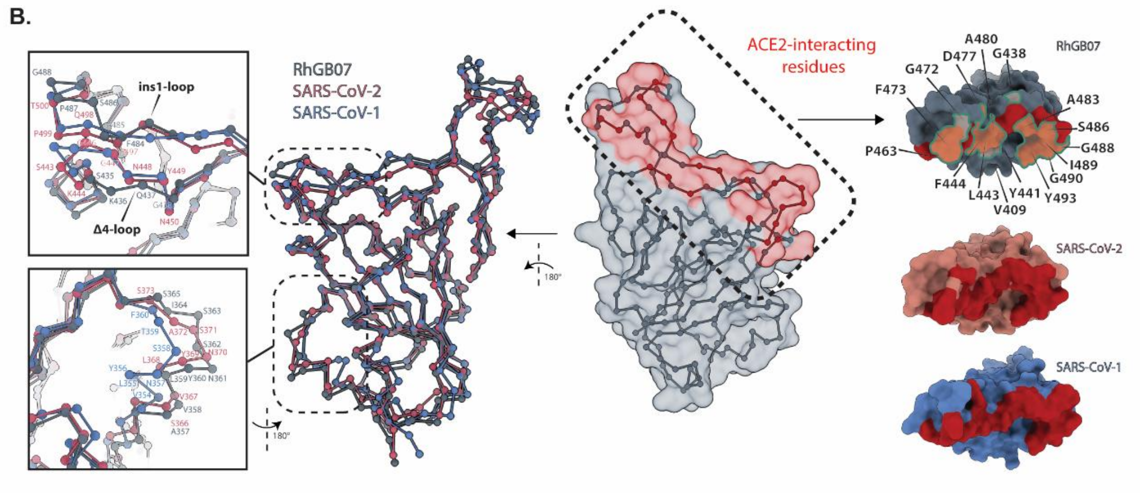

Legend ***Figure 2: (B)*** *The C-alpha trace of the RBD of RhGB07 rendered as balls-and-sticks superimposed with the RBD of SARS-CoV-1 (PDB 7DF3) and SARS-CoV-2 (PDB 5X5B). The insets show a zoomed-in view of two regions of the RBD that show the greatest changes in the position of the C-alpha of the RBD. The region containing the ins1-loop and delta4-loop coincides with putative ACE2-interacting residues based on mapping the equivalent residues of SARS-CoV-1 and SARS-CoV-2. The ACE2 interacting residues were determined by superimposing SARS-CoV-1 and RhGB07 RBD onto the SARS-CoV-2 RBD – ACE2 (PDB: 6M0J) structure and identifying interacting residues. Highlighted residues of RhGB07 show the putative interacting residues that differ in identity to SARS-CoV-2.*

5. Supplementary Fig. 5: The FSC curves do not drop to zero. Please check the Nyquist limit or consider removing duplicate particles.

We thank the reviewer for the observation and for paying close attention to these details. The particles were Fourier-cropped during particle extraction from an original 500-pixel box (0.83 Å/pixel) to a 300-pixel box, resulting in an effective pixel size of 1.38 Å/pixel. As a result, the FSC curve is truncated at the Nyquist limit of 2.77 Å (2 x pixel size). Our current resolution is just below the limit, 2.97 Å. As the signal in our data remains significant up to this sampling limit, the FSC does not fully decay to zero.

As the current map density is sufficient for accurate atomic modelling, we maintained these binned particles during processing for computational efficiency. We have now mentioned this more explicitly in the methods and in our workflow (lines 257-263), which now reads: ***"The 2D classes already show a trimeric spike resembling a closed conformation****. These particles were sufficient to produce an ab-initio model and can be refined to 3.66 Å with C3 symmetry. To increase the resolution and quality of the map these 2D classes were used once again for template picking which led to 2 234 763 particles. After several rounds of extracting, cropping* ***(Box size 500 pixel, Crop size 300 pixel)*** *and classification only 21 821 particles remained* ***(3.09 Å, C3 symmetry)*** *and were subjected to heterogenous refinement* ***(C1 symmetry)*** *with 4 classes. The 3 best classes in terms of resolution were used (17 685 particles) for a final round of NU-Refinement leading to a map of 2.97 Å (FSC 0.143, C3 symmetry). This map showed good local*

resolution at the RBD without necessitating for local refinement for this domain. ***Due to cropping during particle extraction, the FSC curve is truncated at the Nyquist limit of 2.77 Å and does not fully decay to zero****.”*

This is also seen in the updated **Supplementary Figure 6.** We do not believe however that reprocessing would lead to any significant changes in the interpretation of either the density map or the model that has been built within it.

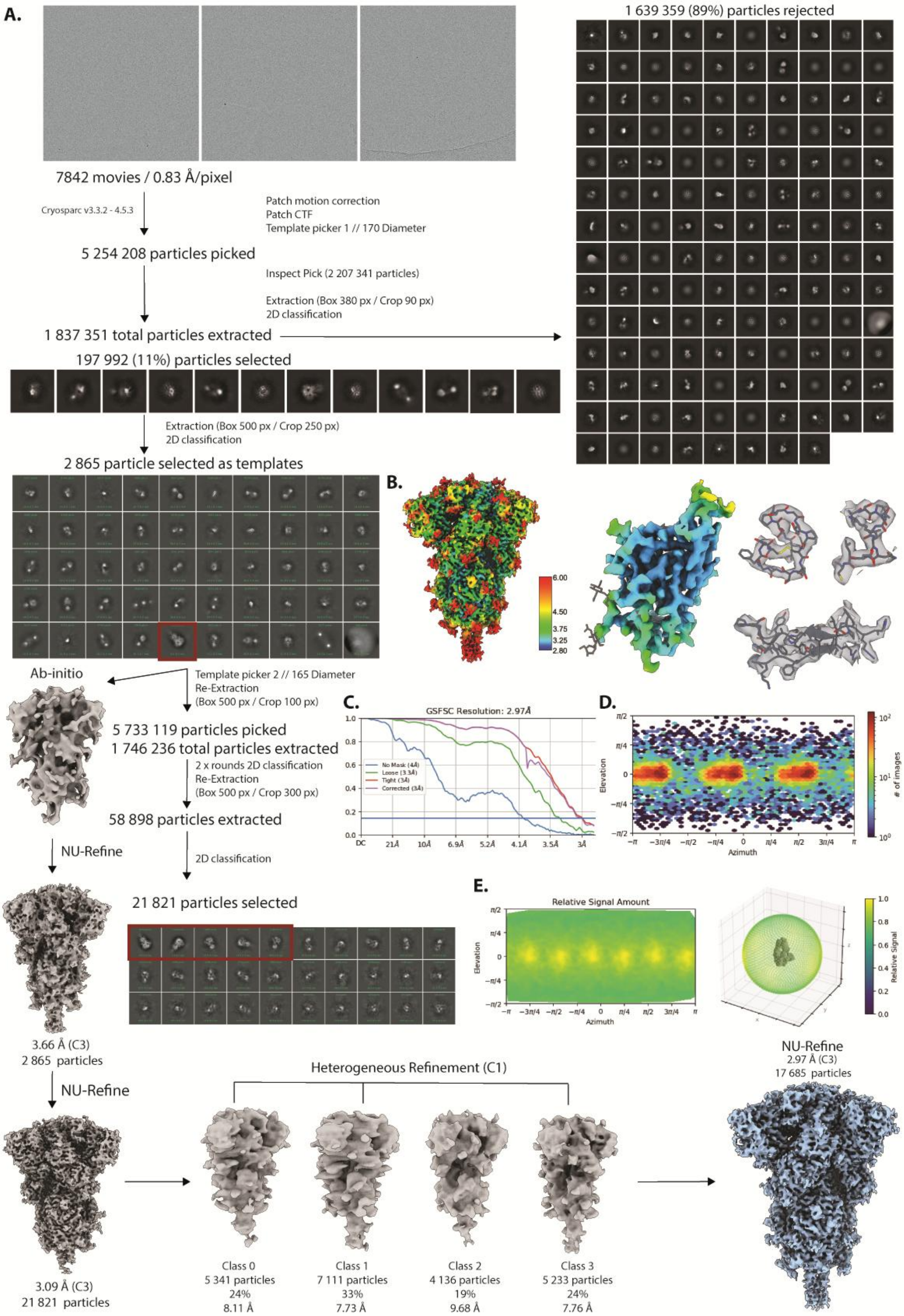

Legend ***Supplementary Figure 6: Cryo-EM Processing Workflow (A)*** *Processing workflow of for the determination of the RhGB07 full-length trimeric spike.* ***(B)*** *Cryo-EM density coloured by local-resolution of the full-length trimer and of the isolated RBD. Selected regions represented with*

their cryo-EM density is also shown. ***(C)*** *FSC curve showing a determined resolution for the full-length spike of 2.97 Å* ***(D)*** *Viewing direction distribution of particles used in the reconstruction.* ***(E)*** *Relative Signal vs Viewing direction plot of particles. Cryo-EM data collection, refinement and validation statistics can be sound in* ***S3 Table****.*

6. Regarding the monoclonal antibodies isolated from SARS-CoV-2 breakthrough infections: Why does the group 2 antibody VA14_26 broadly bind BANALs, WIV1, Rs4231, and Rc-o319, but not RhGB07 or RfGB02? Given that Rc-o319 has a nine-amino acid deletion in its RBD, where exactly does this antibody bind? Interestingly, mAb VA14_26 shows no binding to RhGB07 in the ELISA assay (Fig. 2E), yet it neutralizes RhGB07 pseudoviruses (Fig. 7B, second panel). This discrepancy should be clarified.

Thank you for these comments. The binding residues of VA14_26 to WIV-1 RBD have been described in a sister paper submitted alongside this manuscript - see Figure 3D (Mechanisms for cross-neutralisation of diverse bat sarbecoviruses | bioRxiv). Of note, the binding residues of VA14_26 do not interact with regions where there are deletions in Rc-o319/RhGB07, so it is unlikely the deletions in these RBDs plays a role in the lack of binding observed for RhGB07. Of these binding residues, differences in amino acid residues at position 366 (WIV-1 numbering) appear to be most important. This changes from a K to Q in Rc-o319, which would result in less positive, weaker interactions, evidenced by the reduced binding observed in Fig 6E, and from a K to an E in RhGB07 which would be repulsive (positive to negative charge) and ablate binding. To help explain this in more detail we have now added structural data of VA14_26 interacting with WIV-1, SARS-CoV-2, Rc-o319 and RhGB07 to illustrate this, highlighting these deterministic residues (**new Supp Figure 12A, see below**) and have added a line to the main text (lines 712-715) to address this: "*The lack of binding of VA14_26 with RhGB07 RBD is likely due to differences in amino acid residues present at the antibody-RBD interface that result in weaker, repulsive interactions (K370E and R400K in RhGB07 compared to SARS-CoV-2, WIV-1 and Rc-o319) (**Supp Figure 12A**).*"

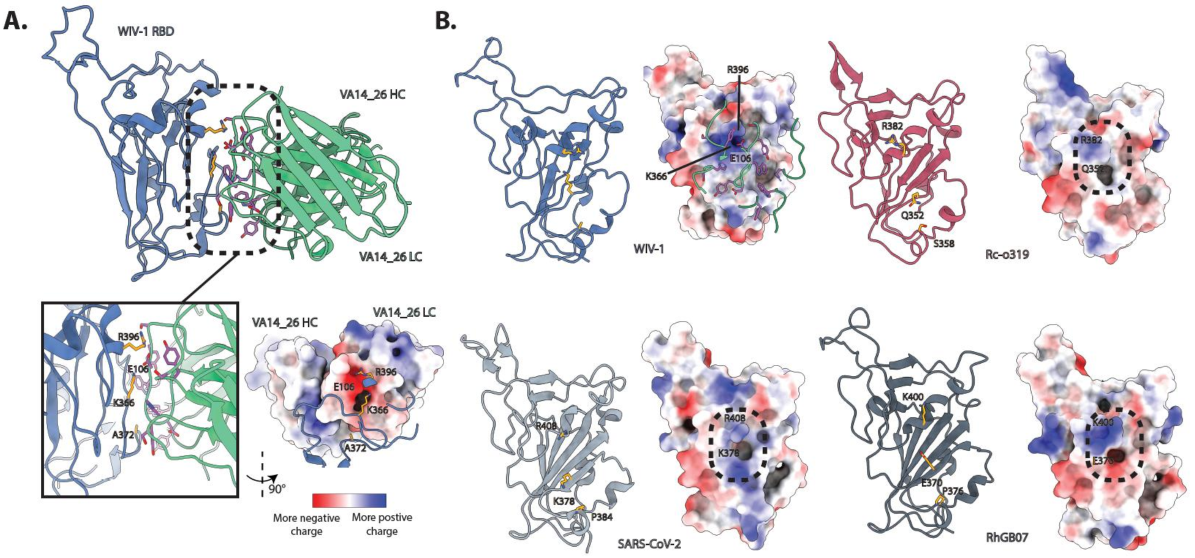

Legend ***Supplementary Figure 12:*** *The* ***(A)*** *WIV-1-RBD (blue) / VA14_26 complex (green) (WIV-1 PDB 8WLZ, [48]) is shown in cartoon representation. Only the VH and VL domains of the Fab of VA14_26 are shown for clarity. Residues of interest on the WIV-1 RBD that make contacts with VA14_26, R396, K366, A372 are coloured in orange. Contact residues on VA14_26 are coloured in purple. The inset shows a zoomed in highlight of this interface. A surface representation of*

VA14_26 coloured by electrostatic potential highlights that the WIV-1 RBD residues R396 and K366 that are positively charged bind and localize in a highly negative patch of the Fab demonstrating that VA14_26 interactions are mediated by charge attraction.

With regards to the binding and neutralisation data for VA14_26 with RhGB07 shown in Figure 7. Our binding data shows no binding by ELISA – see Figure 6E. In Figure 7, the higher EC50 value (for binding, **Figure 7A**) or higher IC50 value (for neutralisation, **Figure 7B**) actually indicates more antibody is required, not less. In this instance the high EC50 and IC50 values recorded for VA14_26 against RhGB07 depict the upper limit of our assays, which corresponds to no binding/neutralisation. We admit this is a little misleading as the graphs are inverted relative to the ELISA data in Figure 6, but this is standard for EC50s and IC50s as opposed to OD. To make this clearer we have now added scale bars to the side of the graphs in **Figures 7A and 7B (below)** to show the directionality of binding or neutralisation and have amended the figure legend to make this clearer. We apologise for the confusion.

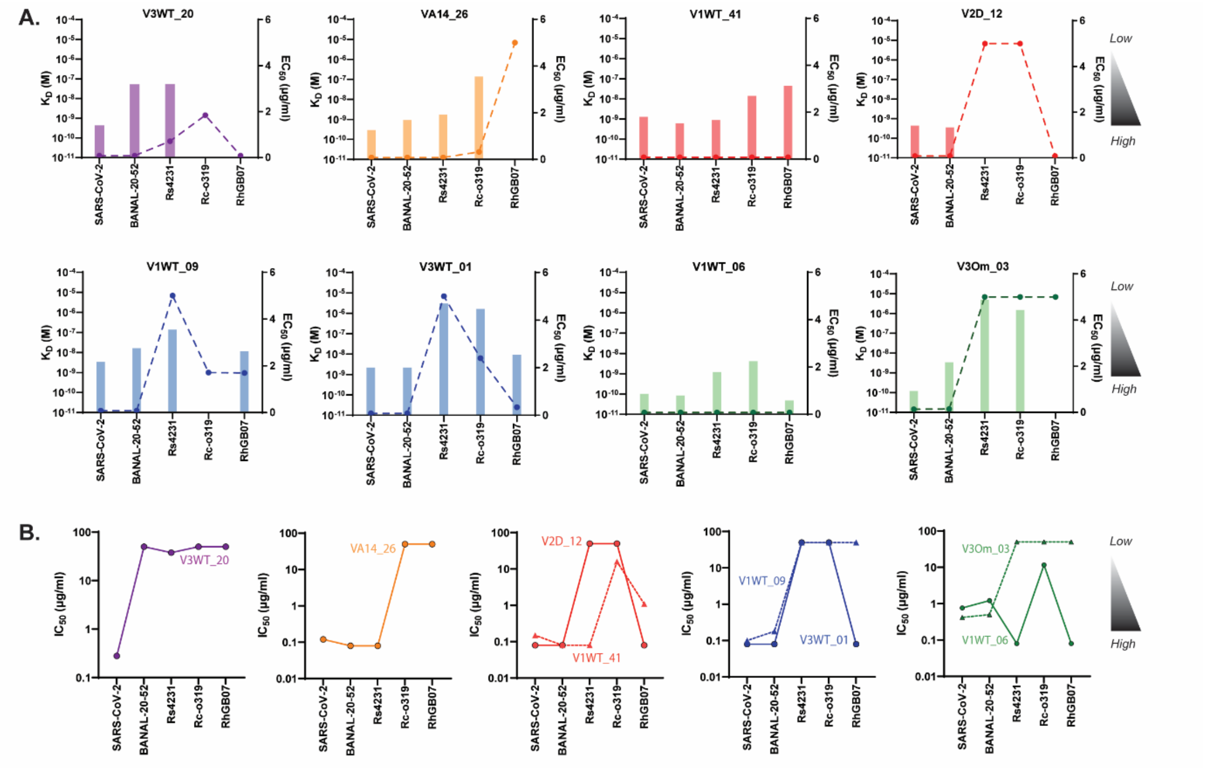

Legend **Figure 7:** ***(A)*** *Dissociation constant (K_D (M), 2-4 replicates ± sd) of mAb binding kinetics calculated by SPR (bars) and ELISA EC_{50} (µg/ml, 2 replicates ± sd) values (broken lines with filled symbols) for a subset of mAbs and sarbecovirus RBDs. For K_D, no bar indicates no binding by SPR. For EC_{50}, lower limit of detection: <0.08µg/ml, upper limit of detection: >5µg/ml.* ***(B)*** *Neutralisation (IC_{50}) titres shown for each mAb against bat sarbecovirus pseudotyped viruses (µg/ml), with limits of detection indicated (lower limit: <0.08µg/ml; upper limit: >50µg/ml for IC_{50}). HEK293T cells stably expressing human ACE2 were used for all neutralisation assays. apart from RhGB07 and Rc-o319, where HEK293T cells overexpressing Rh. alcyone or Rh. cornutus were used, respectively. Scale bars (right) represent direction of increased binding or neutralisation.*

7. Lines 1052-1053: In the description, "The Cα trace of the RBD of RhGB07 rendered as balls and sticks superimposed with the RBD of PRD-0038 apo or PRD-0039 bound to R. alcyone ACE2," the label "PRD-0039" should be corrected to "PRD-0038." Additional structural alignments of clade 3 RBDs would further strengthen the analysis.

Thank you for noting the typo - we have also corrected the typo on line 1064 (Supp Fig 7 legend). With regards to other Clade 3 RBDs, we have now added an additional Supplementary panel (new **Supp figure 7A, below**) which shows additional Clade 3 Spike structures namely, BtKY72, Khosta-1 and Khosta-2 in addition to PRD-0038. This additional data shows similarities to the observed differences with PRD-0038, principally the insertion of residues in the loop that makes direct contact with ACE2 in other RBDs, as indicated. The main text has been edited to include this new panel: *"Comparison with other Clade III RBD apo-structures, PRD-0038, Khosta-1, Khosta-2 and BtKY72 revealed high similarity between the C-alpha traces (**Supp Figures 7A and B**). However, regions of differences were noted that coincide with putative ACE2-interacting residues based, particularly in the ins1-loop and Δ4-loop, which could impact ACE2 usage and receptor tropism."*

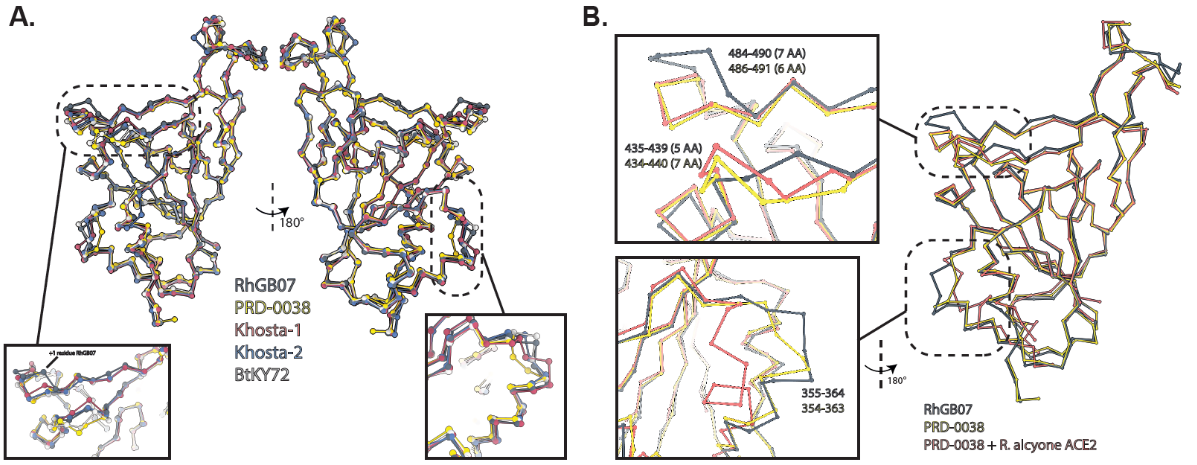

*Legend **Supplementary Figure 7: (A)** The C- α trace of the RBD of RhGB07 rendered as balls-and-sticks superimposed with the RBD apostructures of PRD-0038 (PDB 8U29), Khosta-1 (PDB 9J6G), Khosta-2 (PDB: 9J5U) and BtKY72 (PDB 8ZY0). The insets highlights regions of interest of the RBD that are either involved in binding of ACE2 or have presented structural changes across clades. **(B)** The C-α trace of the RBD of RhGB07 rendered as balls-and-sticks superimposed with the RBD of PRD-0038 apo or PRD-0038 bound to R. Alcyone ACE2 (PDB: 8U0T). The insets show a zoomed-in view of two regions of the RBD that show the greatest changes in the position of the C-α of the RBD. The region containing the ins1-loop and Δ4-loop coincides with putative ACE2-interacting residues based on mapping the equivalent residues.*

8. The authors have missed recent articles analyzing Rc-o319 specificity (https://doi.org/10.1016/j.celrep.2025.116220;https://doi.org/10.1101/2025.09.17.676762), Rc-o319 has a very different RBM as shown by the new structures. The authors should discuss these new results and cross-reference the Rc-o319 binding data in the two articles. In addition, the authors missed a recent article analyzing how non-human ACE2 binder can acquire high-affinity human ACE2 binding (DOI: 10.1126/sciadv.adr8772). This article shows that high-affinity ACE2 binding needs optimal interactions across 4 different ACE2 binding interfaces within the RBM for specific ACE2 molecules. If optimal interactions are found in 3-4 interfaces, it will result in high-affinity binding. Whereas optimal interactions are found in ~2 interfaces, it will result in weak to moderate ACE2 binding. For example, clade 3 RBD BtKY72, with a small loop deletion, can bind R.a-ACE2 at a moderate affinity due to good interactions in 3 interfaces, even if the small loop interface is completely missing. For clade 1, although without deletions in the RBM, interactions in the 4 interfaces for specific ACE2

may not be optimal, therefore could result in varied affinity. The authors should discuss Sarbecovirus ACE2 binding specificity in light of these findings in previous works.

We thank the reviewer for drawing our attention to publications relevant to our work that we missed. We have now changed the discussion section to include the works by Kosugi Y et al., (2025) (Molecular basis of sarbecovirus evolution and receptor tropism in natural hosts, potential intermediate hosts, and humans: Cell Reports) and the pre-print by Wang et al., (2026) (Structural Basis for Sarbecovirus Rc-o319 Spike Adaptation to Rhinolophus cornutus Bat ACE2 and Constraints on Switching to Human ACE2 | bioRxiv) to include Rc-o319 binding data (lines 813-819) which reads: *"Other clade V sarbecoviruses Rc-os20, Rc-kw8 and Rc-mk2 have displayed similar restrictions, using primarily the cognate Rh. cornutus ACE2 receptor [49]. The Rc-o319 RBD contains a 10 amino acid deletion in region 2 (481-499) compared to the SARS-CoV-2 RBD, which likely contributes to this specialist receptor usage profile [50]. This has been confirmed by recent structural data which highlights structural incompatibility between the Rc-o319 RBD and human ACE2, which require combinatorial mutations in specific RBM regions to gain medium-to-high affinity for human ACE2 [53]."*

We had already referenced work by Wang et al., (2025) (SARS-related coronavirus S-protein structures reveal synergistic RBM interactions underpinning high-affinity human ACE2 binding | Science Advances) to compare RhGB07 with other clade III sarbecoviruses, but have expanded this to also mention the article in relation to ACE2 affinity. We have included in the discussion the significance of the 4 described binding interfaces (lines 836 – 847) which reads: *"Generally speaking, variation at the ACE2-binding interface likely impacts the affinity of different bat sarbecovirus Spikes for ACE2, as suggested in recent studies by Kosugi Y et al., and Wang J et al., which describe four regions in the Spike RBM that form contacts with ACE2, resulting in high affinity binding if 3-4 of these interfaces are intact, weak-to-moderate binding if 2 interfaces are intact, and little-to-no binding if only 1 interface is present [41, 49]. This was shown for the clade Ib Spike RShSTT200 [49], the clade III Spikes BtKY72 and BM48-31 [41], and the clade V Spike Rc-o319 [53]. The optimal binding interfaces may also differ between clades, for example, the clade III RBD BtKY72 is able to bind Rh. affinis ACE2 with high affinity due to the presence of 3 of these binding interfaces, despite the deletion at positions 445-449 (SARS-CoV-2 numbering) [41]; conversely, for the clade Ib virus RShSTT200, which also contains the 445-449 deletion, interactions with the other three interfaces still result in weak binding with Rh. affinis ACE2 [49]."*

Furthermore, we have also referenced the work by Kosugi Y et al., (2025) in our discussion to further discuss:

(i) Differences in host range of clade Ib sarbecovirus RShSTT200 (line 776-779), which reads: *"Similarly, the clade Ib sarbecovirus RShSTT200 has also been shown to have restricted host range, including to human ACE2, which the authors attributed to amino acid differences in Spike, relative to SARS-CoV-2, e.g., P486F, T346R, D487N, Y496G, or deletions at positions 445-445 and 448-489 (SARS-CoV-2 numbering) [49]."*

(ii) Restrictions of other clade V sarbecoviruses similar to what we observe for Rc-o319 (lines 813-814), which reads: *"Other clade V sarbecoviruses Rc-os20, Rc-kw8 and Rc-mk2 have displayed similar restrictions, using primarily the cognate Rh. cornutus ACE2 receptor [49]."*

(iii) The impact of deletions and substitutions in clade Ib Spike that confer human ACE2 usage (line 808-811), which reads: *"Kosugi Y et al., showed a similar result whereby 'filling in' the deletion in the clade Ib Spike, RShTT200 did not confer human ACE2 usage, whereas combinatorial substitutions in other regions of Spike was more successful [49]."*

Minor Typographical and Editorial Corrections

*** Line 56: "cluster" → "clusters" (plural verb agreement; "analyses" is plural).**
*** Line 69: Add a comma after "etc." or use "and others," e.g., "(BANAL-20-52, BANAL-20-103, etc.), raising intriguing questions..."**
*** Line 729: If the RBD of SARS-CoV-2 is used as the control, it should indicate that Rc-o319 lacks 9 amino acids in its RBD.**

The minor typographical corrections as listed above have been corrected for lines 56 and 69. For line 748 (now line 814-816), this has been amended and now reads *"The Rc-o319 RBD contains a 10 amino acid deletion in region 2 (481-499) compared to the SARS-CoV-2 RBD, which likely contributes to this specialist receptor usage profile."*

Reviewer #3:

The manuscript by Thakur et al. addresses receptor tropism across sarbecoviruses and the antigenic landscape of SARS-CoV-2 immunity. The paper is clearly written and the central message is well conveyed. However, much of the background on ACE2 usage and species-specific receptor compatibility among SARS-CoV-2 and bat sarbecoviruses has been reported previously (e.g., Emerging Infectious Diseases 31:8 (2024), Nat Commun 13:3868 (2022), Sig Transduct Target Ther 5:282 (2020), Cell 184:4203 (2021), Nature 609:597 (2022)). Similarly, cross-reactive neutralization by SARS-CoV-2-elicited antibodies across sarbecoviruses has been documented (Nat Microbiol 7:1867 (2022), Commun Biol (2025), Cell (2024), Nature 597:103 (2021), Sci Transl Med (2024)). In my view, the principal novelty here lies in the structural resolution of RhGB07 Spike and the downstream functional characterization: quantitative binding to diverse ACE2 orthologs and a defined neutralization-escape profile for RhGB07.

To strengthen the manuscript contribution, I have following recommendation:

1. Resolve the structure of RhGB07 spike with Rh. alcyone ACE2.

Thank you for your suggestion. Recent studies by Lee J et al., 2025 (Broad receptor tropism and immunogenicity of a clade 3 sarbecovirus: Cell Host & Microbe) and Wang J et al., 2025 (SARS-related coronavirus S-protein structures reveal synergistic RBM interactions underpinning high...) also determine cryo-EM structures for other clade III bat sarbecoviruses Spikes to similar resolutions (PRD-0038: 2.9Å, BtKY72: 3.0Å, BM48-31:3.1Å, YN2013: 2.5Å, RmYN02: 3.8 Å, GX2013: 3.3 Å, HeB2013: 3.2 Å) as we describe for RhGB07 (2.97Å). Importantly, although these authors were able to determine a structure of full-length Spike, all their ACE2 complexes were solely with the RBD, not the whole Spike protein. To mitigate this and aid comparison with these new data we have added additional C-alpha traces of different clade III bat sarbecovirus RBDs (PRD3008 apo, Khosta-1, Khosta-2, BtKY72) overlayed with RhGB07, which appear highly similar and also show the RBD in a closed state in context of the full-length Spike **(new Supp Figure 7A, below).** Therefore, we do not believe resolution of RhGB07 Spike with *Rh. alcyone* ACE2 will be possible without extensive time and effort and will not add significantly to our current understanding. However, our structure-targeted mutation of the deterministic residues (see **new**

Figure 2C-2E, below) does aim to address this, and we hope this mitigates the reviewer's concerns.

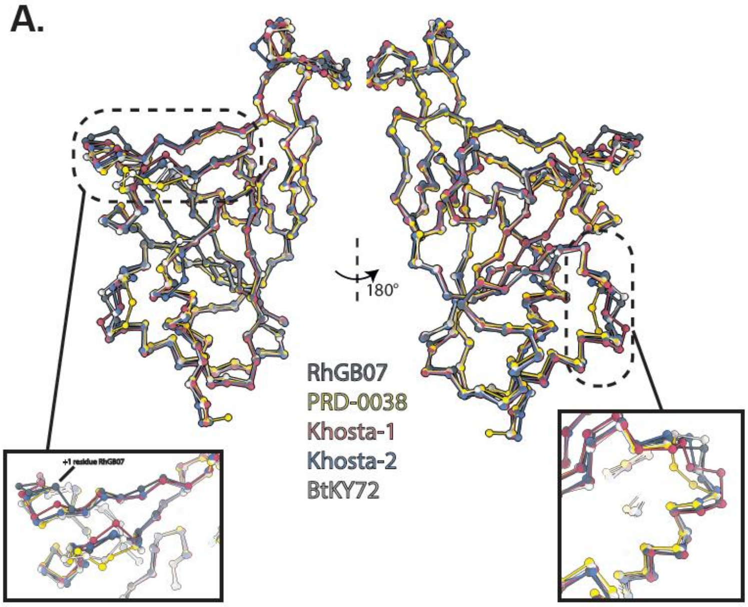

Legend ***Supplementary Figure 7: (A)*** *The C- α trace of the RBD of RhGB07 rendered as balls-and-sticks superimposed with the RBD apostructures of PRD-0038 (PDB 8U29), Khosta-1 (PDB 9J6G), Khosta-2 (PDB: 9J5U) and BtKY72 (PDB 8ZY0). The insets highlights regions of interest of the RBD that are either involved in binding of ACE2 or have presented structural changes across clades.*

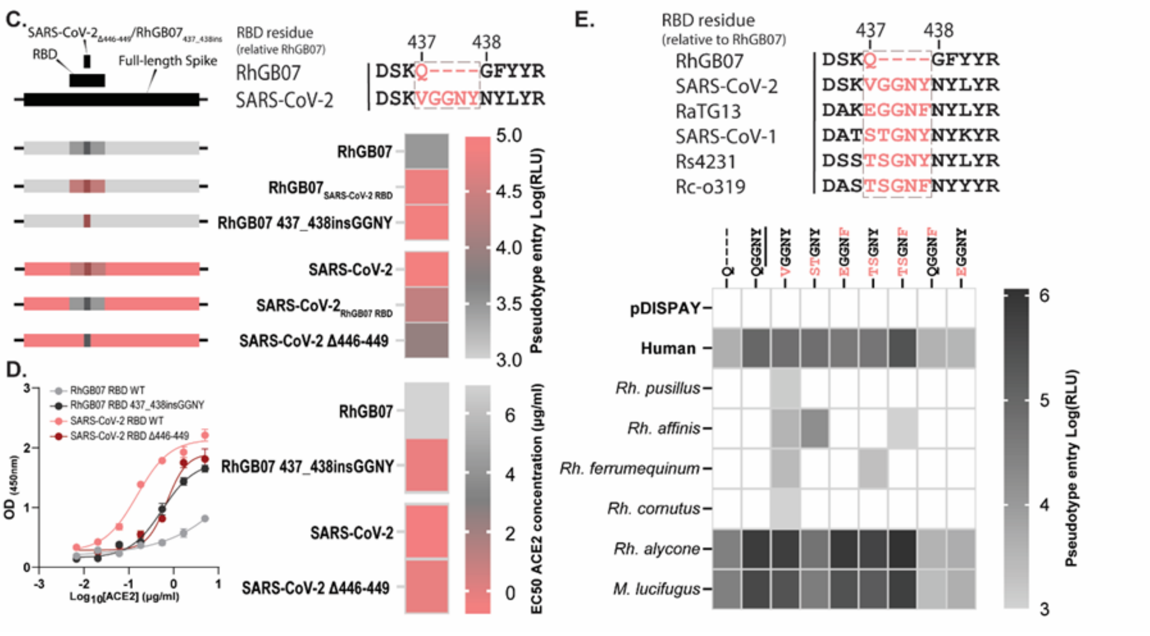

Legend ***Figure 2: (C)*** *Schematic depicting mutants generated of RhGB07 and SARS-CoV-2 Spikes with either full RBD swaps, or insertions/deletions, respectively. Lentiviral pseudotypes bearing Spikes of RhGB07 or SARS-CoV-2 mutants (RhGB07 wildtype, RhGB07SARS-CoV-2 RBD, RhGB07 437_438insGGNY, SARS-CoV-2 wildtype, SARS-CoV-2RhGB07 RBD, SARS-CoV-2 Δ446-449), were used to infect BHK-21 cells overexpressing human ACE2. A heatmap illustrating entry is shown, representing the mean log RLU of 3 separate experiments.* ***(D)*** *ELISA data depicting the binding curves and calculated EC50 values (μg/ml) of recombinant RhGB07, SARS-CoV-2, RhGB07 437_438insGGNY or SARS-CoV-2 Δ446-449 RBDs with recombinant human ACE2.* ***(E)*** *Alignment of the Δ4-loop region of RhGB07 with SARS-CoV-2, SARS-CoV-2, RaTG13, Rs4231, and*

Rc-o319 (RhGB07 numbering), with differences in amino acid residues shown. RhGB07 was mutated to introduce an insertion (437_438insGGNY, underlined) or additional mutations (mutated residues coloured in pink) in the Δ4-loop. A heatmap illustrating entry data is shown for pseudoviruses bearing these mutant Spikes, used to infect BHK-21 cells overexpressing Rh pusillus, Rh. affinis, Rh. ferrumequinum, Rh. cornutus, Rh. alcyone and M. lucifugus ACE2s. Data represents the mean log RLU of 4 separate experiments. A vector only control (pDISPLAY) was included to demonstrate specificity and to set the background signal.

2. What is the binding affinity of RhGB07 RBD for Rh. alcyone ACE2, compared to ACE2 from different species?

Again, thank you for the suggestion. To address this question, we produced recombinant proteins representing the RhGB07 RBD and performed ELISAs to examine binding with purified human, *Rh. alcyone* and *M. lucifugus* ACE2s. In these assays we observed weak binding of RhGB07 RBD with these ACE2s, which was however, stronger with Rh. alcyone ACE2 and M. lucifugus ACE2 than with human ACE2 (**new Supp Figure 5D**). In comparison, binding of these ACE2s by recombinant SARS-CoV-2 RBD was much greater, correlating to our entry data. We also calculated the EC50 for these binding interactions, which also showed good correlation with the entry data shown in Figure 1D (**Supp Figure 5D**). The main text has also now been modified to include these results (lines 454-458), which reads: *"Recombinant SARS-CoV-2 and RhGB07 RBDs were also used to assess binding to the three ACE2 species for which RhGB07 entry was observed (human, Rh. Alcyone, M. lucifugus). We identified good binding of SARS-CoV-2 RBD to these ACE2s, which was markedly reduced for RhGB07 RBD, correlating to the pseudotype entry phenotypes (Figure 1D, Supp Figure 5D)."*

Of note, in the manuscript where RhGB07 was first described (Tan et al., 2023), we already recorded binding data for RhGB07 RBD with K_D values reported for human ACE2 (K_D 253nM), *Rh. ferrumequinum* ACE2 (K_D 128nM) and *M.lucifugus* ACE2 (K_D 209nM) https://www.nature.com/articles/s41467-023-38717-w#Sec2 , Figure 2B and F). This evidence of weak binding, e.g. when compared to reported SARS2 -human ACE2 affinities, is consistent with our ELISA data.

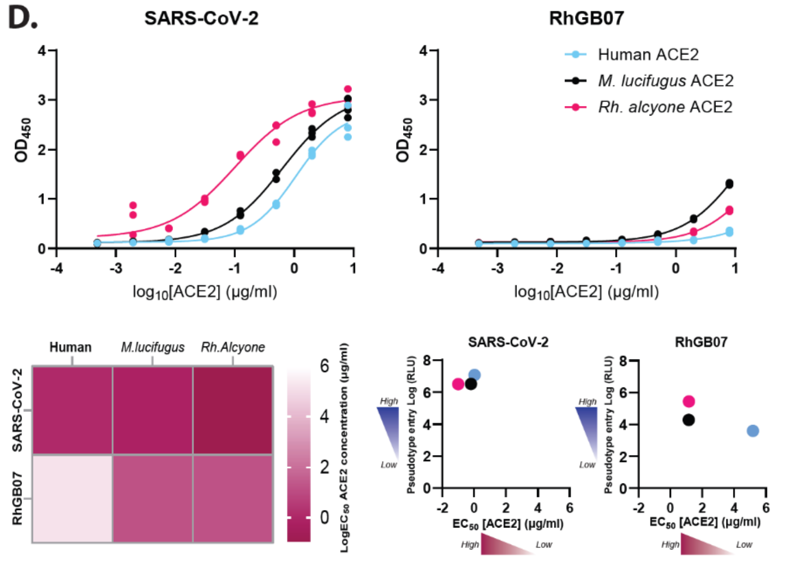

*Legend **Supp Figure 5: (D)** ELISA binding curves of human, M. lucifugus and Rh. alcyone ACE2s with SARS-CoV-2 and RhGB07 RBDs, depicted also as a heatmap with calculated EC_{50} μg/ml. EC_{50} values were also correlated with entry data from Figure 1D. Scale bars indicate direction of increased binding (pink) or entry (blue).*

3. How did the authors normalise the entry data since the pseudovirus packaging efficiency varies between sarbecovirus spikes, as shown in Supplementary Fig. 2b?

We thank the reviewer for this comment and concur that pseudovirus packaging efficiency may vary between sarbecovirus Spikes. The antibody used in the middle panel (anti-Spike) for Supplementary Figure 2B (now Supp Fig 2C) is specific for SARS-CoV-2 and therefore may not have broad specificity for other sarbecovirus Spikes, particularly in elucidating Spike processing. For example, we can see that the S band for SARS-CoV-2 has a lower signal because most of the Spike is processed to S2, whereas for most of the others, S2 processing is less prominent. Therefore, the anti-S expression should be considered qualitative at best. In general, the Flag tag expression levels are comparable between the different Spikes, which would be the more appropriate indicator to look at for expression. Of note, it is our view that is difficult to assume too much related to packaging efficiency based on Spike expression alone, because there is not always a good directly proportional relationship between Spike incorporation and infectivity, as described in Gallo G. et al., (2026) : Heart-nosed bat alphacoronaviruses use human CEACAM6 to enter cells | Nature. These authors are unable to deduce a strong correlation between results from purified pseudoparticle immunoblots with pseudotype entry values. Lentiviral pseudoparticles also likely non-specifically trans-incorporate viral glycoproteins into their surface. This along with the observation by Gallo G et al., suggest it is difficult to establish a minimum threshold for pseudovirus packaging. Therefore, we believe that our current criteria of seeing detectable protein in an immunoblot is a reasonable criterion, albeit qualitative.

However, of note we do normalise the virus used in our assays (receptor usage screen and pseudovirus neutralisation assays). In the methods section for the receptor screen, we state: *"The next day, cells were re-plated into white-bottomed 96-well plates at 2x10^4/well* ***and infected with pseudoparticles equivalent to 10^5 to 10^6 relative light units (RLU)*** *for 48 h at 37°C, 5% CO_2."* (line 170-172). We appreciate however, that this may not be clear so have expanded the method to explicitly explain this. Lines 172-176 now reads: *"The next day, cells were re-plated into white-bottomed 96-well plates at 2x10^4/well and infected with* ***pseudoparticles equivalent to 10^5 to 10^6 relative light units (RLU), pre-determined by titrating on BHK-21 cells overexpressing Rh. cornutus ACE2 (Rc-o319), Rh. alycone ACE2 (RhGB07, RfGB02) or human ACE2 (all others) and the volume of pseudoparticles added to the assay was adjusted accordingly. For non-ACE2 using pseudoviruses, a fixed volume of 100μL was used in subsequent assays. Transfected cells were incubated with pseudotyped virus*** *for 48 h at 37°C, 5% CO_2."*

We have also added the pseudovirus titrations in BHK-21 cells overexpressing human ACE2 for all viruses except Rc-o319 (titrated instead on cells overexpressing *Rh. cornutus* ACE2), and RfGB02/RhGB07 (titrated on cells overexpressing *Rh. alycone* ACE2) (new **Supplementary Figure 2A, see below**). This we hope, illustrates more clearly the differences in titre for each virus, and how we adjust the virus volume added to the assay to account for this. We have also edited the main text (line 419-421) to reflect this, which now reads: *"Transfected cells were subsequently transduced with* ***a fixed volume of*** *bat sarbecovirus Spike-based pseudotypes* ***equivalent to 10^5 – 10^6 relative light units*** *(those highlighted in red in Figure 1A, S2 and S2 Tables;* ***Supp Figure 2A****)."* Similarly, the method for the micro virus neutralisation assay reads (lines186-188): "Convalescent plasma/serum or mAbs were incubated with a fixed titred volume of sarbecovirus pseudoparticles equivalent to 10^5 – 10^6 signal luciferase units and incubated for 1h at 37°C and 5% CO_2."

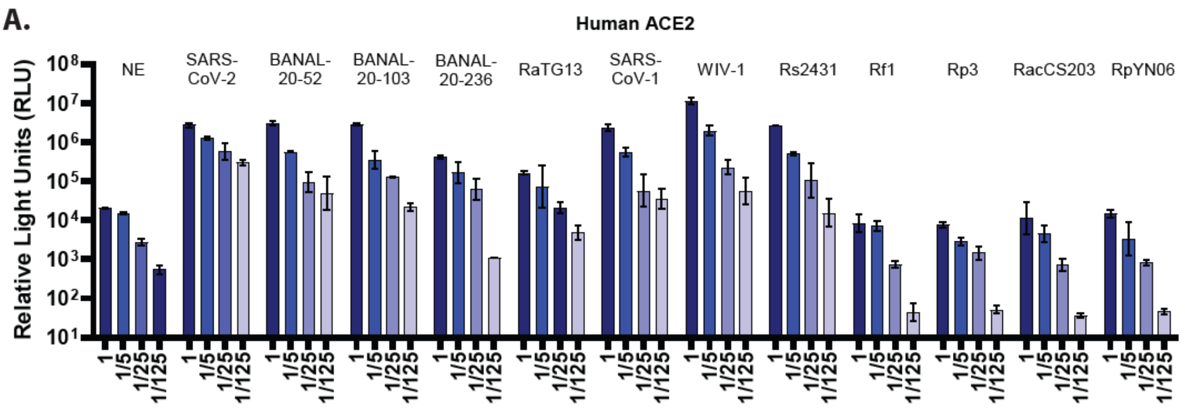

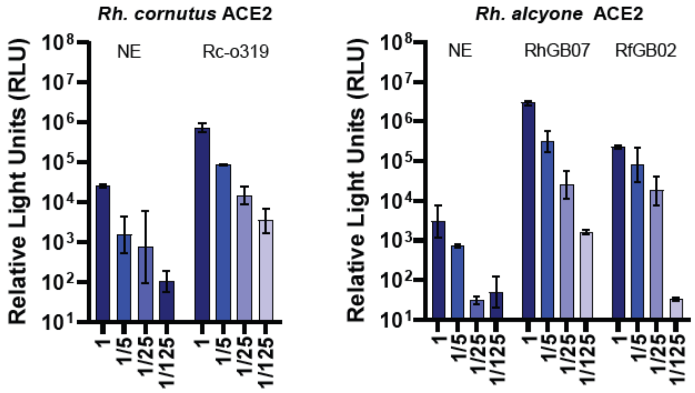

Legend ***Supplementary Figure 2:*** ***(A)*** *Lentiviral pseudotypes expressing different bat sarbecovirus Spikes were titrated on BHK-21 cells overexpressing human ACE2 (SARS-CoV-2, BANAL-20-52, BANAL-20-103, BANAL-20-236, RaTG13, SARS-CoV-1, WIV-1, Rs4231, Rf1, Rp3, RacCS203, RpYN06), Rh. cornutus ACE2 (Rc-o319) or Rh. alcyone ACE2 (RhGB07, RfGB02). Virus titres were used to normalise input virus for subsequent assays.*

4. **Based on the data in Fig. 1 and Fig. 3, SARS-CoV-1 should be regarded as a generalist, and I was wondering why the authors excluded SARS-CoV-1 from the antigenicity characterization?**

We agree with the reviewer that SARS-CoV-1 would be regarded as a generalist, albeit to a lower degree than the clade 1b sarbecoviruses. We did not include SARS-CoV-1 in the antigenicity characterisation as unfortunately we were unable to successfully make the SARS-CoV-1 RBD for use in ELISAs. This is a problem that we occasionally encounter – a small percentage of RBDs appear refractory to production and/or purification. We did however, successfully make and include WIV-1 as an alternative (93.1% amino acid identity to SARS-CoV-1 RBM, https://journals.asm.org/doi/10.1128/jvi.00342-24) as well as Rs4231, which are both from clade 1a and show similar patterns of receptor usage to SARS-CoV-1. Further, our focus with the antigenicity studies was the potential emergence of other bat sarbecoviruses, using SARS-CoV-2 as the comparator, in essence to understand whether pre-existing SARS-CoV-2 immunity could be effective against other bat sarbecoviruses. We hope the reviewer agrees that in this context the inclusion of other clade 1a viruses to compare with SARS-CoV-2 is sufficient. Nonetheless, we have expanded the main text to make this aim clearer on lines 638-639: *"To ascertain whether pre-existing immunity following infection with SARS-CoV-2 can provide cross-protection against related **bat sarbecoviruses should they emerge in the human population**, we examined their antigenic similarity by ELISA and VNT…".*

5. **Most of the sarbecoviruses selected in this study are closely related to SARS-CoV-2; thus, a high level of cross-neutralization activity is expected and has been previously demonstrated. It would be nice if the authors could include more SARS-CoV-1–related animal CoVs in this study, such as those from different subclades in Fig. 1a (e.g., YN2018B, Rs4874, LYRa11, RsSHC014).**

The sarbecoviruses selected for this study were chosen because we wanted to understand how small differences in Spike (i.e., those viruses closely related to SARS-CoV-2 that could have a higher likelihood of spillover), affect host range and antigenicity. Accordingly, we selected all of the clade 1b bat sarbecovirus Spikes that were available at the time, which included the BANAL viruses and RaTG13. At the time of selection, the RShSTT182 / EPI_ISL_852604 and RShSTT200 / EPI_ISL_852605 clade 1b Spike sequences were not available, but similar studies by Hu Y et al. have shown that the RBD of these viruses bound with similar affinities as SARS-CoV-2 RBD to different bat ACE2 orthologues (https://link.springer.com/content/pdf/10.15252/embj.2022111737). Furthermore, we do include some representative, more divergent sarbecovirus Spike from other clades (1a, 2, 3 and 5) to broaden this analysis and show that they are also neutralised by SARS-CoV-2 derived convalescent sera and monoclonal antibodies (Figures 5B and C, Figure 7B). With regards to including more SARS-CoV-1 related animal CoVs, we admit that the selection of viruses for this study was conducted some time ago, and they might appear outdated. To examine this we compared the amino acid sequence similarity of SARS-CoV-1 to WIV-1, Rs4231 and the viruses you suggest (YN2018B, Rs4874, LYRa11 and RsSHC014), most of which share >94% identity to SARS-CoV-1 (WIV-1 94.6%, LYRa11 94.1%, YN2018B 95.5%, Rs4974 95.0%). Interestingly, Rs4231 was still the most divergent, with 82% amino acid identity to SARS-CoV-1. RsSHC014 also shared 82% amino acid similarity to SARS-CoV-1 and shares 99.5% similarity to Rs4231. We believe therefore that we have reasonably good coverage of the diversity within the clade1a subclade already. However, we thank the reviewer for raising this suggestion.

6. **Line 691–693: This statement may not be true since more sarbecoviruses are yet to be discovered, and this study does not include all known sarbecoviruses. As observed in SARS-CoV-2 evolution, early SARS-CoV-2 vaccine-induced neutralizing antibodies failed to neutralize existing circulating variants, e.g., NB.1.8.1 or XFG.**

We agree with the reviewer that this may not be true in the context of yet undiscovered sarboecoviruses. In the context of SARS-CoV-2 evolution, this virus and its variants have undergone (and continue to undergo) specific adaptation to human ACE2 usage and constant immune pressure; a pressure likely not equivalent to that seen in bats. Nonetheless, we have changed the text to clarify this and caveat our conclusions to highlight that we are talking just about the sarbecoviruses we have tested and that this should not be generalised to unknown sarbecoviruses. The next now reads (now lines 762-765): *"These data, combined with high post-pandemic levels of COVID-19 immunity in human populations, may mean the risk of future sarbecovirus zoonotic spillovers,* ***at least the ones described in this study, is diminished. However, additional investigation would be required for other sarbecoviruses, known or unknown, but such studies would provide a strong basis to understand spillover potential."***

Minor comments:

RaTG13 RBD carries 22 amino acid differences from SARS-CoV-2; in my personal view, RaTG13 is quite different from SARS-CoV-2.

We have changed the text in the manuscript on line 60, removing the word "highly" and including the % similarity between the two at the amino acid level. The text now reads: *"Although RaTG13, isolated from a Rhinolophus affinis horseshoe bat,* ***is similar*** *to SARS-CoV-2 at the RBD amino acid level* ***(90%)****, we previously showed it does not share the same broad generalist ACE2 usage profile [15]."*

Reviewer #4:

Here, Thakur, Newman, and colleagues explore the host usage and antigenicity of bat coronavirus spike proteins, focusing on ACE2-using viruses. They describe two viral phenotypes - a generalist and a specialist - and characterize the molecular structure features of receptor-spike interactions using various assays including binding and pseudovirus entry assays. They also report a cryo-EM structure of the RHGB07 spike glycoprotein ectodomain in its apo form. Finally, the authors examine the antigenicity of the different viral spike receptor-binding domains in the context of human serum antibody binding. Overall, the authors point to the generalist ACE2-using bat sarbecoviruses being structurally and antigenically very similar to SARS-CoV-2.

The manuscript reports a wealth of technically-sound functional data, but it is, overall, a very descriptive analysis as there is little correlation observed within and across the generalist and the specialist virus types. Additionally, major caveats stem from the structural analyses of only the apo RhGB07 spike structure.

Major critiques:

1 - structure determination of RhGB07: the data processing details in Figure S5 report over 2M particles picked with template picker, yet only a fraction of those (20K) went into the final 3D reconstruction and refinement.
a) If you are using only 1% of the initial particle stack, then surely, you are missing alternative conformations, other molecular species etc. The data should be carefully re-processed.

We thank Reviewer 4 for highlighting the particle retention rate. The high initial count (>2 million extracted) resulted from an intentionally permissive picking threshold designed to maximize the capture of all potential protein states to avoid missing rare conformations or complexes. As shown in the updated Supplementary Fig. 5 (**now Supp Figure 6**), the vast majority of these initial picks were identified as false positives (e.g., ice contaminants, carbon edges, or aggregates) through multiple rounds of 2D classification. To address the concern regarding alternative conformations, we performed extensive classification with heterogenous refinement on the cleaned particle stack. These efforts consistently yielded only the closed-state trimer; no subclasses representing RBD-up or other conformational states were identified.

Our findings are consistent with recent studies of other Clade 3 Spike trimers (Lee, J. et al 2025 Broad receptor tropism and immunogenicity of a clade 3 sarbecovirus: Cell Host & Microbe and Wang, J. et al 2025 SARS-related coronavirus S-protein structures reveal synergistic RBM interactions underpinning high-affinity human ACE2 binding | Science Advances) where authors also observed particles for full-length Spike that appear to only preferentially adopt a closed conformation. Their results did not reveal full-length Spikes in any other conformation. We have clarified this filtering process in the Materials and Methods (lines 257-267) and updated the workflow figure to better illustrate the transition from raw picks to the final, clean particle stack. The text now reads: *"**The 2D classes already show a trimeric spike resembling a closed conformation.** These particles were sufficient to produce an ab-initio model and can be refined to 3.66 Å with C3 symmetry. To increase the resolution and quality of the map these 2D classes were used once again for template picking which led to 2 234 763 particles. After several rounds of extracting, cropping **(Box size 500 pixel, Crop size 300 pixel)** and classification only 21 821 particles remained **(3.09 Å, C3 symmetry)** and were subjected to heterogenous refinement **(C1 symmetry)** with 4 classes. The 3 best classes in terms of resolution were used (17 685 particles) for a final round of NU-Refinement leading to a map of 2.97 Å (FSC 0.143, C3 symmetry). This map showed good local resolution at the RBD without necessitating for local refinement for this domain. **Due to cropping during particle extraction, the FSC curve is truncated at the Nyquist limit of 2.77 Å and does not fully decay to zero."***

We have updated Supplementary Figure S5 (**now Supp Figure 6, see below**) to show more micrographs and the state of the grid when collected, as well as the multitude of junk particles that were picked and not retained leading to the low percentage of particles.

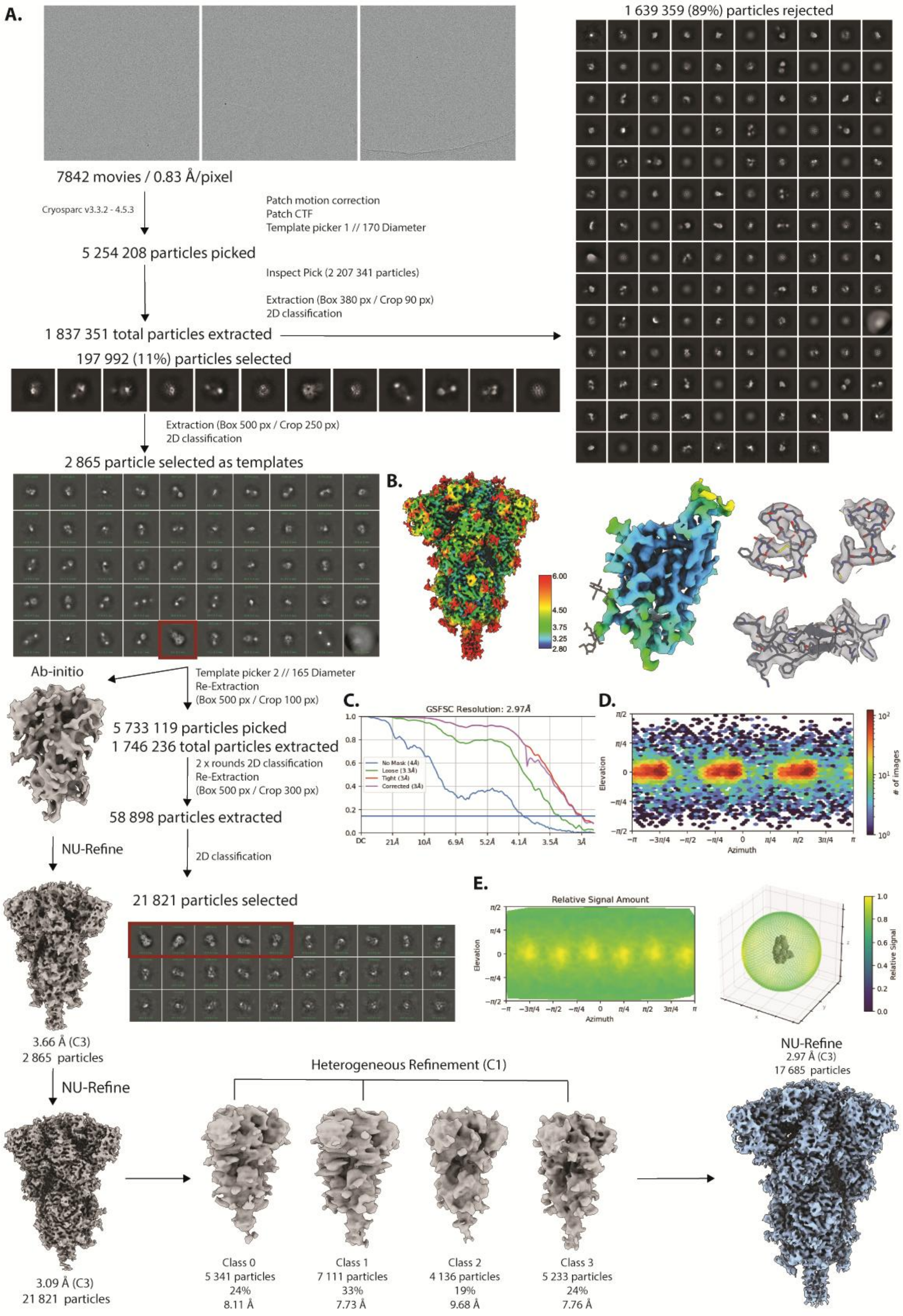

Legend ***Supplementary Figure 6: Cryo-EM Processing Workflow (A)*** *Processing workflow of for the determination of the RhGB07 full-length trimeric spike.* ***(B)*** *Cryo-EM density coloured by local-*

resolution of the full-length trimer and of the isolated RBD. Selected regions represented with their cryo-EM density is also shown. ***(C)*** *FSC curve showing a determined resolution for the full-length spike of 2.97 Å* ***(D)*** *Viewing direction distribution of particles used in the reconstruction.* ***(E)*** *Relative Signal vs Viewing direction plot of particles. Cryo-EM data collection, refinement and validation statistics can be sound in* ***S3 Table****.*

b) Refinement is only done with imposed C3 symmetry and, therefore, any RBD up/down variations would have been missed due to averaging.

We once again thank Reviewer 4 for their attention to detail. To isolate potential conformations that may have been grouped together and would be missed if refinement was done with C3 symmetry, we did perform heterogenous refinement with C1 symmetry over 4 classes that revealed no visual evidence of any up-RBD and only 3-down. For the final refinements, we imposed C3 symmetry after visual inspection of a C1 map did not yield significant differences or improvements to a C3 map, so have only shown this refinement.

c) Supposedly, ACE2 was included in the sample, but was not detected on cryo-EM grids. Was ACE2 excluded from the grid holes? Is there evidence of it being in the holes but dissociated? Please provide raw micrograph images and additional 2D processing with, for example, smaller particle picking params.

Recent studies by peer labs also have demonstrated that several Clade 3 full length spikes are difficult to determine a structure in complex with ACE2, Lee, J. et al 2025 (Broad receptor tropism and immunogenicity of a clade 3 sarbecovirus: Cell Host & Microbe) and Wang, J. et al 2025 (SARS-related coronavirus S-protein structures reveal synergistic RBM interactions underpinning high-affinity human ACE2 binding | Science Advances). In both studies, the RBD are in the closed state in context of the full-length spike, as in our study. We do not believe this is due to the cryoEM preparation conditions as Lee, J. et al also noted a lack of open RBD Spikes.

Supplementary Figure S5 (**now Supp Figure 6, as above in point 1a**) has been amended with additional micrographs for inspection as well as fuller representation of the typical 2D classes seen and selected. This highlights that the only observable class with distinct features are closed form full length Spikes with no ACE2 bound or free. We can only speculate that the RhGB07 Spike bound to ACE2 may be inherently physically unstable during freezing on grids. We would also like to bring to the Reviewer's attention that in Lee, K. et al 2025 and Wang, J. et al 2025, they only revealed an ACE2-bound clade 3 RBD using isolated domains, not in a full-length context.

d) In the same vein, the authors should show physical interaction with bat ACE2 and RhGB07 spike using size-exclusion chromatography and additional biophysical methods.

In a previous report from authors on this manuscript (Tan et al, 2023 https://www.nature.com/articles/s41467-023-38717-w#Sec2), authors we performed biophysical binding experiments via BLI, to demonstrate that the RhGB07 Spike can interact with human, *Rh. ferrumequinum* and *M. lucifugus* ACE2. Solely based on these results we attempted to resolve a complex structure of either human or R. ferrumenquinum ACE2-bound to the RhGB07 spike. We did this by mixing the two proteins, as we have done previously for many other full-length Spike-ACE2 complexes (Ni et al, 2023 Cryo-EM structures and binding of mouse and human ACE2 to SARS-CoV-2 variants of concern indicate that mutations enabling immune escape could expand host range | PLOS Pathogens) and so do not have any size-exclusion results to show a complex being formed off grid.

e) Atomic model validation is missing

Thank you Reviewer 4 for letting us know that we forgot to attach the validation report. The PDB Validation report is attached in this newest version. We would like to bring attention to the Supplementary Table 3 as well, that also shows atomic validation statistics.

2 - Epitope binning in Figure 6 should be done with Fabs and not full IgG molecules because of steric hindrance on such a small protein domain, as is RBD.

We thank the reviewer for this comment and appreciate their concerns. The epitope binning results align with the predicted binding groups and published epitope groups for the RBD giving us confidence that steric hinderance is not a major factor in the epitope communities we have identified through the analysis. Whilst binning with Fabs gives the blocking or permissiveness using the exact footprint of the epitope, using full antibody gives a more biologically relevant picture of how epitopes overlap given that it will be full antibodies present in the host mammal. Multiple published papers have also used full antibody with RBD to perform epitope binning by BLI or SPR (see below) so we are confident that our data is robust.

References:

- Epitope Classification and RBD Binding Properties of Neutralizing Antibodies Against SARS-CoV-2 Variants of Concern - PMC
- Identification of a highly conserved neutralizing epitope within the RBD region of diverse SARS-CoV-2 variants | Nature Communications
- Integrating artificial intelligence-based epitope prediction in a SARS-CoV-2 antibody discovery pipeline: caution is warranted

Minor:

1 - which PDBs were used for Figure 2B? Is it fair to compare apo RBD structures with those complexed with ACE2 and draw conclusions about crucial binding loop rearrangements?

We thank the reviewer for bringing up this point. The PDBs for SARS-CoV-2 and SARS-CoV-2 have now been added to the Figure 2B figure legend. We have now replaced the apo RBD vs RBD complexed with ACE2, with RBDs that are all either from apo or closed state structures to avoid drawing unfair conclusions about crucial binding loop arrangements (**see below**).

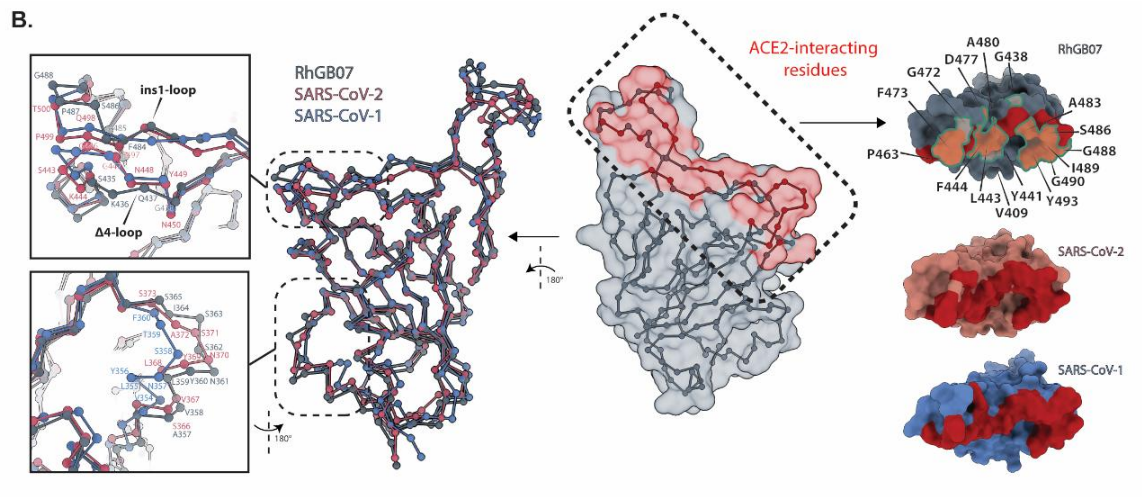

*Legend **Figure 2: (B)** The C-alpha trace of the RBD of RhGB07 rendered as balls-and-sticks superimposed with the RBD of SARS-CoV-1 (PDB 7DF3) and SARS-CoV-2 (PDB 5X5B). The insets show a zoomed-in view of two regions of the RBD that show the greatest changes in the position of the C-alpha of the RBD. The region containing the ins1-loop and delta4-loop coincides with putative ACE2-interacting residues based on mapping the equivalent residues of SARS-CoV-1 and SARS-CoV-2. The ACE2 interacting residues were determined by superimposing SARS-CoV-1 and RhGB07 RBD onto the SARS-CoV-2 RBD – ACE2 (PDB: 6M0J) structure and identifying interacting residues. Highlighted residues of RhGB07 show the putative interacting residues that differ in identity to SARS-CoV-2.*

Journal Requirements:

1) Please ensure that the funders and grant numbers match between the Financial Disclosure field and the Funding Information tab in your submission form. Note that the funders must be provided in the same order in both places as well.
State what role the funders took in the study. If the funders had no role in your study, please state: "The funders had no role in study design, data collection and analysis, decision to publish, or preparation of the manuscript."

A line for this has been added in the funding section, line 386.

2) We ask that a manuscript source file is provided at Revision. Please upload your manuscript file as a .doc, .docx, .rtf or .tex.

This has been done as requested.

3) Please provide separate figure files in .tif or .eps format. For more information about figure files please see our guidelines:

https://journals.plos.org/plosbiology/s/figures

4) We notice that your supplementary figures and tables are included in the manuscript file. Please remove them and upload them with the file type 'Supporting Information'. Please ensure that each Supporting Information file has a legend listed in the manuscript after the references list.

This has been done as requested.

5) We do not publish any copyright or trademark symbols that usually accompany proprietary names, eg ©, ®, ™ (e.g. next to drug or reagent names). Please remove all instances of trademark/copyright symbols throughout the text, including: ™ on page 11.

This has been done for the two occurrences in the Surface Plasmon Resonance method (lines 293 and 303).

6) Figures 1, and S1: please (a) provide a direct link to the base layer of the map (i.e., the country or region border shape) and ensure this is also included in the figure legend; and (b) provide a link to the terms of use / license information for the base layer image or shapefile. We cannot publish proprietary or copyrighted maps (e.g. Google Maps, Mapquest) and the terms of use for your map base layer must be compatible with our CC BY 4.0 license.

Note: if you created the map in a software program like R or ArcGIS, please locate and indicate the source of the basemap shapefile onto which data has been plotted.

If your map was obtained from a copyrighted source please amend the figure so that the base map used is from an openly available source. Alternatively, please provide explicit written permission from the copyright holder granting you the right to publish the material under our CC BY 4.0 license.

If you are unsure whether you can use a map or not, please do reach out and we will be able to help you. The following websites are good examples of where you can source open access or public domain maps:
*** U.S. Geological Survey (USGS) - All maps are in the public domain. (http://www.usgs.gov)**
*** PlaniGlobe - All maps are published under a Creative Commons license so please cite "PlaniGlobe, http://www.planiglobe.com, CC BY 2.0" in the image credit after the caption. (http://www.planiglobe.com/?lang=enl)**
*** Natural Earth - All maps are public domain. (http://www.naturalearthdata.com/about/terms-of-use/)**

Terms of use for licencing for maps obtained from MapChat under the CC BY-SA 4.0 licence: https://www.mapchart.net/terms.html#licensing-maps. The figure legends for **Fig 1B** and **Supp Fig 1** have been amended to include citation for the map modified from MapChat and the CC licence.

Your revisions should address the specific points made by each reviewer. Please submit the following files along with your revised manuscript:

1. A 'Response to Reviewers' file - this should detail your responses to the editorial requests, present a point-by-point response to all of the reviewers' comments, and indicate the changes made to the manuscript.
***NOTE: In your point-by-point response to the reviewers, please provide the full context of each review. Do not selectively quote paragraphs or sentences to reply to. The entire set of reviewer comments should be present in full and each specific point should be responded to individually, point by point.**
You should also cite any additional relevant literature that has been published since the original submission and mention any additional citations in your response.

We have done this, and also updated the phylogenetic tree in **Fig 1A** with additional RBD sequences made available on NCBI since our initial submission, as well as including relevant references.

2. In addition to a clean copy of the manuscript, please also upload a 'track-changes' version of your manuscript that specifies the edits made. This should be uploaded as a "Revised Article with Changes Highlighted" file type.

Attached as requested.

Re-submission Checklist
When you are ready to resubmit your revised manuscript, please refer to this re-submission checklist: https://plos.io/Biology_Checklist

To submit a revised version of your manuscript, please go to https://www.editorialmanager.com/pbiology/ and log in as an Author. Click the link labelled 'Submissions Needing Revision' where you will find your submission record.
