# Supplementary material for: Breadth of ACE2 receptor usage predicts host range and antigenic relatedness across bat sarbecoviruses
Source: PLoS Biol. 2026 Oct 1;24(10):e3003944. doi: 10.1371/journal.pbio.3003944 (PMC13630225; doi:10.1371/journal.pbio.3003944)
Supplement: Supplementary file 3 [file pbio.3003944.s022.docx]

**PBIOLOGY-D-25-03032R3: Response to Reviewers: June 2026**

Dear Editors and Reviewers,

We would like to thank you for your positive comments on the changes we have made to the initial manuscript and believe that the suggestions you have made have further strengthened the manuscript. We have addressed the additional reviewer comments point-by-point below, providing additional validation, explanation and/or data as appropriate, which we hope will mitigate any outstanding concerns.

Yours sincerely,

Drs. Nazia Thakur and Dalan Bailey

**Reviewer #1:

The authors have added substantial data to this manuscript that satisfies all of my previous comments.**

Thank you.

----------------------------------------------------------------

**Reviewer #2:

I appreciate the authors' efforts in revising the manuscript and addressing the concerns raised in the previous review. In particular, the newly added Δ4-loop mutagenesis and ELISA assays provide important functional support for the structural findings. The inclusion of cell-cell fusion data, comparisons with recent clade 3 sarbecovirus structures, and an expanded discussion of ACE2-binding interfaces and receptor tropism further improve the clarity and context of the study. I have only a few minor comments that the authors may wish to consider prior to publication:**

We thank the reviewer for their positive feedback on the updated manuscript and have addressed their minor comments below:

**1. RhGB07 - R. ferrumequinum ACE2 interaction and complex**

**The authors suggest that differences between UK and Chinese R. ferrumequinum ACE2 proteins may explain the discrepancy between binding and entry phenotypes. What is the sequence identity between these ACE2 proteins? Without sequence information, authors should explicitly say this explanation is speculative. In addition, RhGB07 did not mediate detectable pseudovirus entry or cell-cell fusion through R. ferrumequinum ACE2 in the current study. Therefore, the rationale for pursuing structural characterization of this complex remains unclear. Remove this statement if possible.**

Agreed. We were unable to find the UK Rh. ferrumequinum sequence to compare amino acid identity and therefore agree our comments in the previous rebuttal were far too speculative. We now retract this text from our rebuttal and remove any mention of this in the main manuscript. We have also removed text explaining the historical decision-making process for attempting complex formation between the RhGB07 RBD and Rh. ferrumequinum. Of note, we would like to draw the reviewer’s attention to the new data in our revision (**Figure 2E**) which provides deep mechanistic insight into why Rh. ferrumequinum is not used by RhGB07. In these experiments we added SARS-CoV-2-like substitutions into the RhGB07 RBD and conferred functional Rh. ferrumequinum ACE2 usage.

**2. FSC curve**

**The explanation that the FSC curve is truncated due to particle cropping is reasonable. However, this remains an interpretation rather than a direct validation. I suggest re-extracting the final particles without Fourier cropping and performing a final refinement. If the FSC curve then decays toward zero while maintaining a comparable resolution estimate, this would directly confirm the authors' explanation.**

We appreciate this constructive suggestion and have now conducted the direct validation the reviewer recommends.

To address the concern, we re-downloaded our publicly available dataset (EMPIAR 12921), reimported the previously identified particles leading to our original map reconstruction into Cryosparc, and performed additional tests as suggested:

**1. Reproducibility with the current reported binning (0.83 Å/pix, 500→300 px box)**

Particle re-extraction using identical binning parameters yielded identical NU-refinement (C3) results and FSC curves as in the original study, confirming our processing pipeline consistency and the premature cutoff due to reaching the Nyquist limit of 2.76Å.

**2. Duplicate removal validation**

We ran the "Remove Duplicates" job within Cryosparc and detected zero duplicates within the 17,685 particle dataset, ruling out duplicate particles as a confounding variable. Refinement of this curated dataset produced identical results.

**3. Direct FSC validation without Fourier cropping:**

Critically, we re-extracted the same particles *without binning* (preserving the full 500 px box) and subjected them to identical NU-refinement. The FSC curve now properly decays to near zero at high frequencies far from the Nyquist limit, while maintaining the same resolution limit as with the map obtained with binning.

We believe these tests demonstrate that the over binning of data for computational performance reasons was the origin of the non-decay to zero of the FSC curve.

We do not believe this has any impact on the analysis on the model built into the previous map (binned) and does not warrant any changes to the structure presented in the manuscript. A comparison of the two maps (left, yellow, unbinned vs. right, brown, binned) with the current atomic model (9RDT), show nearly identical density features at a similar density threshold cutoff.

We have also now deposited the map (unbinned) with an EMDB code of EMD-58663 for those who wish to compare further in detail.

**3. SPR data transparency
The explanation regarding the apparent discrepancy between ELISA, SPR, and neutralization data is appreciated. However, the interpretation remains difficult to evaluate from summarized KD, EC50, and IC50 values alone.
I recommend providing the raw SPR sensorgrams for representative antibodies, particularly VA14_26 against WIV1, BANAL viruses, Rc-o319, and RhGB07, in the Supplementary Information. These data would allow readers to better assess the quality of the fits and reconcile the ELISA, SPR, and neutralization datasets.**

**Overall, the manuscript has been substantially strengthened and minor improvement may be needed for publication.**

We thank the reviewer for this suggestion and have now included the SPR sensograms generated for this data, for an exemplar mAb: VA14_26. This shows a representative dataset of high (SARS-CoV-2, BANAL-20-52, BANAL-20-236, Rs4231), medium (WIV-1, BANAL-20-103), low (Rc-o319) and no (RhGB07) binding, as well as the associated K_D_ values. We hope this now makes interpretation of the data in Figure 7 clearer for the reader. This figure will be presented as a new **Supp Fig (12C)** and will be cited on lines 742 and 725, and the figure legend (line 1257) amended accordingly**.**

*Legend:* ***Supp Figure 12 (C)*** *Representative SPR kinetics binding curves showing the association and dissociation of mAb VA14_26 with bat sarbecovirus RBDs. Each line represents a RBD concentration, starting at 5µg/ml and titrated 2-fold (8 dilutions). Calculated K_D_ values are also shown.*

----------------------------------------------------------------

**Reviewer #3:

The authors have adequately addressed my concerns.**

We thank the reviewer for their positive response to our changes.

**1) However, I was somewhat surprised that the authors were unable to purify the SARS-CoV-1 RBD, given that there are many commercial sources available for this protein. Therefore, this may not be a sufficiently strong justification for excluding it from the current study. Although SARS-CoV-1, WIV-1, and Rs4231 all belong to clade 1a sarbecoviruses, they are still phylogenetically and antigenically distinct from one another.**

While we agree with the reviewer that SARS-CoV-1 is antigenically and phylogenetically distinct from Rs4231 (~94.4% identity for whole Spike, ~80.8% identity for RBD), it is very closely related to WIV-1 - indeed at one point WIV-1 was considered the progenitor (~92% identity for whole Spike, ~94.3% identity for RBD). WIV-1 is also a major focus of our sister paper, also submitted to PLOS Biology. Various studies have reported comparable neutralisation titres between SARS-CoV-1 and WIV-1 using SARS-CoV-2 convalescent and vaccinee sera ([Broad cross neutralizing antibodies against sarbecoviruses generated by SARS-CoV-2 infection and vaccination in humans | npj Vaccines](https://www.nature.com/articles/s41541-024-00997-8?utm_source=chatgpt.com)), or alternatively using monoclonal antibodies generated against SARS-CoV-2 ([Broad sarbecovirus neutralization by a human monoclonal antibody | Nature](https://www.nature.com/articles/s41586-021-03817-4?utm_source=chatgpt.com), [Cross-neutralization of SARS-CoV-2 by a human monoclonal SARS-CoV antibody | Nature](https://www.nature.com/articles/s41586-020-2349-y)). At the time we considered Rs4231 and WIV-1 as good representatives of clade 1a viruses, as our paper is ostensibly about risk assessing non-emerged bat sarbecoviruses, not viruses that have already spilled over (such as SARS1). Another additional aspect to consider is that the reagents in this study were all made in-house. We have had issues comparing commercial and home-made reagents in side-by-side experiments in the past – a practical but major reason we did not purchase the SARS1 RBD.

**2) Although the authors have provided ELISA data for RhGB07 RBD binding to Rh. alcyone ACE2, as well as Kd values for RhGB07 RBD interactions with other bat and human ACE2 receptors published elsewhere, ELISA assays do not directly measure binding affinity. Therefore, it would be preferable for the authors to perform biolayer interferometry (BLI) or surface plasmon resonance (SPR) assays to accurately determine the binding affinities.**

Again, we thank the reviewer for this suggestion; however, we do not feel that the additional SPR will change the interpretation of our papers results at this late time. In the previous manuscript, where RhGB07 was first described (<https://www.nature.com/articles/s41467-023-38717-w#Sec2>), binding of RhGB07 RBD to *M.lucifugus* ACE2 was greater than that of human ACE2 (K_D_ of 209nM compared to 253nM) but still low compared to published affinities for SARS2. Herein, we simply wanted to use qualitative assays (ELISA) to rank RhGB07 RBD binding to *M.lucifugus*, *Rh. alcyone* and human ACE2, in this wider published context. Subsequently, we compared this to SARS-CoV-2 RBD and showed lower binding, concordant with our entry data (**Supp Figure 5D**). This, together with the mechanistic identification of amino acids important in the restricted host range of RhGB07, shown in the newly added data (**Figure 2F**) provides detailed mechanistic explanation of why some sarbecovirsues are specialised to a limited number of receptors. We have included a line in the main text to clarify in the results that the binding approach we have used is semi-quantitative, and have updated the discussion to highlight the limitations.

The text in the results section reads (lines 460-461): “ … however, these assays appeared to be less sensitive **and do not allow determination of affinity, rather provide an approach to relatively rank binding across ACE2 orthologues** (**Supp Figure 5C**).”

The text in the discussion section reads (lines 795-801): “The clade III viruses RhGB07 and RfGB02 had restricted bat and slightly broader mammalian ACE2 usage, with RhGB07 being able to utilise hACE2, as previously described [10], **with RhGB07 RBD exhibiting lower binding by ELISA to *Rh. alycone, M. lucifugus* and human ACE2, compared to SARS-CoV-2 RBD (Supp Figure 5D). Quantifying K_D_ values of these binding interactions by surface plasmon resonance or by bio-layer interferometry would elucidate the affinity of RhGB07 RBD with these ACE2s, to better understand the interplay of affinity with receptor entry.”**

We really hope this additional explanation satisfies the reviewer’s remaining concerns.
