# Supplementary material for: Breadth of ACE2 receptor usage predicts host range and antigenic relatedness across bat sarbecoviruses
Source: PLoS Biol. 2026 Oct 1;24(10):e3003944. doi: 10.1371/journal.pbio.3003944 (PMC13630225; doi:10.1371/journal.pbio.3003944)
Supplement: Peer review history

# Editor report: Decision Letter 0

**DOI:** 10.1371/journal.pbio.3003944.r001

**Authors:** Melissa Vazquez Hernandez

30 Sep 2025

Dear Dr Thakur,

Thank you for submitting your manuscript entitled "Understanding the future risk of bat coronavirus spillover into humans – correlating sarbecovirus receptor usage, host range, and antigenicity." for consideration as a Research Article by PLOS Biology.

Your manuscript has now been evaluated by the PLOS Biology editorial staff, as well as by an academic editor with relevant expertise, and I am writing to let you know that we would like to send your submission out for external peer review.

However, before we can send your manuscript to reviewers, we need you to complete your submission by providing the metadata that is required for full assessment. To this end, please login to Editorial Manager where you will find the paper in the 'Submissions Needing Revisions' folder on your homepage. Please click 'Revise Submission' from the Action Links and complete all additional questions in the submission questionnaire.

Once your full submission is complete, your paper will undergo a series of checks in preparation for peer review. After your manuscript has passed the checks it will be sent out for review. To provide the metadata for your submission, please Login to Editorial Manager (https://www.editorialmanager.com/pbiology) within two working days, i.e. by Oct 02 2025 11:59PM.

If your manuscript has been previously peer-reviewed at another journal, PLOS Biology is willing to work with those reviews in order to avoid re-starting the process. Submission of the previous reviews is entirely optional and our ability to use them effectively will depend on the willingness of the previous journal to confirm the content of the reports and share the reviewer identities. Please note that we reserve the right to invite additional reviewers if we consider that additional/independent reviewers are needed, although we aim to avoid this as far as possible. In our experience, working with previous reviews does save time.

If you would like us to consider previous reviewer reports, please edit your cover letter to let us know and include the name of the journal where the work was previously considered and the manuscript ID it was given. In addition, please upload a response to the reviews as a 'Prior Peer Review' file type, which should include the reports in full and a point-by-point reply detailing how you have or plan to address the reviewers' concerns.

During the process of completing your manuscript submission, you will be invited to opt-in to posting your pre-review manuscript as a bioRxiv preprint. Visit http://journals.plos.org/plosbiology/s/preprints for full details. If you consent to posting your current manuscript as a preprint, please upload a single Preprint PDF.

Feel free to email us at plosbiology@plos.org if you have any queries relating to your submission.

Kind regards,

Melissa

Melissa Vazquez Hernandez, Ph.D.

Associate Editor

PLOS Biology

mvazquezhernandez@plos.org

# Peer review: Decision Letter 1

**DOI:** 10.1371/journal.pbio.3003944.r002

**Authors:** Melissa Vazquez Hernandez

5 Dec 2025

Dear Nazia,

Thank you for your patience while your manuscript "Understanding the future risk of bat coronavirus spillover into humans – correlating sarbecovirus receptor usage, host range, and antigenicity." was peer-reviewed at PLOS Biology. It has now been evaluated by the PLOS Biology editors, an Academic Editor with relevant expertise, and by four independent reviewers.

In light of the reviews, which you will find at the end of this email, we would like to invite you to revise the work to thoroughly address the reviewers' reports. As you will see below, majority of reviewers are positive about the relevance and novelty of the study, yet some concerns have raised during revision regarding missing data to support the hypotheses. Reviewer 1 requests validation of the RBD/ACE2 interaction hypothesis, to provide further support of the RBD vs human/bat ACE2 interactions like protein purification and mutations, and to clarify some claims. Reviewer 1 raised some concerns about the structural analysis, asking for quantitative RBD–ACE2 binding data, raw fusion images, clarification of missing ACE2-bound structures, corrections to structural comparisons and FSC curves, resolution of contradictions in antibody binding versus neutralization, and integration of recent literature on RBM architecture and ACE2-binding interfaces. S/He also mentions that obtaining at least one RhGB07 Spike structure would be ideal to have more appropriate controls. Reviewer 3 notes that much of the ACE2-usage background is already known and argues that the manuscript’s novelty depends on strengthening the RhGB07 structural and functional characterization, requesting binding affinity data, structural analysis, clearer pseudovirus normalization, inclusion of SARS-CoV-1 in antigenicity analyses, and broader sampling of SARS-CoV-1–related sarbecoviruses. Reviewer 4 had concerns with the cryo-EM analysis, such as the low fraction of particles used, the use of strict C3 symmetry potentially obscuring RBD conformations, lack of evidence for ACE2 presence on grids, absence of biophysical validation of Spike–ACE2 interaction, and missing atomic model validation. Additionally, the reviewer questions the epitope binning approach using full IgGs instead of Fabs and the fairness of structural comparisons across apo and ACE2-bound states. We agree with all reviewer concerns and would require some additional experimental revisions to address them, as we consider that this would strengthen the work.

Given the extent of revision needed, we cannot make a decision about publication until we have seen the revised manuscript and your response to the reviewers' comments. Your revised manuscript is likely to be sent for further evaluation by all or a subset of the reviewers.

In addition to these revisions, you will need to complete some formatting changes, which you will receive in a follow up email. A member of our team will be in touch with a set of requests shortly.

We expect to receive your revised manuscript within 3 months. Please email us if you have any questions or concerns, or would like to request an extension. We also understand the holiday period coming, so feel free to e-mail me directly if you require more time already due to this period.

At this stage, your manuscript remains formally under active consideration at our journal; please notify us by email if you do not intend to submit a revision so that we may withdraw it.

**IMPORTANT - SUBMITTING YOUR REVISION**

Your revisions should address the specific points made by each reviewer. Please submit the following files along with your revised manuscript:

1. A 'Response to Reviewers' file - this should detail your responses to the editorial requests, present a point-by-point response to all of the reviewers' comments, and indicate the changes made to the manuscript.

*NOTE: In your point-by-point response to the reviewers, please provide the full context of each review. Do not selectively quote paragraphs or sentences to reply to. The entire set of reviewer comments should be present in full and each specific point should be responded to individually, point by point.

You should also cite any additional relevant literature that has been published since the original submission and mention any additional citations in your response.

2. In addition to a clean copy of the manuscript, please also upload a 'track-changes' version of your manuscript that specifies the edits made. This should be uploaded as a "Revised Article with Changes Highlighted" file type.

*Re-submission Checklist*

When you are ready to resubmit your revised manuscript, please refer to this re-submission checklist: https://plos.io/Biology_Checklist

To submit a revised version of your manuscript, please go to https://www.editorialmanager.com/pbiology/ and log in as an Author. Click the link labelled 'Submissions Needing Revision' where you will find your submission record.

Please make sure to read the following important policies and guidelines while preparing your revision:

*Published Peer Review*

Please note while forming your response, if your article is accepted, you may have the opportunity to make the peer review history publicly available. The record will include editor decision letters (with reviews) and your responses to reviewer comments. If eligible, we will contact you to opt in or out. Please see here for more details:

https://blogs.plos.org/plos/2019/05/plos-journals-now-open-for-published-peer-review/

*PLOS Data Policy*

Please note that as a condition of publication PLOS' data policy (http://journals.plos.org/plosbiology/s/data-availability) requires that you make available all data used to draw the conclusions arrived at in your manuscript. If you have not already done so, you must include any data used in your manuscript either in appropriate repositories, within the body of the manuscript, or as supporting information (N.B. this includes any numerical values that were used to generate graphs, histograms etc.). For an example see here: http://www.plosbiology.org/article/info%3Adoi%2F10.1371%2Fjournal.pbio.1001908#s5

*Blot and Gel Data Policy*

We require the original, uncropped and minimally adjusted images supporting all blot and gel results reported in an article's figures or Supporting Information files. We will require these files before a manuscript can be accepted so please prepare them now, if you have not already uploaded them. Please carefully read our guidelines for how to prepare and upload this data: https://journals.plos.org/plosbiology/s/figures#loc-blot-and-gel-reporting-requirements

*Protocols deposition*

To enhance the reproducibility of your results, we recommend that if applicable you deposit your laboratory protocols in protocols.io, where a protocol can be assigned its own identifier (DOI) such that it can be cited independently in the future. Additionally, PLOS ONE offers an option for publishing peer-reviewed Lab Protocol articles, which describe protocols hosted on protocols.io. Read more information on sharing protocols at https://plos.org/protocols?utm_medium=editorial-email&utm_source=authorletters&utm_campaign=protocols

Thank you again for your submission to our journal. We hope that our editorial process has been constructive thus far, and we welcome your feedback at any time. Please don't hesitate to contact us if you have any questions or comments.

Sincerely,

Melissa

Melissa Vazquez Hernandez, Ph.D.

Associate Editor

PLOS Biology

mvazquezhernandez@plos.org

------------------------------------

REVIEWERS' COMMENTS

------------------------------------

Reviewer #1:

This manuscript maps receptor usage, host range and antigenicity across a panel of bat sarbecovirus spikes and mammalian ACE2 orthologues. It shows that the clade I viruses (which includes SARS-CoV-2 and BANAL isolates) tend to be generalists and able to use receptors across species while clade III and V viruses are more specialist for particular bat ACE2s. They also demonstrate a cryo-EM structure of the a clade-III Spike that shows differences in receptor tropism and helps to explain the shifts in ACE2 usage as SARS-CoV-2 evolved. The work is well done and agrees with previously published work showing the generalist ACE2 binding of SARS-CoV-2.

Major comments below:

1.There is little data supporting the RBD/ACE2 interaction hypothesis. The RhGB07 cryo-EM structure is well done, but the authors discuss that specific deletions and glycans explain specialist tropism without validation. Please discuss the confidence in these conclusions or include additional experiments to validate the findings.

2.Additional data on RBB binding would be supportive of structure claims. Can you support the RhGB07 RBD vs human & relevant bat ACE2 interactions with purified protein? This would convert qualitative binding and entry phenotypes into interpretable KD/kon/koff values and strengthen mechanistic claims. This could be combined with mutations designed from the structural data of predicted loops and deletions.

3.Clarify language in the manuscript. Please replace broad claims ("pan-coronavirus vaccines") with precise phrasing ("pan-sarbecovirus immunogen design informed by conserved epitopes identified here"), and explicitly note that protection against non-sarbecovirus betacoronaviruses is not supported by the data.

------------------------------------

Reviewer #2 (Xiaoli Xiong):

PBIOLOGY-D-25-03032R1

"Understanding the future risk of bat coronavirus spillover into humans - correlating

sarbecovirus receptor usage, host range, and antigenicity."

General Summary

SARS-CoV-1 and SARS-CoV-2, both originating from Rhinolophus bats, underscore the zoonotic risk posed by sarbecoviruses—a genetically diverse group of coronaviruses capable of crossing species barriers. These viruses use the angiotensin-converting enzyme 2 (ACE2) receptor for host cell entry, but their ACE2-binding capacity varies across lineages. Clade I viruses, including SARS-CoV-2 and the BANAL isolates from Laos, display broad or "generalist" ACE2 usage, whereas clades III and V exhibit more restricted, "specialist" patterns. RhGB07, a sarbecovirus identified in the United Kingdom, exemplifies this specialization, binding efficiently to only a limited range of ACE2 receptors. Structural analysis of the RhGB07 Spike protein revealed amino acid motifs responsible for this receptor specificity, offering insight into how host range is determined at the molecular level. The study further examines how receptor usage diversity correlates with antigenic similarity to SARS-CoV-2 and how viral evolution, including that of SARS-CoV-2 variants, affects species tropism. By comparing generalist and specialist profiles across bat and mammalian ACE2 libraries, the research highlights RhGB07 as a useful model for understanding receptor specialization and its implications for sarbecovirus ecology, cross-species transmission, and spillover risk. However, the structural and functional details of RhGB07 receptor specialization remain limited in this study.

Major Comments

This article includes the structural analysis of the RhGB07 Spike protein, and the authors have also attempted to capture the RhGB07 Spike in complex with hACE2 or R. ferrumequinum (Rh.ferr) ACE2. The following points may help strengthen the manuscript:

1.Supplementary Fig. 3: HEK293T cells expressing Rh.ferr ACE2 do not support cell-cell fusion with the RhGB07 Spike protein. Given this, what was the rationale for assembling the Rh.ferr ACE2-RhGB07 Spike complex? It would be helpful to include quantitative binding data between these ACE2 proteins and RhGB07 S-RBD in Supplementary Fig. 3.

2.Cell-cell fusion data: Please provide the original cell-cell fusion images in the Supplementary Figures for clarity and reproducibility.

3.The reported C3-symmetric RBD-down structure of the RhGB07 Spike represents the only conformation obtained. Were any small-molecule densities—such as lipids or biliverdin—observed in the RBD or NTD domains?

4.If only a single RhGB07 Spike structure was obtained, then when comparing RBD conformations in Fig. 2B, the locked SARS-CoV-2 and SARS-CoV-1 RBD structures would be more appropriate controls than the SARS-CoV-2 RBD-ACE2 complex (PDB: 6M0J).

5.Supplementary Fig. 5: The FSC curves do not drop to zero. Please check the Nyquist limit or consider removing duplicate particles.

6.Regarding the monoclonal antibodies isolated from SARS-CoV-2 breakthrough infections: Why does the group 2 antibody VA14_26 broadly bind BANALs, WIV1, Rs4231, and Rc-o319, but not RhGB07 or RfGB02? Given that Rc-o319 has a nine-amino acid deletion in its RBD, where exactly does this antibody bind? Interestingly, mAb VA14_26 shows no binding to RhGB07 in the ELISA assay (Fig. 2E), yet it neutralizes RhGB07 pseudoviruses (Fig. 7B, second panel). This discrepancy should be clarified.

7.Lines 1052-1053: In the description, "The Cα trace of the RBD of RhGB07 rendered as balls and sticks superimposed with the RBD of PRD-0038 apo or PRD-0039 bound to R. alcyone ACE2," the label "PRD-0039" should be corrected to "PRD-0038." Additional structural alignments of clade 3 RBDs would further strengthen the analysis.

8.The authors have missed recent articles analyzing Rc-o319 specificity (https://doi.org/10.1016/j.celrep.2025.116220;https://doi.org/10.1101/2025.09.17.676762), Rc-o319 has a very different RBM as shown by the new structures. The authors should discuss these new results and cross-reference the Rc-o319 binding data in the two articles. In addition, the authors missed a recent article analyzing how non-human ACE2 binder can acquire high-affinity human ACE2 binding (DOI: 10.1126/sciadv.adr8772). This article shows that high-affinity ACE2 binding needs optimal interactions across 4 different ACE2 binding interfaces within the RBM for specific ACE2 molecules. If optimal interactions are found in 3-4 interfaces, it will result in high-affinity binding. Whereas optimal interactions are found in ~2 interfaces, it will result in weak to moderate ACE2 binding. For example, clade 3 RBD BtKY72, with a small loop deletion, can bind R.a-ACE2 at a moderate affinity due to good interactions in 3 interfaces, even if the small loop interface is completely missing. For clade 1, although without deletions in the RBM, interactions in the 4 interfaces for specific ACE2 may not be optimal, therefore could result in varied affinity. The authors should discuss Sarbecovirus ACE2 binding specificity in light of these findings in previous works.

Minor Typographical and Editorial Corrections

*Line 56: "cluster" → "clusters" (plural verb agreement; "analyses" is plural).

*Line 69: Add a comma after "etc." or use "and others," e.g., "(BANAL-20-52, BANAL-20-103, etc.), raising intriguing questions…"

*Line 729: If the RBD of SARS-CoV-2 is used as the control, it should indicate that Rc-o319 lacks 9 amino acids in its RBD.

------------------------------------

Reviewer #3:

The manuscript by Thakur et al. addresses receptor tropism across sarbecoviruses and the antigenic landscape of SARS-CoV-2 immunity. The paper is clearly written and the central message is well conveyed. However, much of the background on ACE2 usage and species-specific receptor compatibility among SARS-CoV-2 and bat sarbecoviruses has been reported previously (e.g., Emerging Infectious Diseases 31:8 (2024), Nat Commun 13:3868 (2022), Sig Transduct Target Ther 5:282 (2020), Cell 184:4203 (2021), Nature 609:597 (2022)). Similarly, cross-reactive neutralization by SARS-CoV-2-elicited antibodies across sarbecoviruses has been documented (Nat Microbiol 7:1867 (2022), Commun Biol (2025), Cell (2024), Nature 597:103 (2021), Sci Transl Med (2024)). In my view, the principal novelty here lies in the structural resolution of RhGB07 Spike and the downstream functional characterization: quantitative binding to diverse ACE2 orthologs and a defined neutralization-escape profile for RhGB07.

To strengthen the manuscript’s contribution, I have following recommendation:

Resolve the structure of RhGB07 spike with Rh. alcyone ACE2.

What is the binding affinity of RhGB07 RBD for Rh. alcyone ACE2, compared to ACE2 from different species?

How did the authors normalise the entry data since the pseudovirus packaging efficiency varies between sarbecovirus spikes, as shown in Supplementary Fig. 2b?

Based on the data in Fig. 1 and Fig. 3, SARS-CoV-1 should be regarded as a generalist, and I was wondering why the authors excluded SARS-CoV-1 from the antigenicity characterization?

Most of the sarbecoviruses selected in this study are closely related to SARS-CoV-2; thus, a high level of cross-neutralization activity is expected and has been previously demonstrated. It would be nice if the authors could include more SARS-CoV-1–related animal CoVs in this study, such as those from different subclades in Fig. 1a (e.g., YN2018B, Rs4874, LYRa11, RsSHC014).

Line 691–693: This statement may not be true since more sarbecoviruses are yet to be discovered, and this study does not include all known sarbecoviruses. As observed in SARS-CoV-2 evolution, early SARS-CoV-2 vaccine-induced neutralizing antibodies failed to neutralize existing circulating variants, e.g., NB.1.8.1 or XFG.

Minor comments:

RaTG13 RBD carries 22 amino acid differences from SARS-CoV-2; in my personal view, RaTG13 is quite different from SARS-CoV-2.

------------------------------------

Reviewer #4:

Here, Thakur, Newman, and colleagues explore the host usage and antigenicity of bat coronavirus spike proteins, focusing on ACE2-using viruses. They describe two viral phenotypes - a generalist and a specialist - and characterize the molecular structure features of receptor-spike interactions using various assays including binding and pseudovirus entry assays. They also report a cryo-EM structure of the RHGB07 spike glycoprotein ectodomain in its apo form. Finally, the authors examine the antigenicity of the different viral spike receptor-binding domains in the context of human serum antibody binding. Overall, the authors point to the generalist ACE2-using bat sarbecoviruses being structurally and antigenically very similar to SARS-CoV-2.

The manuscript reports a wealth of technically-sound functional data, but it is, overall, a very descriptive analysis as there is little correlation observed within and across the generalist and the specialist virus types.

Additionally, major caveats stem from the structural analyses of only the apo RhGB07 spiek structure.

Major critiques:

1 - structure determination of RhGB07: the data processing details in Figure S5 report over 2M particles picked with template picker, yet only a fraction of those (20K) went into the final 3D reconstruction and refinement.

a)If you are using only 1% of the initial particle stack, then surely, you are missing alternative conformations, other molecular species etc. The data should be carefully re-processed.

b)Refinement is only done with imposed C3 symmetry and, therefore, any RBD up/down variations would have been missed due to averaging.

c)Supposedly, ACE2 was included in the sample, but was not detected on cryo-EM grids. Was ACE2 excluded from the grid holes? Is there evidence of it being in the holes but dissociated? Please provide raw micrograph images and additional 2D processing with, for example, smaller particle picking params.

d)In the same vein, the authors should show physical interaction with bat ACE2 and RhGB07 spike using size-exclusion chromatography and additional biophysical methods.

e)Atomic model validation is missing

2 - Epitope binning in Figure 6 should be done with Fabs and not full IgG molecules because of steric hindrance on such a small protein domain, as is RBD.

Minor:

1 - which PDBs were used for Figure 2B? Is it fair to compare apo RBD structures with those complexed with ACE2 and draw conclusions about crucial binding loop rearrangements?

# Author comment: Author response to Decision Letter 2

**DOI:** 10.1371/journal.pbio.3003944.r003

11 May 2026

Attachment Submitted filename: PBIOLOGY-D-25-03032R1_Response to reviewers.pdf

# Peer review: Decision Letter 2

**DOI:** 10.1371/journal.pbio.3003944.r004

**Authors:** Melissa Vazquez Hernandez

15 Jun 2026

Dear Dr Thakur,

Thank you for your patience while we considered your revised manuscript "Understanding the future risk of bat coronavirus spillover into humans – correlating sarbecovirus receptor usage, host range, and antigenicity." for consideration as a Research Article at PLOS Biology. Your revised study has now been evaluated by the PLOS Biology editors, the Academic Editor and the original reviewers.

As you will see from the reports, the reviewers were generally positive about the revised manuscript and appreciated the additional data and clarification provided. However, there are still several outstanding points that must be addressed before we can further consider the manuscript for publication in PLOS Biology. Specifically, in response to Reviewer #2, please clarify whether the proposed explanation for the RhGB07–R. ferrumequinum ACE2 binding/entry discrepancy is speculative unless sequence identity data are provided, justify or revise the rationale for the RhGB07–R. ferrumequinum ACE2 structural complex, further support the explanation for the truncated FSC curve, and please provide representative raw SPR sensorgrams in the Supplementary Information. In response to Reviewer #3, please better justify the exclusion of SARS-CoV-1 RBD and, where possible, support ACE2-binding claims with quantitative BLI or SPR affinity measurements rather than ELISA alone. Addressing these remaining points will be important for our further consideration of the manuscript.

In light of the reviews, which you will find at the end of this email, we are pleased to offer you the opportunity to address the remaining points from the reviewers in a revision that we anticipate should not take you very long. We will then assess your revised manuscript and your response to the reviewers' comments with our Academic Editor aiming to avoid further rounds of peer-review, although we might need to consult with the reviewers, depending on the nature of the revisions.

In addition to these revisions, you may need to complete some formatting changes, which you will receive in a follow up email. A member of our team will be in touch with a set of requests shortly. If you do not receive a separate email within a few days, please assume that checks have been completed, and no additional changes are required.

We expect to receive your revised manuscript within 1 month. Please email us (plosbiology@plos.org) if you have any questions or concerns, or would like to request an extension.

At this stage, your manuscript remains formally under active consideration at our journal; please notify us by email if you do not intend to submit a revision so that we withdraw the manuscript.

**IMPORTANT - SUBMITTING YOUR REVISION**

Your revisions should address the specific points made by each reviewer. Please submit the following files along with your revised manuscript:

1. A 'Response to Reviewers' file - this should detail your responses to the editorial requests, present a point-by-point response to all of the reviewers' comments, and indicate the changes made to the manuscript.

*NOTE: In your point-by-point response to the reviewers, please provide the full context of each review. Do not selectively quote paragraphs or sentences to reply to. The entire set of reviewer comments should be present in full and each specific point should be responded to individually.

You should also cite any additional relevant literature that has been published since the original submission and mention any additional citations in your response.

2. In addition to a clean copy of the manuscript, please also upload a 'track-changes' version of your manuscript that specifies the edits made. This should be uploaded as a "Revised Article with Changes Highlighted " file type.

*Resubmission Checklist*

When you are ready to resubmit your revised manuscript, please refer to this resubmission checklist: https://plos.io/Biology_Checklist

To submit a revised version of your manuscript, please go to https://www.editorialmanager.com/pbiology/ and log in as an Author. Click the link labelled 'Submissions Needing Revision' where you will find your submission record.

Please make sure to read the following important policies and guidelines while preparing your revision:

*Published Peer Review*

Please note while forming your response, if your article is accepted, you may have the opportunity to make the peer review history publicly available. The record will include editor decision letters (with reviews) and your responses to reviewer comments. If eligible, we will contact you to opt in or out. Please see here for more details:

https://blogs.plos.org/plos/2019/05/plos-journals-now-open-for-published-peer-review/

*PLOS Data Policy*

Please note that as a condition of publication PLOS' data policy (http://journals.plos.org/plosbiology/s/data-availability) requires that you make available all data used to draw the conclusions arrived at in your manuscript. If you have not already done so, you must include any data used in your manuscript either in appropriate repositories, within the body of the manuscript, or as supporting information (N.B. this includes any numerical values that were used to generate graphs, histograms etc.). For an example see here: http://www.plosbiology.org/article/info%3Adoi%2F10.1371%2Fjournal.pbio.1001908#s5

*Blot and Gel Data Policy*

We require the original, uncropped and minimally adjusted images supporting all blot and gel results reported in an article's figures or Supporting Information files. We will require these files before a manuscript can be accepted so please prepare them now, if you have not already uploaded them. Please carefully read our guidelines for how to prepare and upload this data: https://journals.plos.org/plosbiology/s/figures#loc-blot-and-gel-reporting-requirements

*Protocols deposition*

To enhance the reproducibility of your results, we recommend that if applicable you deposit your laboratory protocols in protocols.io, where a protocol can be assigned its own identifier (DOI) such that it can be cited independently in the future. Additionally, PLOS ONE offers an option for publishing peer-reviewed Lab Protocol articles, which describe protocols hosted on protocols.io. Read more information on sharing protocols at https://plos.org/protocols?utm_medium=editorial-email&utm_source=authorletters&utm_campaign=protocols

Thank you again for your submission to our journal. We hope that our editorial process has been constructive thus far, and we welcome your feedback at any time. Please don't hesitate to contact us if you have any questions or comments.

Sincerely,

Melissa

Melissa Vazquez Hernandez, Ph.D.

Associate Editor

PLOS Biology

mvazquezhernandez@plos.org

----------------------------------------------------------------

REVIEWERS' COMMENTS

----------------------------------------------------------------

Reviewer #1:

The authors have added substantial data to this manuscript that satisfies all of my previous comments.

----------------------------------------------------------------

Reviewer #2:

I appreciate the authors' efforts in revising the manuscript and addressing the concerns raised in the previous review. In particular, the newly added Δ4-loop mutagenesis and ELISA assays provide important functional support for the structural findings. The inclusion of cell-cell fusion data, comparisons with recent clade 3 sarbecovirus structures, and an expanded discussion of ACE2-binding interfaces and receptor tropism further improve the clarity and context of the study.

I have only a few minor comments that the authors may wish to consider prior to publication:

1. RhGB07-R. ferrumequinum ACE2 interaction and complex

The authors suggest that differences between UK and Chinese R. ferrumequinum ACE2 proteins may explain the discrepancy between binding and entry phenotypes. What is the sequence identity between these ACE2 proteins? Without sequence information, authors should explicitly say this explanation is speculative.

In addition, RhGB07 did not mediate detectable pseudovirus entry or cell-cell fusion through R. ferrumequinum ACE2 in the current study. Therefore, the rationale for pursuing structural characterization of this complex remains unclear. Remove this statement if possible.

2. FSC curve

The explanation that the FSC curve is truncated due to particle cropping is reasonable. However, this remains an interpretation rather than a direct validation. I suggest re-extracting the final particles without Fourier cropping and performing a final refinement. If the FSC curve then decays toward zero while maintaining a comparable resolution estimate, this would directly confirm the authors' explanation.

3. SPR data transparency

The explanation regarding the apparent discrepancy between ELISA, SPR, and neutralization data is appreciated. However, the interpretation remains difficult to evaluate from summarized KD, EC50, and IC50 values alone.

I recommend providing the raw SPR sensorgrams for representative antibodies, particularly VA14_26 against WIV1, BANAL viruses, Rc-o319, and RhGB07, in the Supplementary Information. These data would allow readers to better assess the quality of the fits and reconcile the ELISA, SPR, and neutralization datasets.

Overall, the manuscript has been substantially strengthened and minor improvement may be needed for publication.

----------------------------------------------------------------

Reviewer #3:

The authors have adequately addressed my concerns.

1) However, I was somewhat surprised that the authors were unable to purify the SARS-CoV-1 RBD, given that there are many commercial sources available for this protein. Therefore, this may not be a sufficiently strong justification for excluding it from the current study. Although SARS-CoV-1, WIV-1, and Rs4231 all belong to clade 1a sarbecoviruses, they are still phylogenetically and antigenically distinct from one another.

2) Although the authors have provided ELISA data for RhGB07 RBD binding to Rh. alcyone ACE2, as well as Kd values for RhGB07 RBD interactions with other bat and human ACE2 receptors published elsewhere, ELISA assays do not directly measure binding affinity. Therefore, it would be preferable for the authors to perform biolayer interferometry (BLI) or surface plasmon resonance (SPR) assays to accurately determine the binding affinities.

# Author comment: Author response to Decision Letter 3

**DOI:** 10.1371/journal.pbio.3003944.r005

7 Jul 2026

Attachment Submitted filename: Response to reviewers_R2.docx

# Editor report: Decision Letter 3

**DOI:** 10.1371/journal.pbio.3003944.r006

**Authors:** Melissa Vazquez Hernandez

22 Jul 2026

Dear Nazia,

Thank you for your patience while we considered your revised manuscript "Understanding the future risk of bat coronavirus spillover into humans – correlating sarbecovirus receptor usage, host range, and antigenicity." for publication as a Research Article at PLOS Biology. This revised version of your manuscript has been evaluated by the PLOS Biology editors and the Academic Editor.

Based on our Academic Editor's assessment of your revision, we are likely to accept this manuscript for publication, provided you satisfactorily address the following data and other policy-related requests.

1) We routinely suggest changes to titles to ensure maximum accessibility for a broad, non-specialist readership, and to ensure they reflect the contents of the paper. Please ensure you change both the manuscript file and the online submission system, as they need to match for final acceptance:

"ACE2 receptor breadth predicts host range and antigenic relatedness across sarbecoviruses"

2) Please add weblinks of the funding agencies in the Financial Disclosure statement in the manuscript details.

3) Thank you for providing the raw data. Please cite the location of the data clearly in all relevant main and supplementary Figure legends, e.g. “The data underlying this Figure can be found in S1 Data” or “The data underlying this Figure can be found in https://doi.org/10.5281/zenodo.XXXXX”

4) We require the original, uncropped and minimally adjusted images supporting all blot and gel results reported in the Figures S2BC

-- We will require these files before a manuscript can be accepted so please prepare and upload them now. Please carefully read our guidelines for how to prepare and upload this data: https://journals.plos.org/plosbiology/s/figures#loc-blot-and-gel-reporting-requirements

5) Please provide the tree files for the phylogenetic trees in Figures 1A, 4A, S11A. Please make sure all relevant figures have scale bars.

6) We ask that you please make the structures publicly available at this stage before publication. Also note that we require the code for the structural analyses.

7) For figures containing FACS data (Figures S5A), please provide the FCS files and a picture showing the successive plots and gates that were applied to the FCS files to generate the figure. We ask that you please deposit this data an open repository like Zenoso and provide the accession number/URL of the deposition in the Data Availability Statement in the online submission form.

8) Supplementary files (e.g., excel). Please ensure that all data files are uploaded as 'Supporting Information' and are invariably referred to (in the manuscript, figure legends, and the Description field when uploading your files) using the following format verbatim: S1 Data, S2 Data, etc. Multiple panels of a single or even several figures can be included as multiple sheets in one excel file that is saved using exactly the following convention: S1_Data.xlsx (using an underscore).

9) Please ensure that your Data Statement in the submission system accurately describes where your data can be found and is in final format, as it will be published as written there

10) Per journal policy, if you have generated any custom code during the course of this investigation, please make it available without restrictions. Please ensure that the code is sufficiently well documented and reusable, and that your Data Statement in the Editorial Manager submission system accurately describes where your code can be found. More information on our Code Policy, what and how to share can be found here: https://journals.plos.org/plosbiology/s/code-availability

Please note that we cannot accept sole deposition of code in GitHub, as this could be changed after publication. However, you can archive this version of your publicly available GitHub code to Zenodo. Once you do this, it will generate a DOI number, which you will need to provide in the Data Accessibility Statement (you are welcome to also provide the GitHub access information). See the process for doing this here: https://docs.github.com/en/repositories/archiving-a-github-repository/referencing-and-citing-content

As you address these items, please take this last chance to review your reference list to ensure that it is complete and correct. If you have cited papers that have been retracted, please include the rationale for doing so in the manuscript text, or remove these references and replace them with relevant current references. Any changes to the reference list should be mentioned in the cover letter that accompanies your revised manuscript.

In addition to these revisions, you may need to complete some formatting changes, which you will receive in a follow up email. A member of our team will be in touch with a set of requests shortly. If you do not receive a separate email within a few days, please assume that checks have been completed, and no additional changes are required.

We expect to receive your revised manuscript within two weeks.

To submit your revision, please go to https://www.editorialmanager.com/pbiology/ and log in as an Author. Click the link labelled 'Submissions Needing Revision' to find your submission record. Your revised submission must include the following:

- a cover letter that should detail your responses to any editorial requests, if applicable, and whether changes have been made to the reference list

- a Response to Reviewers file that provides a detailed response to the reviewers' comments (if applicable, if not applicable please do not delete your existing 'Response to Reviewers' file.)

- a track-changes file indicating any changes that you have made to the manuscript.

NOTE: If Supporting Information files are included with your article, note that these are not copyedited and will be published as they are submitted. Please ensure that these files are legible and of high quality (at least 300 dpi) in an easily accessible file format. For this reason, please be aware that any references listed in an SI file will not be indexed. For more information, see our Supporting Information guidelines:

https://journals.plos.org/plosbiology/s/supporting-information

*Published Peer Review History*

Please note that you may have the opportunity to make the peer review history publicly available. The record will include editor decision letters (with reviews) and your responses to reviewer comments. If eligible, we will contact you to opt in or out. Please see here for more details:

https://plos.org/published-peer-review-history/

*Press*

Should you, your institution's press office or the journal office choose to press release your paper, please ensure you have opted out of Early Article Posting on the submission form. We ask that you notify us as soon as possible if you or your institution is planning to press release the article.

*Protocols deposition*

To enhance the reproducibility of your results, we recommend that if applicable you deposit your laboratory protocols in protocols.io, where a protocol can be assigned its own identifier (DOI) such that it can be cited independently in the future. Additionally, PLOS ONE offers an option for publishing peer-reviewed Lab Protocol articles, which describe protocols hosted on protocols.io. Read more information on sharing protocols at https://plos.org/protocols?utm_medium=editorial-email&utm_source=authorletters&utm_campaign=protocols

Please do not hesitate to contact me should you have any questions.

Sincerely,

Melissa

Melissa Vazquez Hernandez, Ph.D.

Senior Editor

mvazquezhernandez@plos.org

PLOS Biology

# Author comment: Author response to Decision Letter 4

**DOI:** 10.1371/journal.pbio.3003944.r007

25 Jul 2026

Attachment Submitted filename: PBIOLOGY-D-25-03032R1_Response to reviewers_collated.docx

# Editor report: Decision Letter 4

**DOI:** 10.1371/journal.pbio.3003944.r008

**Authors:** Melissa Vazquez Hernandez

28 Jul 2026

Dear Nazia,

Thank you for the submission of your revised Research Article "ACE2 receptor breadth predicts host range and antigenic relatedness across bat sarbecoviruses." for publication in PLOS Biology. On behalf of my colleagues and the Academic Editor, Ali Ellebedy, I am pleased to say that we can in principle accept your manuscript for publication, provided you address any remaining formatting and reporting issues. These will be detailed in an email you should receive within 2-3 business days from our colleagues in the journal operations team; no action is required from you until then. Please note that we will not be able to formally accept your manuscript and schedule it for publication until you have completed any requested changes.

FYI that we have commissioned a primer to bring context to both papers. We will reach out as soon as it is submitted for your opinion.

Please take a minute to log into Editorial Manager at http://www.editorialmanager.com/pbiology/, click the "Update My Information" link at the top of the page, and update your user information to ensure an efficient production process.

PRESS

We frequently collaborate with press offices. If your institution or institutions have a press office, please notify them about your upcoming paper at this point, to enable them to help maximise its impact. If the press office is planning to promote your findings, we would be grateful if they could coordinate with biologypress@plos.org. If you have previously opted in to the early version process, we ask that you notify us immediately of any press plans so that we may opt out on your behalf.

We also ask that you take this opportunity to read our Embargo Policy regarding the discussion, promotion and media coverage of work that is yet to be published by PLOS. As your manuscript is not yet published, it is bound by the conditions of our Embargo Policy. Please be aware that this policy is in place both to ensure that any press coverage of your article is fully substantiated and to provide a direct link between such coverage and the published work. For full details of our Embargo Policy, please visit http://www.plos.org/about/media-inquiries/embargo-policy/.

Thank you again for choosing PLOS Biology for publication and supporting Open Access publishing. We look forward to publishing your study.

Sincerely,

Melissa

Melissa Vazquez Hernandez, Ph.D., Ph.D.

Senior Editor

PLOS Biology

mvazquezhernandez@plos.org
